# Synthesis and Biological Evaluation of Harmirins, Novel Harmine–Coumarin Hybrids as Potential Anticancer Agents

**DOI:** 10.3390/molecules26216490

**Published:** 2021-10-27

**Authors:** Kristina Pavić, Maja Beus, Goran Poje, Lidija Uzelac, Marijeta Kralj, Zrinka Rajić

**Affiliations:** 1 Faculty of Pharmacy and Biochemistry, University of Zagreb, 10 000 Zagreb, Croatia; kpavic@pharma.hr (K.P.); mbeus@pharma.hr (M.B.); gpoje@pharma.hr (G.P.); 2 Laboratory of Experimental Therapy, Division of Molecular Medicine, Ruđer Bošković Institute, 10 000 Zagreb, Croatia; Lidija.Uzelac@irb.hr (L.U.); Marijeta.kralj@irb.hr (M.K.)

**Keywords:** harmine, β-carboline, coumarin, triazole, antiproliferative activity, cell cycle analysis

## Abstract

As cancer remains one of the major health burdens worldwide, novel agents, due to the development of resistance, are needed. In this work, we designed and synthesized harmirins, which are hybrid compounds comprising harmine and coumarin scaffolds, evaluated their antiproliferative activity, and conducted cell localization and cell cycle analysis experiments. Harmirins were prepared from the corresponding alkynes and azides under mild reaction conditions using Cu(I) catalyzed azide–alkyne cycloaddition, leading to the formation of the 1*H*-1,2,3-triazole ring. Antiproliferative activity of harmirins was evaluated in vitro against four human cancer cell lines (MCF-7, HCT116, SW620, and HepG2) and one human non-cancer cell line (HEK293T). The most pronounced activities were exerted against MCF-7 and HCT116 cell lines (IC_50_ in the single-digit micromolar range), while the most selective harmirins were **5b** and **12b**, substituted at C-3 and O-7 of the β-carboline core and bearing methyl substituent at position 6 of the coumarin ring (SIs > 7.2). Further experiments demonstrated that harmirin **12b** is localized exclusively in the cytoplasm. In addition, it induced a strong G1 arrest and reduced the percentage of cells in the S phase, suggesting that it might exert its antiproliferative activity through inhibition of DNA synthesis, rather than DNA damage. In conclusion, harmirin **12b** is a novel harmine and coumarin hybrid with significant antiproliferative activity and warrants further evaluation as a potential anticancer agent.

## 1. Introduction

Cancer remains one of the greatest global health burdens, affecting an estimated 19.3 million new patients and causing nearly 10 million deaths in 2020. The most commonly diagnosed cancers are breast, lung, colorectal, prostate, and stomach, with lung cancer being the leading cause of cancer death [1]. In 2020/2021, the diagnosis and treatment of cancer were negatively affected by the COVID-19 pandemic. This may have led to a false decline in cancer incidence, but the true impact of delays in diagnosis and treatment will only become apparent in subsequent years [2]. At the same time, the silent pandemic of anticancer drug resistance is developing in the background, leading to cancer recurrence and treatment failures [3]. Therefore, there is still a constant need for new effective anticancer agents.

There are many approaches in drug discovery and development (e.g., follow-on and analog-based drug discovery, product-based research, drug repositioning, and repurposing) [4]. The molecular hybridization approach, a strategy in which two molecules/pharmacophores are combined to form a new hybrid compound, is a useful tool for drug development targeting multifactorial diseases, including cancer. Due to the possible involvement of different or dual/multiple modes of action, the “combined chemotherapy-like” effect of anticancer hybrids is achieved, while overcoming the drawbacks of conventional chemotherapeutic agents [5,6,7,8,9]. 

**Figure 1:**
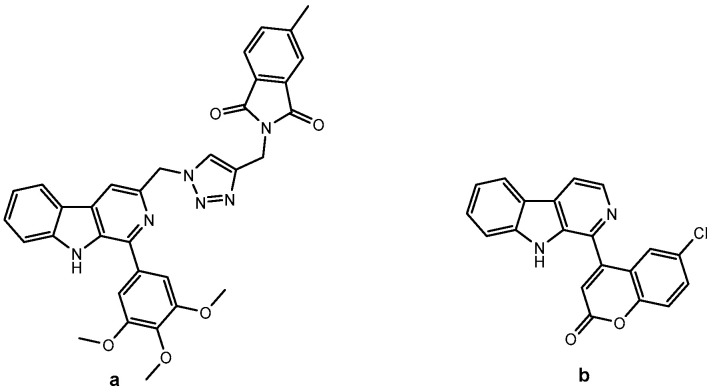
β-carboline hybrids with anticancer activity: (**a**) β-carboline–triazole hybrid (IC_50_ = 3.67 and 5.44 µM against HT-29 and HGC-27) [16], (**b**) coumarin–β-carboline hybrid (GI_50_ = 67.5 µM against HeLa) [23].

β-carbolines and coumarins are two important classes of pharmacologically active natural products used extensively in medicinal chemistry. Harmine, the best-known representative of the β-carboline alkaloids, is found in the seeds and roots of *Peganum harmala*, Nitrariaceae. In general, β-carbolines possess a wide range of biological activities, such as anticancer, anti-inflammatory, antiviral, antiparasitic, hallucinogenic, etc. [3,5,10,11,12,13]. Today, the abundance of research in this field focuses on their anticancer properties. They have been shown to interact with various anticancer drug targets, such as DNA (intercalation [3,14,15], groove binding [3,16]), topoisomerase [3,17], kinases [3,18,19], and α-tubulin [3,20]. Several series of β-carboline–triazole hybrids have been synthesized and evaluated for their antiproliferative activity [5]: C3-linked β-carboline–triazole conjugates (an example is shown in Figure 1a) [16], C1-linked β-carboline–triazoles with an aryl-alkyl ether spacer [21], and tetrahydro-β-carboline–chalcone hybrids [22].

Coumarins are phytochemicals that belong to a family of benzopyran-2-ones [24] and have outstanding therapeutic potential (anticoagulant, antimicrobial, anticancer, anti-inflammatory, etc.) [9]. They have been shown to inhibit kinases, aromatase, heat shock protein 90 (Hsp90), telomerase, angiogenesis and can cause cell cycle arrest [8,25]. Novobiocin, an aminocoumarin antibiotic, and its analogs inhibit Hsp90 via the ubiquitin-proteasome pathway [26]. Clausarin, another naturally occurring coumarin, showed superior antiproliferative activity compared to cisplatin against HepG2, HCT116, and SK-LU-1 cancer cell lines [27]. Samundeeswari et al. reported the synthesis and antimitotic activity of a series of coumarin–β-carboline hybrids in which the coumarin moiety was directly linked to the β-carboline core at the C-1 position. Hybrids with fully aromatized β-carboline ring and C-6 substituted coumarin moiety showed better activity against several cancer cell lines than the corresponding tetrahydro analogs (the most active is shown in Figure 1b) [23].

**Figure 2:**
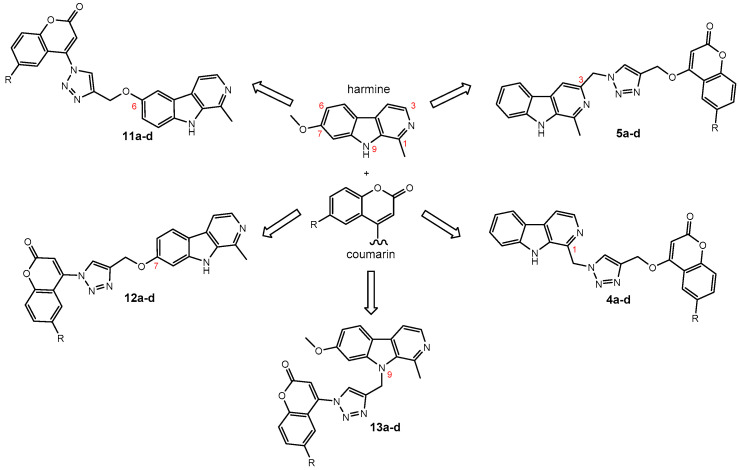
Novel harmirins—harmine–coumarin hybrids.

We have employed the concept of molecular hybridization to develop harmirins—novel compounds combining harmine and coumarin pharmacophores in hybrid molecules linked by a 1*H*-1,2,3-triazole spacer (Figure 2). In this paper, we report their synthesis, antiproliferative activity, cell localization, and influence on the cell cycle.

## 2. Results and Discussion

### 2.1. Chemistry

**Scheme 1:**
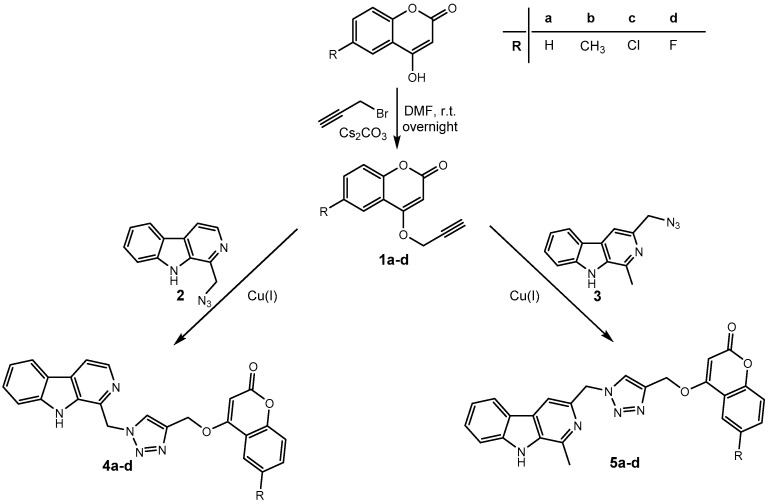
Synthesis of harmirins **4** and **5**.

**Scheme 2:**
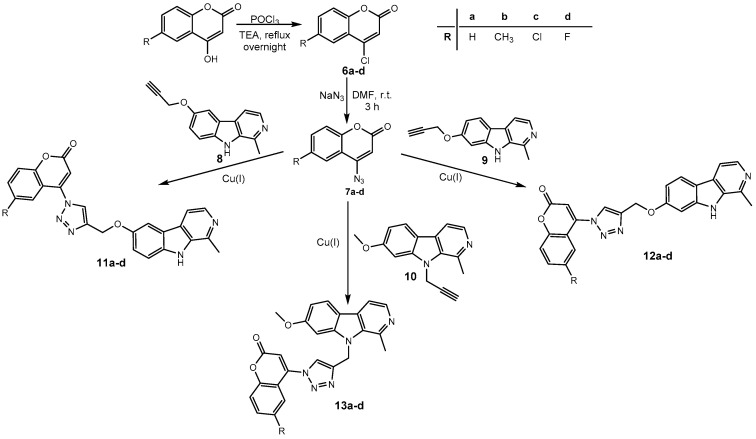
Synthesis of harmirins **11**–**13**.

Harmirins, harmine–coumarin hybrids, were obtained by applying the standard Cu(I) catalyzed azide–alkyne cycloaddition (CuAAC), leading to the formation of a 1*H*-1,2,3-triazole ring (Scheme 1 and Scheme 2). Triazole was selected as a suitable linker between two bioactive moieties due to its chemical inertness to oxidation, reduction, and hydrolysis under acidic or basic conditions. Moreover, triazole is an excellent bioisostere of the amide bond [28]. The structural diversity of the title compounds was achieved by the following measures: (1) five series of harmirins were synthesized (**4a**–**d**, **5a**–**d**, **11a**–**d**, **12a**–**d**, and **13a**–**d**), which differ by the position of the coumarin-based substituents on the β-carboline core, viz., 1, 3, 6, 7, and 9; (2) in each series, four different substituents at position 6 of the coumarin ring (-H, -CH_3_, -Cl, -F) were varied; (3) the harmirins **4** and **5** bear a methyleneoxy spacer between the triazole and coumarin heterocycles; and (4) the triazole ring is directly attached to the coumarin moiety in derivatives **11**–**13**.

The required starting compounds for CuAAC were harmine- and coumarin-based azides, **2**, **3**, and **7a**–**d**, as well as coumarin- and harmine-based terminal alkynes, **1a**–**d** and **8**–**10**. The treatment of 4-hydroxycoumarins with propargyl bromide in the presence of cesium carbonate as a base gave *O*-alkylated coumarins **1a**–**d** in a one-step reaction. Coumarin-based azides **7a**–**d** were prepared from 4-hydroxycoumarins in a two-step procedure [29]. The first step involved the chlorination of 4-hydroxycoumarins with phosphorous(V) oxychloride. The obtained 4-chlorocoumarins **6a**–**d** were then converted to azides **7a**–**d** using sodium azide. The harmine-based azides **2** and **3** and the harmine-based terminal alkynes **8**–**10** were prepared according to our previously published procedure [30,31,32].

In the next step, harmirins were efficiently prepared by CuAAC under mild reaction conditions and in relatively good yields (26–69%). Two general methods were employed for the generation of an active catalyst, Cu(I) cations. Most of the harmirins were prepared using Cu(II) acetate precatalyst in methanol as a reducing agent. On the other hand, sodium ascorbate was the reducing agent of choice for the generation of Cu(I) from CuSO_4_ × 5H_2_O in the synthesis of harmirins **4a**–**d** due to the lower number of by-products and easier purification. Almost all reactions proceeded at room temperature. However, in some cases, the reactions were slow, the yields were poor or did not proceed at all. The use of microwave-assisted synthesis significantly shortened reaction times, reduced the formation of by-products, and increased yields in the synthesis of several 6- and 9-substituted harmirins (**11b**,**d** and **13d**, respectively). On the contrary, harmirin **11c** was obtained by a classical synthetic procedure at 50 °C due to the formation of by-products during the microwave-assisted synthesis. The reaction conditions and obtained yields are summarized in Table 1.

In total, we prepared 20 harmirins, which were characterized by the standard spectroscopic/spectrometric methods (IR, MS, ^1^H and ^13^C-APT NMR). The spectral data were in agreement with the proposed structures and are presented briefly in the Materials and Methods section and in detail in the Supporting Information. The formation of the triazole ring was confirmed by the presence of a characteristic singlet in ^1^H NMR in the region of 8.46–9.02 ppm due to triazolyl proton. The corresponding carbon atom appeared at 124.67–126.54 ppm in ^13^C-APT NMR, while the quaternary carbon of the triazole ring was in the region of 137.93–144.75 ppm. The purity of the prepared compounds was evaluated by the elemental analysis, with the values for carbon, hydrogen, and nitrogen within 0.4% of those calculated for the proposed molecular formula.

All synthesized harmirins are in almost complete agreement with Lipinski’s rule of five and Gelovani’s rules for the prospective small molecular drugs (MW ≤ 500, log *P* ≤ 5, number of H-bond donors ≤ 5, number of H-bond acceptors ≤ 10, polar surface area (PSA) < 140 Å^2^, molar refractivity (MR) in the range of 40 and 130 cm^3^/mol). Minimal aberrations of the rules are present only for the MR. The parameters were calculated using the Chemicalize.org program and are shown in Table S13 [33].

### 2.2. Biological Evaluations

#### 2.2.1. Antiproliferative Activity

We selected four different cancer cell lines for antiproliferative screening in vitro, which we believed would provide sufficient data on the anticancer potential of the prepared harmirins (hepatocellular carcinoma—HepG2, colorectal adenocarcinoma, Dukes’ type C—SW620, colorectal carcinoma—HCT116, and breast adenocarcinoma—MCF-7). Additionally, we included one non-cancer cell line (embryonic kidney—HEK293T) to evaluate harmirins’ selectivity. The results obtained are shown in Table 2. The commonly used anticancer drug 5-FU and harmine were used as positive controls. Pre-screening was performed using 50 µM of the tested compound. Only compounds that led to more than 50% reduction in mitochondrial metabolic activity at 50 µM concentration were selected for further analysis. The selectivity index (SI) for each harmirin was calculated as a fractional ratio between the IC_50_ values for HEK293T and the cancer cell line MCF-7 or HCT116 since these two cell lines were the most susceptible to harmirins. 

The most active harmirins exhibited stronger activity against MCF-7 and HCT116 compared to HepG2 and SW620 (the least sensitive cancer cell line). Since the difference in the activity of harmirins was greater against MCF-7 than HCT116 when compared to the activity of the parent compound harmine and 5-FU, further structure–activity relationship (SAR) analyses were performed for MCF-7 (although some interesting results were also obtained for HCT116 and will be discussed later in the text).

The activity of harmirins against MCF-7 decreased according to the pattern: **11** (O-6) > **12** (O-7) > **13** (N-9) > **5** (C-3) > **4** (C-1). Within the series, the compounds differed by the substituents at position 6 of the coumarin ring. The most active compounds **11a**,**d** and **13d**, bearing the smallest substituents, hydrogen or fluorine, showed the highest cytotoxicity at low micromolar concentrations (IC_50_ = 1.9–2.7 μM), which were ~5–7-fold higher than harmine (marked in red in Table 2). Remarkably, their activity against MCF-7 was also ~9–13-fold stronger than the activity of 5-FU. Harmirins **11**, as well as compound **13d**, exerted strong activity against all tested cell lines, including HEK293T. Thus, these compounds exerted low SI. Interestingly, compounds **5** showed stronger activity against HEK293T than against cancer cell lines.

In contrast, the most active compound against MCF-7 within the series of O-7 substituted harmirins, **12b**, bears a methyl substituent (IC_50_ = 6.9 ± 0.7 μM, marked in green in Table 2). Although it is only involved in weak London dispersion interactions, the methyl group has stereoelectronic effects on biomacromolecules that could subsequently lead to increased receptor selectivity, efficacy, and metabolic stability [34]. Moreover, compound **12b** exerted the most selective activity towards MCF-7 (SI > 7.2) compared to HEK293T. Interestingly, the most selective and active compounds against HCT116 were 7- and 3-substituted harmirins with a methyl substituent at position 6 of the coumarin core: **12b** (IC_50_ = 4.7 ± 0.6 µM, SI > 10.6) and **5b** (IC_50_ = 3.2 ± 0.5 µM, SI = 8.6), respectively (marked in green in Table 2).

Compound **12b** and MCF-7 were selected for further investigation due to the following reasons: (1) compound **12b** is among the most active harmirins against MCF-7 and also the most selective one, and (2) the cytotoxicity of **12b** against MCF-7 is twofold higher than harmine and 3.4-fold higher than 5-FU.

#### 2.2.2. Cell Localization

**Figure 3:**
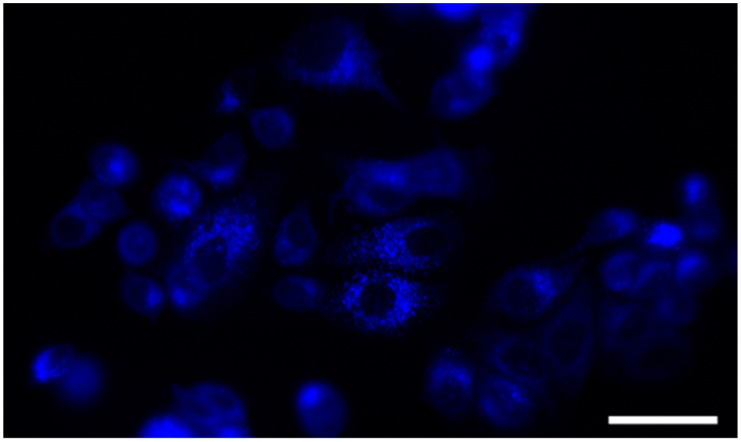
Fluorescent microscopy image of MCF-7 incubated with 10 µM concentration of compound **12b**. The cells were treated for 30 min, rinsed, and analyzed by fluorescent microscope at 400× magnification. Compound **12b** shows predominantly cytoplasmic distribution. The scale bar is 50 µm.

We further examined the intracellular distribution of the compound **12b** in MCF-7 cells, based on its fluorescence properties. Therefore, we incubated MCF-7 cells for 30 min with the tested compound **12b** and analyzed its localization by fluorescence microscopy (Figure 3). No autofluorescence was detected by examining untreated cells under typical imaging conditions. The tested compound showed punctate staining, pointing to the localization within the cytoplasm, but not within the nucleus. The results confirm that **12b** does not bind to the nuclear DNA, i.e., it does not target it, as a potential mechanism of action. 

#### 2.2.3. Cell Cycle Analysis

**Figure 4:**
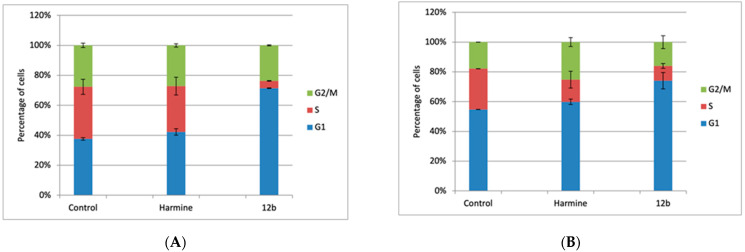
The effect of **12b** and harmine on the cell cycle distribution of MCF-7 cells. Cells were treated with 10 µM of **12b** and 20 µM of harmine for 24 h (**A**) and 48 h (**B**), and then the cell cycle was analyzed by flow cytometry. The histograms represent the percentage of cells in the respective cell cycle phase (G1, S, and G2/M). Average values from three individual experiments ± SD are presented.

To gain further insight into the potential mechanism of activity of **12b**, and to examine whether and how the cell growth inhibition was associated with cell cycle regulation, we assessed its influence on the cell cycle of MCF-7 cells, 24 and 48 h after the treatment and compared it with the influence of the reference compound harmine (Figure 4). Both compounds were tested at the ≈ 1.5 × IC_50_ concentration (10 µM and 20 µM, respectively, obtained in the MTT assay, after 72 h).

As discussed earlier, harmine shows anticancer activity in multiple types of cancer, through various mechanisms, whereby the IC_50_ concentrations differed between the cell lines [35,36]. It is interesting to note that, in our hands, treatment of MCF-7 cells with harmine resulted in an order of magnitude lower IC_50_ value than reported earlier [37]. Harmine also demonstrated antiangiogenic and antitumor effects via the p53 signaling pathway in endothelial cells. These studies clearly demonstrated that in the presence of harmine (10–50 µM), pancreatic cancer cells, as well as human umbilical vein endothelial (HUVEC) cells, were arrested in the G2/M phase of the cell cycle, accompanied by an induction of apoptosis.

Our results show that the treatment with **12b** significantly influenced the cell cycle of MCF-7 cells, already after 24 h. It induced a strong G1 arrest, accompanied by a drastic reduction in the percentage of cells in the S phase, which also persisted after 48 h. The influence of harmine on the cell cycle was much less significant, although a G1 delay, along with the reduction in cells in the S phase, is obvious after 24 h, while after 48 h of treatment, an additional accumulation of cells in the G2/M phase is demonstrated, which is in accordance with the published data. The 48-hour treatment with both compounds resulted in an induction of the accumulation of cells in the subG1 phase (apoptotic cells), up to 10% and 15%, respectively (data not shown).

It could be concluded that **12b** demonstrates a more pronounced influence on the proliferation of breast cancer cell line MCF-7 compared to harmine. However, the mechanism of activity (molecular target) is likely different and should be determined in future studies.

## 3. Materials and Methods

### 3.1. Chemistry

#### 3.1.1. General Information

Melting points were measured on a Stuart Melting Point (SMP3) apparatus (Barloworld Scientific, U.K.) in open capillaries with uncorrected values. FTIR-ATR spectra were recorded using a Fourier-Transform Infrared Attenuated Total Reflection UATR Two spectrometer (PerkinElmer, Waltham, MA, USA) in the range 450–4000 cm^−1^. ^1^H and ^13^C-APT NMR spectra were recorded on a Bruker Avance III HD operating at 300 or 400 MHz for the ^1^H and 75, 101, or 151 MHz for the ^13^C nuclei (Bruker, Billerica, MA, USA). Samples were measured in DMSO-*d*_6_ solutions at 20 °C in 5 mm NMR tubes. Chemical shifts (*δ*) are reported in parts per million (ppm) relative to tetramethylsilane in the ^1^H and the dimethyl sulfoxide (DMSO) residual peak as a reference in the ^13^C spectra (39.51 ppm). Coupling constants (*J*) are reported in Hertz (Hz). Mass spectra were collected on an HPLC-MS/MS instrument (HPLC, Agilent Technologies 1200 Series; MS, Agilent Technologies 6410 Triple Quad, Santa Clara, CA, USA). Mass determination was performed using electrospray ionization (ESI) in a positive mode. Elemental analyses were performed on a CHNS LECO analyzer (LECO Corporation, St. Joseph, MI, USA). Analyses indicated by the symbols of the elements were within ± 0.4% of their theoretical values. Microwave-assisted reactions were performed in a microwave reactor CEM Discover (CEM, Charlotte, NC, USA) in a glass reaction vessel. All compounds were routinely checked by TLC with Merck silica gel 60F-254 glass plates (Merck, Germany) using cyclohexane/ethyl acetate/methanol 3:1:0.75, cyclohexane/ethyl acetate/methanol 3:1:0.5, cyclohexane/ethyl acetate/methanol 1:1:0.5, cyclohexane/ethyl acetate 1:1, dichloromethane/methanol 9.5:0.5, dichloromethane/methanol 9:1, dichloromethane/methanol 7.5:2.5, or dichloromethane/methanol 1:0.1 as solvent systems. Spots were visualized by short-wave (*λ* = 254 nm) and long-wave UV light (*λ* = 366 nm) and iodine vapor. Column chromatography was performed on silica gel 0.063–0.200 mm (Sigma-Aldrich, USA) with the same eluents used for TLC. To eliminate the presence of Cu(I) in the target compounds, a layer of aluminum oxide (90 activated, neutral; 0.063–0.200 mm; Merck, Germany) was applied over silica gel.

All chemicals and solvents were of analytical grade and purchased from commercial sources. Harmine, acetic acid, hydrochloric acid, 4-hydroxycoumarin, acetaldehyde dimethyl acetal, Pd/C (10%), and phosphorus(V) oxychloride were purchased from Sigma-Aldrich (St. Louis, MO, USA). 4-Hydroxy-6-methylcoumarin, 6-chloro-4-hydroxycoumarin, 6-fluoro-4-hydroxycoumarin, cesium carbonate, dimethylformamide (DMF), and trifluoroacetic acid were purchased from Tokyo Chemical Industry (Tokyo, Japan). Hydrobromic acid (47%) and sodium azide were purchased from Merck (Darmstadt, Germany), dichloromethane from Fischer Scientific (Loughborough, U.K.), diethyl ether from ITW Reagents (Darmstadt, Germany), and anhydrous sodium sulfate from Gram-Mol (Zagreb, Croatia). Azides **2** and **3**, as well as alkynes compounds **8**–**10**, were synthesized according to the previously published procedures [30,31,32]. 4-Chlorocoumarins **6a**–**d** and 4-azidocoumarins **7a**–**d** were synthesized according to the slightly modified previously published procedure [29].

#### 3.1.2. General Procedure for the Synthesis of O-alkylated Coumarins 1**a**–**d**

A corresponding 4-hydroxycoumarin (1 mmol) was dissolved in dry DMF (3 mL). Under an argon atmosphere, cesium carbonate (0.456 g, 1.4 mmol) was added, followed by dropwise addition of 80% solution of propargyl bromide in toluene (0.134 mL, 1.2 mmol). The reaction was stirred at r.t. and under argon atmosphere overnight. Upon completion, the reaction mixture was poured into 50 mL water. The product was extracted with dichloromethane (4 × 30 mL). Organic layers were collected and washed with water, dried over anhydrous sodium sulfate, and evaporated under reduced pressure. After trituration with diethyl ether, *O*-alkylated coumarins **1a**–**d** were obtained. Compounds **1a**–**c** were previously described and their analytical data were in accordance with available data [38].

(1) 4-(Prop-2-yn-1-yloxy)-2H-chromen-2-one (**1a**).
  4-Hydroxycoumarin (0.162 g); yield: 0.060 g (30%) of **1a** (white solid).
(2) 6-Methyl-4-(prop-2-yn-1-yloxy)-2H-chromen-2-one (**1b**).
  4-Hydroxy-6-methylcoumarin (0.176 g); yield: 0.062 g (29%) of **1b** (white solid).
(3) 6-Chloro-4-(prop-2-yn-1-yloxy)-2H-chromen-2-one (1**c**).
  6-Chloro-4-hydroxycoumarin (0.197 g); yield: 0.150 g (64%) of **1c** (white solid).
(4) 6-Fluoro-4-(prop-2-yn-1-yloxy)-2H-chromen-2-one (1**d**).

6-Fluoro-4-hydroxycoumarin (0.180 g); yield: 0.092 g (42%) of **1d** (white solid); IR (ATR): ν_max_ 3274, 3237, 3074, 2135, 1702, 1625, 1575, 1492, 1452, 1392, 1360, 1322, 1263, 1221, 1180, 1120, 1083, 993, 932, 884, 818, 743, 712, 662, 593, 521 cm^−1^; ^1^H NMR (400 MHz, DMSO-*d*_6_) *δ* 7.60–7.48 (m, 3H), 6.04 (s, 1H), 5.12 (s, 1H), 3.85 (t, 1H, *J* = 2.39 Hz); ^13^C NMR (101 MHz, DMSO-*d*_6_) *δ* 162.81, 161.08, 158.05 (d, *J* = 242.14 Hz), 149.11, 120.32 (d, *J* = 29.28 Hz), 118.76 (d, *J* = 8.36 Hz), 115.98 (d, *J* = 8.37 Hz), 108.37 (d, *J* = 25.1 Hz), 92.49, 80.33, 57.66; ESI-MS: *m*/*z* calculated for C_12_H_7_FO_3_: 218.0, found 219.0 (M + 1)^+^, 241.0 (M + 23)^+^.

#### 3.1.3. General Procedure for the Synthesis of Harmirins **4a–d**

To a solution of compound **2** (0.039 g, 0.176 mmol) and the corresponding *O*-alkylated coumarin **1a**–**d** (0.160 mmol) in *t*-butanol (4 mL), a solution of sodium ascorbate (10 mg/mL) and CuSO_4_ × 5 H_2_O (20 µL, *c* = 1 M) was added. The reaction mixture was stirred for 0.5–2 h at r.t. The solvent was removed under reduced pressure. The crude product was purified by column chromatography.

(1) 4-((1-((9*H*-Pyrido[3,4-*b*]indol-1-yl)methyl)-1*H*-1,2,3-triazol-4-yl)methoxy)-2*H*-chromen-2-one (**4a**).

From the reaction of **1a** (0.032 g) and after purification by column chromatography (mobile phase dichloromethane/methanol 9.5:0.5) and recrystallization from ethanol, 0.035 g (51%) of an off-white solid **4a** was obtained; reaction time 1 h; mp 260.5–264.0 °C (decomp.); IR (ATR): *ν*_max_ 3283, 1707, 1623, 1609, 1564, 1454, 1434, 1380, 1327, 1273, 1244, 1191, 1141, 1106, 1051, 938, 884, 824, 745, 728, 620 cm^–1^; ^1^H NMR (600 MHz, DMSO-*d*_6_) *δ* 11.99 (s, 1H), 8.51 (s, 1H), 8.31 (d, 1H, *J* = 5.17 Hz), 8.27 (d, 1H, *J* = 7.85 Hz), 8.14 (d, 1H, *J* = 5.14 Hz), 7.73 (dd, 1H, *J* = 1.64, 7.89 Hz), 7.69 (d, 1H, *J* = 8.22 Hz), 7.66-7.64 (ddd, 1H, *J* = 1.65, 7.22, 8.63 Hz), 7.62–7.59 (ddd, 1H, *J* = 1.21, 6.97, 8.22 Hz), 7.41 (dd, 1H, *J* = 1.03, 8.38 Hz), 7.34–7.28 (m, 2H), 6.18, 6.15 (2s, 3H), 5.44 (s, 2H); ^13^C NMR (151 MHz, DMSO-*d*_6_) *δ* 164.37, 161.55, 152.76, 140.87, 140.71, 137.94, 137.88, 133.82, 132.80, 128.74, 128.58, 126.27, 124.21, 122.86, 121.93, 120.74, 119.67, 116.46, 115.08, 114.88, 112.07, 91.34, 62.76, 51.29; ESI-MS: *m*/*z* calculated for C_24_H_17_N_5_O_3_: 423.1, found 424.1 (M + 1)^+^; Anal. Calcd. for C_24_H_17_N_5_O_3_: C, 68.08; H, 4.05; N, 16.54. Found: C, 68.18; H, 4.06; N, 16.56.

(2) 4-((1-((9*H*-Pyrido[3,4-*b*]indol-1-yl)methyl)-1*H*-1,2,3-triazol-4-yl) methoxy)-6-methyl-2*H*-chromen-2-one (**4b**).

From the reaction of **1b** (0.034 g) and after purification by column chromatography (mobile phase dichloromethane/methanol 9.5:0.5) and recrystallization from ethanol, 0.032 g (45%) of a white solid **4b** was obtained; reaction time 0.5 h; mp 246.5–251.0 °C (decomp.); IR (ATR): *ν*_max_ 3337, 3151, 3106, 3059, 1786, 1697, 1626, 1574, 1504, 1430, 1398, 1369, 1330, 1274, 1236, 1212, 1193, 1166, 1126, 1102, 1050, 969, 947, 839, 817, 762, 746, 724, 607, 585, 543, 510 cm^–1^; ^1^H NMR (600 MHz, DMSO-*d*_6_) *δ* 11.99 (s, 1H), 8.50 (s, 1H), 8.31 (d, 1H, *J* = 5.14 Hz), 8.27 (d, 1H, *J* = 7.80 Hz), 8.14 (d, 1H, *J* = 5.16 Hz), 7.68 (d, 1H, *J* = 0.92, 8.24 Hz), 7.62–7.59 (ddd, 1H, *J* = 1.21, 7.03, 8.21 Hz), 7.50 (s, 1H), 7.45 (dd, 1H, *J* = 2.65, 8.82 Hz), 7.30–7.28 (m, 2H), 6.15, 6.14 (2s, 3H), 5.42 (s, 2H), 2.33 (s, 3H); ^13^C NMR (151 MHz, DMSO-*d*_6_) *δ* 164.36, 161.69, 150.92, 140.85, 140.70, 137.93, 137.87, 133.82, 133.62, 133.56, 128.74, 128.58, 126.32, 122.27, 121.93, 120.74, 119.67, 116.28, 114.88, 114.76, 112.07, 91.27, 62.67, 51.28, 20.28; ESI-MS: *m/z* calculated for C_25_H_19_N_5_O_3_: 437.2, found 438.1 (M + 1)^+^; Anal. Calcd. for C_25_H_19_N_5_O_3_: C, 68.64; H, 4.38; N, 16.01. Found: C, 68.74; H, 4.37; N, 16.03.

(3) 4-((1-((9*H*-Pyrido[3,4-*b*]indol-1-yl) methyl)-1*H*-1,2,3-triazol-4-yl) methoxy)-6-chloro-2*H*-chromen-2-one (**4c**).

From the reaction of **1c** (0.038 g) and after purification by column chromatography (mobile phase dichloromethane/methanol 9.5:0.5) and recrystallization from ethanol, 0.051 g (69%) of a white solid **4c** was obtained; reaction time 1 h; mp 263.5–265.0 °C (decomp.); IR (ATR): *ν*_max_ 3253, 3159, 1684, 1616, 1603, 1561, 1485, 1458, 1431, 1359, 1325, 1265, 1229, 1194, 1146, 1112, 1058, 939, 895, 818, 738, 596, 532 cm^–1^; ^1^H NMR (600 MHz, DMSO-*d*_6_) *δ* 11.98 (s, 1H), 8.52 (s, 1H), 8.31 (d, 1H, *J* = 5.18 Hz), 8.27 (d, 1H, *J* = 7.85 Hz), 8.13 (d, 1H, *J* = 5.14 Hz), 7.70–7.65 (m, 3H), 7.60 (t, 1H, *J* = 7.60 Hz), 7.46 (d, 1H, *J* = 8.83 Hz), 7.29 (t, 1H, *J* = 7.45 Hz), 6.25 (s, 1H), 6.15 (s, 2H), 5.44 (s, 3H); ^13^C NMR (151 MHz, DMSO-*d*_6_) *δ* 163.19, 161.09, 151.41, 140.74, 140.71, 137.93, 137.87, 133.81, 132.51, 128.74, 128.58, 128.27, 126.33, 121.93, 120.74, 119.67, 118.67, 116.56, 114.88, 112.07, 92.24, 63.06, 51.32; ESI-MS: *m/z* calculated for C_24_H_16_ClN_5_O_3_: 457.1, found 458.0 (M + 1)^+^; Anal. Calcd. for C_24_H_16_ClN_5_O_3_: C, 62.96; H, 3.52; N, 15.30. Found: C, 63.12; H, 3.54; N, 15.34.

(4) 4-((1-((9*H*-Pyrido[3,4-*b*] indol-1-yl) methyl)-1*H*-1,2,3-triazol-4-yl) methoxy)-6-fluoro-2*H*-chromen-2-one (**4d**).

From the reaction of **1d** (0.035 g) and after purification by column chromatography (mobile phase dichloromethane/methanol 9.5:0.5) and recrystallization from ethanol, 0.040 g (56%) of a white solid **4d** was obtained; reaction time 2 h; mp 239.5–241.0 °C (decomp.); IR (ATR): *ν*_max_ 3334, 3059, 1732, 1718, 1626, 1572, 1492, 1453, 1432, 1371, 1323, 1255, 1215, 1183, 1130, 1082, 1051, 977, 950, 937, 878, 827, 796, 746, 723, 622, 542 cm^–1^; ^1^H NMR (600 MHz, DMSO-*d*_6_) *δ* 11.98 (s, 1H), 8.53 (s, 1H), 8.30 (d, 1H, *J* = 5.18 Hz), 8.27 (d, 1H, *J* = 7.84 Hz), 8.13 (d, 1H, *J* = 5.13 Hz), 7.68 (d, 1H, *J* = 8.20 Hz), 7.61–7.59 (ddd, 1H, *J* = 1.23, 7.03, 8.23 Hz), 7.56–7.52 (td, 1H, *J* = 3.07, 8.60 Hz), 7.49–7.44 (m, 2H), 7.29 (t, 1H, *J* = 7.40 Hz), 6.25 (s, 1H), 6.15 (s, 2H), 5.45 (s, 3H); ^13^C NMR (151 MHz, DMSO-*d*_6_) *δ* 163.51, 161.32, 157.99 (d, *J* = 222.56 Hz), 149.11, 140.80, 140.70, 137.93, 137.86, 133.80, 128.73, 128.57, 126.25, 121.92, 120.73, 120.30 (d, *J* = 20.69 Hz), 119.66, 118.68 (d, *J* = 7.88 Hz), 116.17 (d, *J* = 11.83 Hz), 114.87, 112.06, 108.42 (d, *J* = 29.57 Hz), 92.17, 63.05, 51.29; ESI-MS: *m/z* calculated for C_24_H_16_FN_5_O_3_: 441.1, found 442.1 (M + 1)^+^; Anal. Calcd. for C_24_H_16_FN_5_O_3_: C, 65.30; H, 3.65; N, 15.87. Found: C, 65.40; H, 3.64; N, 15.88.

#### 3.1.4. General Procedure for the Synthesis of Harmirins **5a–d**

To a solution of compound **3** (0.042 g, 0.176 mmol) and the corresponding *O*-alkylated coumarin **1a**–**d** (0.160 mmol) in methanol (3.5 mL), Cu(OAc)_2_ (0.01 mmol) was added. The reaction mixture was stirred overnight at r.t. The solvent was removed under reduced pressure. The crude product was purified by column chromatography.

(1) 4-((1-((1-Methyl-9*H*-pyrido[3,4-*b*]indol-3-yl)methyl)-1*H*-1,2,3-triazol-4-yl)methoxy)-2*H*-chromen-2-one (**5a**).

From the reaction of **1a** (0.032 g) and after purification by column chromatography (mobile phase dichloromethane/methanol 9.5:0.5) and trituration with diethyl ether, 0.045 g (64%) of a white solid **5a** was obtained; mp 149.5–153.5 °C (decomp.); IR (ATR): *ν*_max_ 3292, 1686, 1621, 1566, 1493, 1455, 1357, 1274, 1234, 1191, 1141, 1109, 1051, 937, 881, 812, 767, 731, 647, 588 cm^–1^; ^1^H NMR (400 MHz, DMSO-*d*_6_) *δ* 11.67 (s, 1H), 8.46 (s, 1H), 8.17 (d, 1H, *J* = 7.85 Hz), 8.00 (s, 1H), 7.73 (dd, 1H, *J* = 1.30, 7.90 Hz), 7.67–7.59 (m, 2H), 7.54 (t, 1H, *J* = 7.60 Hz), 7.40 (d, 1H, *J* = 8.23 Hz), 7.32 (t, 1H, *J* = 7.56 Hz), 7.24 (t, 1H, *J* = 7.38 Hz), 6.17 (s, 1H), 5.81 (s, 2H), 5.43 (s, 2H), 2.75 (s, 3H); ^13^C NMR (101 MHz, DMSO-*d*_6_) *δ* 164.34, 161.53, 152.76, 142.43, 142.18, 140.96, 140.77, 134.00, 132.79, 128.10, 127.60, 125.64, 124.20, 122.87, 121.69, 120.91, 119.46, 116.45, 115.08, 112.09, 112.00, 91.32, 62.78, 55.28, 20.38; ESI-MS: *m/z* calculated for C_25_H_19_N_5_O_3_: 437.2, found 438.4 (M + 1)^+^; Anal. Calcd. for C_25_H_19_N_5_O_3_: C, 68.64; H, 4.38; N, 16.01. Found: C, 68.80; H, 4.38; N, 16.03.

(2) 6-Methyl-4-((1-((1-methyl-9*H*-pyrido[3,4-*b*] indol-3-yl) methyl)-1*H*-1,2,3-triazol-4-yl) methoxy)-2*H*-chromen-2-one (**5b**).

From the reaction of **1b** (0.034 g) and after purification by column chromatography (mobile phase dichloromethane/methanol 9.5:0.5) and trituration with diethyl ether, 0.033 g (45%) of a white solid **5b** was obtained; mp 263.0–267.5 °C (decomp.); IR (ATR): *ν*_max_ 3149, 3092, 1721, 1629, 1576, 1499, 1431, 1384, 1355, 1323, 1281, 1250, 1208, 1132, 1102, 1059, 936, 902, 857, 821, 795, 737, 584, 533 cm^–1^; ^1^H NMR (400 MHz, DMSO-*d*_6_) *δ* 11.67 (s, 1H), 8.45 (s, 1H), 8.13 (d, 1H, *J* = 7.87 Hz), 8.00 (s, 1H), 7.61–7.52 (m, 2H), 7.50 (br. s, 1H), 7.45 (dd, 1H, *J* = 1.81, 8.46 Hz), 7.29 (d, 1H, *J* = 8.41 Hz), 7.26–7.22 (m, 1H), 6.14 (s, 1H), 5.81 (s, 2H), 5.41 (s, 2H), 2.75 (s, 3H), 2.33 (s, 3H); ^13^C NMR (101 MHz, DMSO-*d*_6_) *δ* 164.34, 161.68, 150.92, 142.47, 142.18, 140.94, 140.77, 134.00, 133.61, 133.55, 128.10, 127.60, 125.71, 122.29, 121.68, 120.91, 119.45, 116.26, 114.76, 120.09, 111.98, 91.25, 62.70, 55.27, 20.38, 20.26; ESI-MS: *m/z* calculated for C_26_H_21_N_5_O_3_: 451.2, found 452.1 (M + 1)^+^; Anal. Calcd. for C_26_H_21_N_5_O_3_: C, 69.17; H, 4.69; N, 15.51. Found: C, 69.29; H, 4.70; N, 15.54.

(3) 6-Chloro-4-((1-((1-methyl-9*H*-pyrido[3,4-*b*]indol-3-yl)methyl)-1*H*-1,2,3-triazol-4-yl) methoxy)-2*H*-chromen-2-one (**5c**).

From the reaction of **1c** (0.038 g) and after purification by column chromatography (mobile phase dichloromethane/methanol 9.5:0.5) and trituration with diethyl ether, 0.045 g (59%) of an off-white solid **5c** was obtained; mp 254.0–257.5 °C (decomp.); IR (ATR): *ν*_max_ 3259, 3149, 3091, 1720, 1623, 1563, 1500, 1427, 1355, 1310, 1245, 1186, 1149, 1117, 1058, 931, 857, 825, 795, 735, 705, 677, 584, 532 cm^–1^; ^1^H NMR (600 MHz, DMSO-*d*_6_) *δ* 11.67 (s, 1H), 8.48 (s, 1H), 8.17 (d, 1H, *J* = 7.88 Hz), 7.99 (s, 1H), 7.69 (dd, 1H, *J* = 2.63, 8.84 Hz), 7.66 (d, 1H, *J* = 2.53 Hz), 7.60 (d, 1H, *J* = 8.18 Hz), 7.55–7.53 (ddd, 1H, *J* = 1.19, 6.97, 8.21 Hz), 7.45 (d, 1H, *J* = 8.87 Hz), 7.25–7.22 (ddd, 1H, *J* = 1.05, 6.98, 7.98 Hz), 6.24 (s, 1H), 5.81 (s, 2H), 5.44 (s, 2H), 2.75 (s, 3H); ^13^C NMR (151 MHz, DMSO-*d*_6_) *δ* 163.17, 161.09, 151.41, 142.48, 142.17, 140.84, 140.78, 134.00, 132.49, 128.26, 128.09, 127.60, 125.73, 121.94, 121.69, 120.91, 119.45, 118.65, 116.57, 112.09, 111.93, 92.22, 63.10, 55.28, 20.39; ESI-MS: *m/z* calculated for C_25_H_18_ClN_5_O_3_: 471.1, found 472.0 (M + 1)^+^; Anal. Calcd. for C_25_H_18_ClN_5_O_3_: C, 63.63; H, 3.84; N, 14.84. Found: C, 63.53; H, 3.85; N, 15.56.

(4) 6-Fluoro-4-((1-((1-methyl-9*H*-pyrido[3,4-*b*]indol-3-yl)methyl)-1*H*-1,2,3-triazol-4-yl)methoxy)-2*H*-chromen-2-one (**5d**).

From the reaction of **1d** (0.035 g) and after purification by column chromatography (mobile phase dichloromethane/methanol 9.5:0.5) and trituration with diethyl ether, 0.027 g (37%) of a white solid **5d** was obtained; mp 259.0–261.5 °C (decomp.); IR (ATR): *ν*_max_ 3151, 3095, 1715, 1630, 1575, 1499, 1453, 1366, 1320, 1254, 1216, 1176, 1131, 1081, 1055, 968, 922, 877, 827, 737, 704, 678, 588, 563 cm^–1^; ^1^H NMR (600 MHz, DMSO-*d*_6_) *δ* 11.67 (s, 1H), 8.48 (s, 1H), 8.17 (d, 1H, *J* = 8.27 Hz), 7.99 (s, 1H), 7.60 (d, 1H, *J* = 8.18 Hz), 7.56–7.51 (m, 2H), 7.48–7.45 (m, 2H), 7.25–7.22 (ddd, 1H, *J* = 1.04, 6.99, 8.02 Hz), 6.24 (s, 1H), 5.81 (s, 2H), 5.44 (s, 2H), 2.75 (s, 3H); ^13^C NMR (151 MHz, DMSO-*d*_6_) *δ* 163.50, 161.32, 157.99 (d, *J* = 236.63 Hz), 149.10, 142.49, 142.17, 140.91, 140.78, 134.00, 128.10, 127.60, 125.65, 121.69, 120.91, 120.17 (d, *J* = 26.32 Hz), 119.45, 118.67 (d, *J* = 9.06 Hz), 116.18 (d, *J* = 8.15 Hz), 112.10, 111.94, 108.44 (d, *J* = 24.70 Hz), 92.16, 63.09, 55.28, 20.38; ESI-MS: *m/z* calculated for C_25_H_18_FN_5_O_3_: 455.1, found 456.1 (M + 1)^+^; Anal. Calcd. for C_25_H_18_FN_5_O_3_: C, 65.93; H, 3.98; N, 15.38. Found: C, 65.87; H, 3.98; N, 15.39.

#### 3.1.5. General Procedure for the Synthesis of Harmirins **11a–d**

Method A: To a solution of compound **8** (0.038 g, 0.16 mmol) and the corresponding 4-azidocoumarin **7a**–**d** (0.176 mmol) in methanol (3.5 mL), Cu(OAc)_2_ (0.01 mmol) was added. The reaction mixture was stirred at r.t for 48 h. The solvent was removed under reduced pressure. The crude product was purified by column chromatography.

Method B: A mixture of compound **8** (0.038 g, 0.16 mmol), corresponding 4-azidocoumarin **7a**–**d** (0.176 mmol) and Cu(OAc)_2_ (0.01 mmol) in methanol (1.5 mL) was heated at 70 °C in microwave reactor for 25 min (*P* = 75 W). The solvent was removed under reduced pressure. The crude product was purified by column chromatography.

Method C: To a solution of compound **8** (0.038 g, 0.16 mmol) and the corresponding 4-azidocoumarin **7a**–**d** (0.176 mmol) in methanol (3.5 mL), Cu(OAc)_2_ (0.02 mmol) was added. The reaction mixture was stirred at 50 °C for four days. The solvent was removed under reduced pressure. The crude product was purified by column chromatography.

(1) 4-(4-(((1-Methyl-9*H*-pyrido[3,4-*b*]indol-6-yl)oxy)methyl)-1*H*-1,2,3-triazol-1-yl)-2*H*-chromen-2-one (**11a**).

Method A, from the reaction of **7a** (0.033 g) and after purification by column chromatography (mobile phase dichloromethane/methanol 9:1) and trituration with diethyl ether, 0.043 g (63%) of a yellow solid **11a** was obtained; mp 235.5–238.0 °C (decomp.); IR (ATR): *ν*_max_ 3349, 3035, 1726, 1606, 1572, 1499, 1443, 1391, 1360, 1291, 1256, 1202, 1133, 1106, 1037, 1003, 944, 862, 808, 765, 701, 650, 616, 524 cm^–1^; ^1^H NMR (400 MHz, DMSO-*d*_6_) *δ* 11.43 (s, 1H), 8.99 (s, 1H), 8.19 (d, 1H, *J* = 5.33 Hz), 7.98 (s, 1H), 7.93 (d, 1H, *J* = 5.32 Hz), 7.84–7.76 (m, 2H), 7.60–7.53 (m, 2H), 7.42 (t, 1H, *J* = 7.69 Hz), 7.30 (dd, 1H, *J* = 2.45, 8.85 Hz), 7.00 (s, 1H), 5.40 (s, 2H), 2.75 (s, 3H); ^13^C NMR (101 MHz, DMSO-*d*_6_) *δ* 159.47, 153.66, 151.87, 145.91, 143.89, 142.23, 136.91, 135.60, 135.10, 133.47, 126.75, 126.37, 125.47, 124.98, 121.37, 118.46, 117.19, 114.36, 112.85, 110.66, 105.33, 61.62, 20.36; ESI-MS: *m/z* calculated for C_24_H_17_N_5_O_3_: 423.1, found 424.1 (M + 1)^+^; Anal. Calcd. for C_24_H_17_N_5_O_3_: C, 68.08; H, 4.05; N, 16.54. Found: C, 67.88; H, 4.04; N, 16.48.

(2) 6-Methyl-4-(4-(((1-methyl-9*H*-pyrido[3,4-*b*]indol-6-yl)oxy)methyl)-1*H*-1,2,3-triazol-1-yl)-2*H*-chromen-2-one (**11b**).

Method B, from the reaction of **7b** (0.035 g) and after purification by column chromatography (mobile phase dichloromethane/methanol 9.5:0.5) and recrystallization from ethanol, 0.018 g (26%) of a yellow solid **11a** was obtained; mp 235.0–242.0 °C (decomp.); IR (ATR): *ν*_max_ 3340, 3036, 1725, 1623, 1572, 1499, 1465, 1405, 1378, 1282, 1256, 1199, 1132, 1105, 1040, 1009, 945, 864, 807, 701, 610, 528 cm^–1^; ^1^H NMR (400 MHz, DMSO-*d*_6_) *δ* 11.42 (s, 1H), 8.97 (s, 1H), 8.18 (d, 1H, *J* = 5.28 Hz), 7.98 (s, 1H), 7.93 (d, 1H, *J* = 5.31 Hz), 7.60–7.48 (m, 4H), 7.30 (dd, 1H, *J* = 2.45, 8.84 Hz), 6.95 (s, 1H), 5.40 (s, 1H), 2.75 (s, 3H), 2.34 (s, 3H); ^13^C NMR (101 MHz, DMSO-*d*_6_) *δ* 159.57, 151.85, 145.91, 143.87, 142.23, 136.94, 135.58, 135.11, 134.41, 134.35, 126.74, 126.38, 124.80, 121.38, 118.42, 117.00, 114.08, 112.85, 112.72, 110.71, 105.29, 61.60, 20.41, 20.37; ESI-MS: *m/z* calculated for C_25_H_19_N_5_O_3_: 437.2, found 438.1 (M + 1)^+^; Anal. Calcd. for C_25_H_19_N_5_O_3_: C, 68.64; H, 4.38; N, 16.01. Found: C, 68.90; H, 4.40; N, 16.07.

(3) 6-Chloro-4-(4-(((1-methyl-9*H*-pyrido[3,4-*b*]indol-6-yl)oxy)methyl)-1*H*-1,2,3-triazol-1-yl)-2*H*-chromen-2-one (**11c**).

Method C, from the reaction of **7c** (0.039 g) and after purification by column chromatography (mobile phase cyclohexane/ethyl acetate/methanol 3:1:0.75) and trituration with diethyl ether, 0.019 g (26%) of a yellow solid **11c** was obtained; mp 220.0–225.0 °C (decomp.); IR (ATR): *ν*_max_ 3245, 3165, 1731, 1620, 1558, 1500, 1479, 1452, 1401, 1344, 1284, 1261, 1230, 1213, 1185, 1111, 1052, 1013, 947, 827, 810, 682, 619, 558 cm^–1^; ^1^H NMR (400 MHz, DMSO-*d*_6_) *δ* 11.43 (s, 1H), 9.01 (s, 1H), 8.18 (d, 1H, *J* = 5.33 Hz), 7.97 (s, 1H), 7.92–7.91 (m, 2H), 7.83 (d, 1H, *J* = 8.90 Hz), 7.64 (d, 1H, *J* = 8.89 Hz), 7.55 (d, 1H, *J* = 8.77 Hz), 7.29 (d, 1H, *J* = 8.83 Hz), 7.09 (s, 1H), 5.40 (s, 2H), 2.74 (s, 3H); ^13^C NMR (101 MHz, DMSO-*d*_6_) *δ* 159.05, 152.34, 151.81, 144.58, 144.04, 142.22, 136.96, 135.55, 135.08, 133.00, 128.78, 126.67, 126.24, 124.78, 121.36, 119.23, 118.32, 115.61, 112.81, 112.67, 111.26, 105.27, 61.59, 20.36; ESI-MS: *m/z* calculated for C_24_H_16_ClN_5_O_3_: 457.1, found 458.0 (M + 1)^+^; Anal. Calcd. for C_24_H_16_ClN_5_O_3_: C, 62.96; H, 3.52; N, 15.30. Found: C, 63.15; H, 3.53; N, 15.36.

(4) 6-Fluoro-4-(4-(((1-methyl-9*H*-pyrido[3,4-*b*]indol-6-yl)oxy)methyl)-1*H*-1,2,3-triazol-1-yl)-2*H*-chromen-2-one (**11d**).

Method B, from the reaction of **7d** (0.036 g) and after purification by column chromatography (mobile phase dichloromethane/methanol 9.5:0.5) and recrystallization from ethanol, 0.024 g (34%) of a yellow solid **11d** was obtained; mp 224.5–225.5 °C (decomp.); IR (ATR): *ν*_max_ 3357, 3028, 1730, 1527, 1512, 1488, 1464, 1416, 1353, 1291, 1261, 1238, 1202, 1159, 1074, 1035, 989, 942, 871, 808, 719, 700, 610, 523 cm^–1^; ^1^H NMR (600 MHz, DMSO-*d*_6_) *δ* 11.41 (s, 1H), 9.02 (s, 1H), 8.18 (d, 1H, *J* = 5.29 Hz), 7.97 (s, 1H), 7.92 (d, 1H, *J* = 5.26 Hz), 7.73–7.66 (m, 3H), 7.54 (d, 1H, *J* = 8.80 Hz), 7.29 (dd, 1H, *J* = 2.53, 8.82 Hz), 7.09 (s, 1H), 5.40 (s, 2H), 2.74 (s, 3H); ^13^C NMR (151 MHz, DMSO-*d*_6_) *δ* 159.30, 158.05 (d, *J* = 241.22 Hz), 151.86. 150.11, 144.93 (d, *J* = 2.52 Hz), 144.05, 142.25, 137.00, 135.56, 135.11, 126.69, 126.23, 121.39, 120.82 (d, *J* = 25.77 Hz), 119.33 (d, *J* = 8.37 Hz), 118.35, 115.20 (d, *J* = 7.36 Hz), 112.83, 111.44 (d, *J* = 25.53 Hz), 111.16, 105.26, 61.62, 20.40; ESI-MS: *m/z* calculated for C_24_H_16_FN_5_O_3_: 441.1, found 442.1 (M + 1)^+^; Anal. Calcd. for C_24_H_16_FN_5_O_3_: C, 65.30; H, 3.65; N, 15.87. Found: C, 65.05; H, 3.52; N, 15.81.

#### 3.1.6. General Procedure for the Synthesis of Harmirins **12a–d**

To a solution of compound **9** (0.038 g, 0.16 mmol) and the corresponding 4-azidocoumarin **7a**–**d** (0.176 mmol) in methanol (3.5 mL), Cu(OAc)_2_ (0.01 mmol) was added. The reaction mixture was stirred at r.t overnight (**12a**,**b**), for two (**12c**) or five (**12d**) days. The solvent was removed under reduced pressure. The crude product was purified by column chromatography.

(1) 4-(4-(((1-Methyl-9*H*-pyrido[3,4-*b*]indol-7-yl)oxy)methyl)-1*H*-1,2,3-triazol-1-yl)-2*H*-chromen-2-one (**12a**).

From the reaction of **7a** (0.033 g) and after purification by column chromatography (mobile phase dichloromethane/methanol 9:1) and trituration with diethyl ether, 0.036 g (53%) of a pale yellow solid **12a** was obtained; mp 223.5–225.5 °C (decomp.); IR (ATR): *ν*_max_ 3287, 3177, 3136, 3090, 3025, 2864, 1697, 1608, 1558, 1484, 1439, 1382, 1351, 1275, 1233, 1177, 1138, 1106, 1056, 1003, 964, 872, 820, 738, 651, 615, 572, 488 cm^–1^; ^1^H NMR (600 MHz, DMSO-*d*_6_) *δ* 11.52 (s, 1H), 8.99 (s, 1H), 8.17 (d, 1H, *J* = 5.27 Hz), 8.11 (d, 1H, *J* = 8.62 Hz), 7.85–7.82 (m, 2H), 7.79 (t, 1H, *J* = 8.00 Hz), 7.60 (d, 1H, *J* = 8.34 Hz), 7.45 (t, 1H, *J* = 7.66 Hz), 7.25 (s, 1H), 7.00–6.97 (m, 2H), 5.44 (s, 2H), 2.74 (s, 3H); ^13^C NMR (151 MHz, DMSO-*d*_6_) *δ* 159.45, 158.71, 153.65, 145.90, 143.64, 141.84, 141.30, 137.57, 134.61, 133.48, 127.23, 126.51, 125.45, 125.00, 122.77, 117.19, 115.31, 114.36, 112.04, 110.72, 109.46, 96.02, 61.14, 20.23; ESI-MS: *m/z* calculated for C_24_H_17_N_5_O_3_: 423.1, found 424.1 (M + 1)^+^; Anal. Calcd. for C_24_H_17_N_5_O_3_: C, 68.08; H, 4.05; N, 16.54. Found: C, 68.35; H, 4.06; N, 16.60.

(2) 6-Methyl-4-(4-(((1-methyl-9*H*-pyrido[3,4-*b*]indol-7-yl)oxy)methyl)-1*H*-1,2,3-triazol-1-yl)-2*H*-chromen-2-one (**12b**).

From the reaction of **7b** (0.035 g) and after purification by column chromatography (mobile phase dichloromethane/methanol 9:1) and trituration with diethyl ether, 0.040 g (57%) of a white solid **12b** was obtained; mp 225.5–229.5 °C (decomp.); IR (ATR): *ν*_max_ 3286, 3149, 3113, 3067, 3013, 2926, 2861, 1723, 1630, 1570, 1483, 1452, 1357, 1323, 1276, 1233, 1181, 1137, 1105, 1048, 1012, 946, 890, 801, 733, 610, 568, 519 cm^–1^; ^1^H NMR (400 MHz, DMSO-*d*_6_) *δ* 11.51 (s, 1H), 8.97 (s, 1H), 8.16 (s, 1H), 8.11 (d, 1H, *J* = 8.52 Hz), 7.84 (d, 1H, *J* = 4.60 Hz), 7.60–7.48 (m, 3H), 7.24 (s, 1H, 12), 6.99–6.96 (m, 2H), 5.45 (s, 2H), 2.74 (s, 3H), 2.35 (s, 3H); ^13^C NMR (101 MHz, DMSO-*d*_6_) *δ* 159.56, 158.68, 151.85, 145.90, 143.61, 141.83, 141.31, 137.61, 134.61, 134.42, 134.36, 127.21, 126.54, 124.77, 122.78, 117.01, 115.32, 114.08, 112.04, 110.78, 96.02, 61.14, 20.43, 20.25; ESI-MS: *m/z* calculated for C_25_H_19_N_5_O_3_: 437.2, found 438.1 (M + 1)^+^; Anal. Calcd. for C_25_H_19_N_5_O_3_: C, 68.64; H, 4.38; N, 16.01. Found: C, 68.37; H, 4.37; N, 15.95.

(3) 6-Chloro-4-(4-(((1-methyl-9*H*-pyrido[3,4-*b*]indol-7-yl)oxy)methyl)-1*H*-1,2,3-triazol-1-yl)-2*H*-chromen-2-one (**12c**).

From the reaction of **7c** (0.039 g) and after purification by column chromatography (mobile phase dichloromethane/methanol 9:1) and trituration with diethyl ether, 0.029 g (40%) of a pale yellow solid **12c** was obtained; mp 244.0–247.0 °C (decomp.); IR (ATR): *ν*_max_ 3149, 3090, 3030, 2991, 1619, 1601, 1569, 1484, 1447, 1369, 1322, 1217, 1137, 1097, 1058, 1021, 910, 859, 805, 734, 669, 590, 540, 481 cm^–1^; ^1^H NMR (400 MHz, DMSO-*d*_6_) *δ* 11.51 (s, 1H), 9.01 (s, 1H), 8.17 (d, 1H, *J* = 5.30 Hz), 8.11 (d, 1H, *J* = 8.65 Hz), 7.91 (s, 1H), 7.85–7.82 (m, 2H), 7.64 (d, 1H, *J* = 8.88 Hz), 7.24 (s, 1H), 7.10 (s, 1H), 6.98 (dd, 1H, *J* = 2.13, 8.65 Hz), 5.45 (s, 2H), 2.74 (s, 3H); ^13^C NMR (101 MHz, DMSO-*d*_6_) *δ* 159.07, 158.68, 152.37, 144.60, 143.79, 141.83, 141.30, 137.59, 134.61, 133.05, 128.82, 127.22, 126.44, 124.78, 122.78, 119.28, 115.65, 115.33, 112.04, 111.38, 109.41, 96.02, 61.13, 20.24; ESI-MS: *m/z* calculated for C_24_H_16_ClN_5_O_3_: 457.1, found 458.0 (M + 1)^+^; Anal. Calcd. for C_24_H_16_ClN_5_O_3_: C, 62.96; H, 3.52; N, 15.30. Found: C, 62.71; H, 3.51; N, 15.24.

(4) 6-Fluoro-4-(4-(((1-methyl-9*H*-pyrido[3,4-*b*]indol-7-yl)oxy)methyl)-1*H*-1,2,3-triazol-1-yl)-2*H*-chromen-2-one (**12d**).

From the reaction of **7d** (0.036 g) and after purification by column chromatography (mobile phase dichloromethane/methanol 9.5:0.5) and trituration with diethyl ether, 0.028 g (39%) of a pale yellow solid **12d** was obtained; mp 249.0–251.0 °C (decomp.); IR (ATR): *ν*_max_ 3275, 3175, 3141, 3095, 3067, 3012, 1718, 1626, 1566, 1483, 1456, 1415, 1382, 1345, 1275, 1231, 1169, 1103, 1056, 1006, 966, 869, 806, 735, 614, 569, 528, 491 cm^–1^; ^1^H NMR (600 MHz, DMSO-*d*_6_) *δ* 11.48 (s, 1H), 9.02 (s, 1H), 8.16 (d, 1H, *J* = 5.24 Hz), 8.10 (d, 1H, *J* = 8.61 Hz), 7.83 (d, 1H, *J* = 5.28 Hz), 7.72–7.67 (m, 3H), 7.24 (s, 1H), 7.09 (s, 1H), 6.98 (dd, 1H, *J* = 2.27, 8.61 Hz), 5.44 (s, 2H), 2.73 (s, 3H); ^13^C NMR (151 MHz, DMSO-*d*_6_) *δ* 159.26, 158.55 (d, *J* = 246.21 Hz), 158.64, 150.10, 144.92 (d, *J* = 2.92 Hz), 143.78, 141.77, 141.36, 137.72, 134.62, 127.12, 126.38, 122.73, 120.82 (d, *J* = 31.71 Hz), 119.33 (d, *J* = 10.57 Hz), 115.34, 115.20 (d, *J* = 10.57 Hz), 111.99, 111.39 (d, *J* = 31.70 Hz), 111.27, 111.23, 96.01, 61.14, 20.30; ESI-MS: *m/z* calculated for C_24_H_16_FN_5_O_3_: 441.1, found 442.1 (M + 1)^+^; Anal. Calcd. for C_24_H_16_FN_5_O_3_: C, 65.30; H, 3.65; N, 15.87. Found: C, 65.45; H, 3.65; N, 15.90.

#### 3.1.7. General Procedure for the Synthesis of Harmirins **13a–d**

Method A: To a solution of compound **10** (0.040 g, 0.160 mmol) and the corresponding 4-azidocoumarin **7a**–**d** (0.176 mmol) in methanol (3.5 mL), Cu(OAc)_2_ (0.01 mmol) was added. The reaction mixture was stirred overnight at r.t. The solvent was removed under reduced pressure. The crude product was purified by column chromatography.

Method B: A mixture of compound **10** (0.040 g, 0.160 mmol), corresponding 4-azidocoumarin **7d** (0.176 mmol) and Cu(OAc)_2_ (0.01 mmol) in methanol (1.5 mL) was heated at 70 °C in microwave reactor for 40 min (*P* = 75 W). The solvent was removed under reduced pressure. The crude product was purified by column chromatography.

(1) 4-(4-((7-Methoxy-1-methyl-9*H*-pyrido[3,4-*b*]indol-9-yl)methyl)-1*H*-1,2,3-triazol-1-yl)-2*H*-chromen-2-one (**13a**).

Method A, from the reaction of **7a** (0.033 g) and after purification by column chromatography (mobile phase dichloromethane/methanol 9.5:0.5) and trituration with diethyl ether, 0.030 g (43%) of an off-white solid **13a** was obtained; mp 239.0–243.5 °C (decomp.); IR (ATR): *ν*_max_ 3144, 3086, 3062, 2837, 1760, 1717, 1622, 1564, 1494, 1439, 1406, 1349, 1237, 1167, 1104, 1041, 948, 870, 813, 768, 646 cm^–1^; ^1^H NMR (400 MHz, DMSO-*d*_6_) *δ* 8.78 (s, 1H), 8.21 (br. s, 1H), 8.11 (d, 1H, *J* = 8.60 Hz), 7.91 (d, 1H, *J* = 4.71 Hz), 7.76–7.72 (m, 2H), 7.55 (dd, 1H, *J* = 0.95, 8.70 Hz), 7.41–7.37 (m, 2H), 6.92-6.89 (m, 2H), 6.03 (s, 2H), 3.92 (s, 3H), 3.10 (s, 3H); ^13^C NMR (101 MHz, DMSO-*d*_6_) *δ* 160.62, 159.41), 153.57, 145.69, 144.74, 142.72, 141.18, 138.13, 134.87, 133.37, 128.63, 125.40, 124.90, 122.40, 117.13, 114.52, 114.36, 112.34, 110.71, 109.47, 94.16, 55.66, 23.30; ESI-MS: *m/z* calculated for C_25_H_19_N_5_O_3_: 437.2, found 438.1 (M + 1)^+^; Anal. Calcd. for C_25_H_19_N_5_O_3_: C, 68.64; H, 4.38; N, 16.01. Found: C, 68.57; H, 4.37; N, 15.98.

(2) 4-(4-((7-Methoxy-1-methyl-9*H*-pyrido[3,4-*b*]indol-9-yl)methyl)-1*H*-1,2,3-triazol-1-yl)-6-methyl-2*H*-chromen-2-one (**13b**).

Method A, from the reaction of **7b** (0.035 g) and after purification by column chromatography (mobile phase dichloromethane/methanol 9.5:0.5) and trituration with diethyl ether, 0.038 g (53%) of a white solid **13b** was obtained; mp 253.0–255.0 °C (decomp.); IR (ATR): *ν*_max_ 3145, 3096, 3058, 3000, 1728, 1622, 1568, 1496, 1445, 1408, 1367, 1336, 1284, 1243, 1195, 1165, 1137, 1039, 1010, 945, 889, 813, 748, 720, 649, 610, 550 cm^–1^; ^1^H NMR (400 MHz, DMSO-*d*_6_) *δ* 8.78 (s, 1H), 8.21 (d, 1H, *J* = 5.16 Hz), 8.11 (d, 1H, *J* = 8.60 Hz), 7.90 (d, 1H, *J* = 5.17 Hz), 7.55 (dd, 1H, *J* = 1.48, 8.55 Hz), 7.45–7.40 (m, 3H), 6.91 (dd, 1H, *J* = 2.02, 8.60 Hz), 6.86 (s, 1H), 6.03 (s, 2H), 3.91 (s, 3H), 3.11 (s, 3H), 2.31 (s, 3H); ^13^C NMR (101 MHz, DMSO-*d*_6_) *δ* 160.63, 159.51, 151.77, 145.67, 144.69, 142.70, 141.16, 138.18, 134.77, 134.30, 134.24, 128.64, 124.95, 124.64, 122.41, 116.96, 114.53, 114.06, 112.30, 110.72, 109.45, 94.16, 55.66, 54.91, 23.30, 20.38; ESI-MS: *m/z* calculated for C_26_H_21_N_5_O_3_: 451.2, found 452.1 (M + 1)^+^; Anal. Calcd. for C_26_H_21_N_5_O_3_: C, 69.17; H, 4.69; N, 15.51. Found: C, 69.31; H, 4.69; N, 15.54.

(3) 6-Chloro-4-(4-((7-methoxy-1-methyl-9*H*-pyrido[3,4-*b*]indol-9-yl)methyl)-1*H*-1,2,3-triazol-1-yl)-2*H*-chromen-2-one (**13c**).

Method A, from the reaction of **7c** (0.039 g) and after purification by column chromatography (mobile phase dichloromethane/methanol 9.5:0.5) and trituration with diethyl ether, 0.033 g (44%) of an off-white solid **13c** was obtained; mp 233.0–239.5 °C (decomp.); IR (ATR): *ν*_max_ 3136, 3080, 2997, 2961, 1729, 1624, 1562, 1445, 1410, 1344, 1256, 1221, 1169, 1120, 1041, 971, 930, 816, 720, 682, 644, 597, 556, 512 cm^–1^; ^1^H NMR (400 MHz, DMSO-*d*_6_) *δ* 8.79 (s, 1H), 8.20 (d, 1H, *J* = 5.19 Hz), 8.11 (d, 1H, *J* = 8.60 Hz), 7.90 (d, 1H, *J* = 5.19 Hz), 7.82–7.78 (m, 2H), 7.60 (d, 1H, *J* = 8.68 Hz), 7.39 (s, 1H), 7.01 (s, 1H), 6.90 (dd, 1H, *J* = 2.05, 8.60 Hz), 6.03 (s, 2H), 3.91 (s, 3H), 3.09 (s, 3H); ^13^C NMR (101 MHz, DMSO-*d*_6_) *δ* 160.64, 159.01, 152.27, 144.95, 144.41, 142.72, 141.12, 138.17, 134.71, 132.93, 128.73, 128.65, 124.79, 124.67, 122.39 119.22, 115.65, 114.50, 112.29, 111.36, 109.48, 94.12, 55.67, 23.25; ESI-MS: *m/z* calculated for C_25_H_18_ClN_5_O_3_: 471.1, found 472.0 (M + 1)^+^; Anal. Calcd. for C_25_H_18_ClN_5_O_3_: C, 63.63; H, 3.84; N, 14.84. Found: C, 63.49; H, 3.85; N, 14.87.

(4) 6-Fluoro-4-(4-((7-methoxy-1-methyl-9*H*-pyrido[3,4-*b*]indol-9-yl)methyl)-1*H*-1,2,3-triazol-1-yl)-2*H*-chromen-2-one (**13d**).

Method B, from the reaction of **7d** (0.036 g) and after purification by column chromatography (mobile phase dichloromethane/methanol 9.5:0.5) and recrystallization from ethanol, 0.036 g (49%) of an off-white solid **13d** was obtained; mp 235.0–237.0 °C (decomp.); IR (ATR): *ν*_max_ 3441, 3127, 3072, 2955, 2836, 1728, 1628, 1574, 1490, 1461, 1440, 1406, 1348, 1301, 1256, 1235, 1197, 1163, 1041, 1025, 1008, 945, 887, 827, 728, 714, 611, 523 cm^–1^; ^1^H NMR (600 MHz, DMSO-*d*_6_) *δ* 8.81 (s, 1H), 8.21 (d, 1H, *J* = 5.18 Hz), 8.12 (d, 1H, *J* = 8.60 Hz), 7.92 (d, 1H, *J* = 5.17 Hz), 7.65–7.61 (m, 3H), 7.39 (s, 1H), 7.01 (s, 1H), 6.91 (dd, 1H, *J* = 2.17, 8.56 Hz), 6.03 (s, 2H), 3.91 (s, 3H), 3.09 (s, 3H); ^13^C NMR (151 MHz, DMSO-*d*_6_) *δ* 160.62, 159.24, 158.49 (d, *J* = 235.40 Hz), 150.02, 144.92, 144.75, 142.70, 141.15, 138.18, 134.73, 128.63, 124.77, 122.40, 120.72 (d, *J* = 29.19 Hz), 119.28 (d, *J* = 8.63 Hz), 115.21 (d, *J* = 10.88 Hz), 114.52, 112.30, 111.33 (d, *J* = 24.99 Hz), 111.24, 109.46, 94.14, 55.65, 23.27; ESI-MS: *m/z* calculated for C_25_H_18_FN_5_O_3_: 455.1, found 456.1 (M + 1)^+^; Anal. Calcd. for C_25_H_18_FN_5_O_3_: C, 65.93; H, 3.98; N, 15.38. Found: C, 66.03; H, 3.97; N, 15.39.

### 3.2. Biological Evaluation

#### 3.2.1. Cytotoxicity Assay in Human Cell Lines

The experiments were carried out on five human cell lines purchased from American Type Culture Collection (ATCC, PO BOX 1549, manassas, VA 20108, USA): HepG2 (hepatocellular carcinoma; ATCC^®^ HB-8065™), SW620 (colorectal adenocarcinoma; ATCC^®^ CCL-227™), HCT116 (colorectal carcinoma ATCC^®^ CCL-247™), MCF-7 (breast adenocarcinoma, ATCC^®^ HTB-22™), and HEK293T (embryonic kidney cells; ATCC^®^ CRL-3216™). All cell lines were cultured as monolayers and maintained in Dulbecco’s modified Eagle medium (DMEM) (Capricorn Scientific, USA), supplemented with 10% fetal bovine serum (FBS) (Capricorn Scientific, USA), 100 U/mL penicillin, and 100 µg/mL streptomycin (Capricorn Scientific, USA) in a humidified atmosphere with 5% CO_2_ at 37 °C. Cells were seeded in 96-well plates (Corning, Durham, NC, USA) at 5000–7000 cells per well (depending on the cell doubling time of a specific cell line) in 0.1 mL media and cultured for 24 h. The next day, the medium was aspirated, and cells were treated for 72 h. Only the compounds that led to more than a 50% reduction in mitochondrial metabolic activity at a concentration of 50 µM were selected for further analysis. The following concentrations of selected compounds were used: 25, 10, 5, and 1 µM. Working dilutions were freshly prepared on the day of the testing. A fresh growth medium was added to untreated control cells, which were defined as 100% viable. DMSO (0.13%) in DMEM was considered a negative control. 5-Fluorouracil (5-FU) and harmine were used as positive controls. At the end of treatment, media was removed, and cells were incubated for 1 h with 0.5 mg/mL MTT (Abcam, Cambridge, MA, USA) dissolved in serum-deprived DMEM. The absorbance was directly proportional to cell viability. The MTT-containing media was then removed, and 0.1 mL isopropanol was added per well to lyse cells and dissolve formazan. The optical density was measured at 570 nm using a microplate reader (VICTOR3, PerkinElmer). Each test point was performed in triplicate. The IC_50_ values (concentration required to decrease viability by 50%) were calculated by using nonlinear regression on the sigmoidal dose–response plots and are expressed as mean ± SD.

#### 3.2.2. Cell Localization

The MCF-7 cells were seeded on round microscopic coverslips placed in 24-well-plates (5 × 10^4^ cells per well) and grown at 37 °C and 5% CO_2_ for 24 h in DMEM supplemented with FBS, penicillin, and streptomycin, as described above. Cells were then incubated with compound **12b** (10 µM) for 30 min. Afterward, the medium was discharged, coverslips rinsed twice with PBS, placed on the microscopic slides, and immediately analyzed. The uptake and intracellular distribution of the tested derivative were analyzed under a fluorescence microscope (Olympus BX51) at 400 × magnification, using a DAPI filter. Images were captured with an Olympus DP70 Digital Camera.

#### 3.2.3. Cell Cycle Analysis

MCF-7 cells were seeded onto 6-well plates (3 × 10^5^ cells per well). After 24 h, **12b** was added at 10 µM concentration and harmine at 20 µM concentration. After 24 h or 48 h, the attached cells were trypsinized, combined with floating cells, washed with phosphate buffer saline (PBS), fixed with 70% ethanol, and stored at −20 °C. Immediately before analysis, the cells were washed with PBS and stained with 50 μg/mL of propidium iodide (PI) with the addition of 0.1 μg/μL of RNAse A. The stained cells were then analyzed by BD FACScalibur flow cytometer (20,000 counts were measured). The percentage of cells in each cell cycle phase was determined using FlowJo software (TreeStar Inc., USA). The tests were performed in duplicate and repeated in three separate experiments.

## 4. Conclusions

Twenty novel hybrid compounds comprising two distinct pharmacophores—harmine/β-carboline and coumarin, connected via triazole linker—were synthesized using CuAAC from harmine-based azides and coumarin alkynes (**4** and **5**) and coumarin azides and harmine-based alkynes (**11**–**13**), respectively. The evaluation of their antiproliferative activity in vitro against a panel of human cell lines revealed that seven harmirins display activities in the single-digit micromolar range against MCF-7 and HCT116. Among them, harmirin **12b**, substituted at O-7 of the β-carboline core and bearing the methyl group at position 6 of the coumarin ring, showed the highest selectivity towards cancer cells, in comparison to HEK293T (SIs > 7.2). According to cell localization experiments, harmirin **12b** is localized exclusively in the cytoplasm. Furthermore, cell cycle analysis showed that the treatment of MCF-7 cells with harmirin **12b** induced a strong G1 arrest, accompanied by a drastic reduction in the percentage of cells in the S phase. Taken together, these results might suggest that harmirin **12b** exerts its antiproliferative activity through inhibition of DNA synthesis, rather than DNA damage. Our future work will focus on the elucidation of molecular mechanisms involved in the anticancer activities of harmirins. In summary, our findings indicate that harmirins, harmine–coumarin hybrids, might serve as an important basis for the design and synthesis of new anticancer agents with significant antitumor activity and low toxicity.

## Figures and Tables

**Table 1:** The reaction conditions for the synthesis of harmirins and obtained yields.

Compd.	General Structure	R	Precatalyst	Reducing Agent	Solvent	Temp.	Time	Yield (%)
**4a**	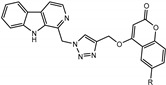	H	CuSO_4_ × 5 H_2_O	Na-ascorbate	*t*-butanol/water	r.t.	1 h	51
**4b**		CH_3_					30 min	45
**4c**		Cl					1 h	69
**4d**		F					2 h	56
**5a**	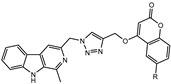	H	Cu(OAc)_2_	methanol	methanol	r.t.	overnight	64
**5b**		CH_3_						45
**5c**		Cl						59
**5d**		F						37
**11a**	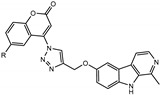	H	Cu(OAc)_2_	methanol	methanol	r.t.	48 h	63
**11b**		CH_3_				70 °C ^a^	25 min	26
**11c**		Cl				50 °C	96 h	26
**11d**		F				70 °C ^a^	25 min	34
**12a**	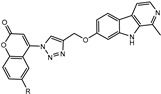	H	Cu(OAc)_2_	methanol	methanol	r.t.	overnight	53
**12b**		CH_3_						57
**12c**		Cl					48 h	40
**12d**		F					120 h	39
**13a**	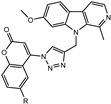	H	Cu(OAc)_2_	methanol	methanol	r.t.	overnight	43
**13b**		CH_3_						53
**13c**		Cl						44
**13d**		F				70 °C ^a^	40 min	49

^a^ microwave-assisted synthesis.

**Table 2:** In vitro cytostatic activity of harmirins **4**, **5**, and **11**–**13** against human cell lines and calculated selectivity indices.

Compd.	IC_50_ ^a^ (µM)					SI ^b^ (HCT116)	SI ^b^ (MCF-7)
	HepG2	SW620	HCT116	MCF-7	Hek293T		
**4a**	29.5 ± 3.5	>50	>50	>50	>50	>1	>1
**4b**	27.3 ± 1.2	>50	>50	35.7 ± 1.0	26.0 ± 1.0	<0.5	0.7
**4c**	>50	>50	>50	39.1 ± 3.6	>50	>1	>1.3
**4d**	>50	>50	33.1 ± 7.7	>50	45.8 ± 0.2	1.4	<0.9
**5a**	17.8 ± 1.8	>50	>50	>50	6.5 ± 1.6	<0.1	<0.1
**5b**	16.8 ± 1.4	>50	3.2 ± 0.5	>50	27.3 ± 2.1	8.6	<0.6
**5c**	18.5 ± 2.3	>50	7.1 ± 0.7	15.0 ± 1.3	8.5 ± 0.3	1.2	0.6
**5d**	>50	>50	>50	>5 0	7.0 ± 1.6	<0.1	<0.1
**11a**	6.4 ± 0.2	8.4 ± 0.3	3.3 ± 0.4	2.2 ± 0.2	6.4 ± 0.2	2	3
**11b**	13.6 ± 0.8	>50	>50	10.4 ± 0.8	21.3 ± 4.1	<0.4	2
**11c**	14.0 ± 1.2	30.3 ± 3.9	15.5 ± 4.4	13.3 ± 1.5	12.2 ± 1.0	0.8	0.9
**11d**	14.2 ± 0.4	12.7 ± 1.8	2.7 ± 0.4	1.9 ± 0.2	6.9 ± 0.6	3.7	3.7
**12a**	19.8 ± 1.1	30.0 ± 3.5	10.0 ± 3.4	21.6 ± 3.7	14.7 ± 0.8	1.5	0.7
**12b**	19.5 ± 1.5	20.8 ± 0.4	4.7 ± 0.6	6.9 ± 0.7	>50	>10.6	>7.2
**12c**	>50	>50	37.6 ± 6.9	38.2 ± 2.5	46.2 ± 4.7	1.2	1.2
**12d**	>50	>50	>50	>50	>50	>1	>1
**13a**	19.6 ± 1.3	>50	3.4 ± 0.6	34.0 ± 2.1	12.4 ± 1.1	3.6	0.4
**13b**	>50	>50	>50	>50	>50	>1	>1
**13c**	>50	>50	34.4 ± 3.3	32.0 ± 1.9	>50	>1.5	>1.6
**13d**	3.1 ± 0.5	3.3 ± 0.4	12.7 ± 0.6	2.7 ± 0.5	3.9 ± 0.3	0.3	1.5
**HAR** ^c^	18.7 ± 0.8	4.7 ± 0.6	4.0 ± 0.8	13.5 ± 1.1	12.6 ± 0.8	3.2	0.9
**5-FU** ^d^	5.5 ± 0.6	9.4 ± 0.3	5.2 ± 2.8	23.9 ± 5.7	8.1 ± 0.8	1.6	0.3

^a^ IC_50_, the concentration that causes 50 % growth inhibition; ^b^ SI, selectivity index; ^c^ HAR, harmine; ^d^ 5-FU; 5-fluorouracil.

## Data Availability

The data presented in this study are available in this article and the Supplementary Materials.

## Supplementary Materials

Tables S1–S6: Analytical data for coumarin **1d**, harmirins **4a**–**d**, **5a**–**d**, **11a**–**d**, **12a**–**d**, **13a**–**d**; Tables S7–S12: ^1^H and ^13^C NMR spectroscopic data for coumarin **1d**, harmirins **4a**–**d**, **5a**–**d**, **11a**–**d**, **12a**–**d**, **13a**–**d**; Table S13: Properties of the harmirins calculated with Chemicalize.org program. The Lipinski and Gelovani parameters.

## Sample Availability

Samples of the compounds are available from the authors.
