# Supplementary material for: Synthesis and Biological Evaluation of Harmirins, Novel Harmine–Coumarin Hybrids as Potential Anticancer Agents
Source: Molecules. 2021 Oct 27;26(21):6490. doi: 10.3390/molecules26216490 (PMC8587047; doi:10.3390/molecules26216490)
Supplement: Supplementary file 1 [file molecules-26-06490-s001.zip › molecules-1425666-supplementary.pdf]

Synthesis and biological evaluation of harmirins, novel harmine-coumarin hybrids as potential anticancer agents

Kristina Pavić [1], Maja Beus [1], Goran Poje [1], Lidija Uzelac [2], Marijeta Kralj [2] and Zrinka Rajić [1,*]

Supplementary Materials

Table of contents

Table S1. Analytical data for *O*-alkylated coumarin **1d**. 3

Table S2. Analytical data for harmirins **4a–d**. 4

Table S3. Analytical data for harmirins **5a–d**. 5

Table S4. Analytical data for harmirins **11a–d**. 6

Table S5. Analytical data for harmirins **12a–d**. 7

Table S6. Analytical data for harmirins **13a–d**. 8

Table S7. ^{1}H and ^{13}C NMR spectroscopic data for *O*-alkylated coumarin **1d**. 9

Table S8. ^{1}H and ^{13}C NMR spectroscopic data for harmirins **4a–d**. 10

Table S9. ^{1}H and ^{13}C NMR spectroscopic data for harmirins **5a–d**. 11

Table S10. ^{1}H and ^{13}C NMR spectroscopic data for harmirins **11a–d**. 12

Table S11. ^{1}H and ^{13}C NMR spectroscopic data for harmirins **12a–d**. 12

Table S12. ^{1}H and ^{13}C NMR spectroscopic data for harmirins **13a–d**. 14

Table S13. Properties of the harmirins calculated with Chemicalize.org program [34]. The Lipinski and Gelovani parameters.[a] 15

Table S1. Analytical data for *O*-alkylated coumarin **1d**.

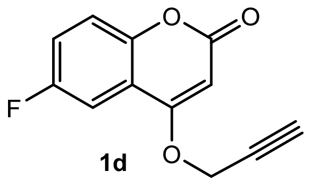

1d

**Compd.**	**Yield (%)**	**Molecular Formula**	M_r	**MS (*m/z*)**	**IR (*υ*/ cm^{-1})**
**1d**	42	$C_{12}H_7FO_3$	218.18	219.0 $(M + 1)^+$, 241.0 $(M + 23)^+$	3274, 3237, 3074, 2135, 1702, 1625, 1575, 1492, 1452, 1392, 1360, 1322, 1263, 1221, 1180, 1120, 1083, 993, 932, 884, 818, 743, 712, 662, 593, 521

Table S2. Analytical data for harmirins **4a-d**.

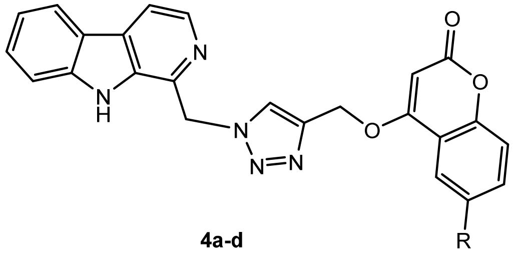

4a-d

Compd.	R	Yield (%)	Molecular Formula	M_r	MS	t_t (°C)	IR (υ/ cm^{-1})
**4a**	H	51	$C_{24}H_{17}N_5O_3$	423.43	424.1 (M + 1)$^+$	260.5–264.0 (decomp.)	3283, 1707, 1623, 1609, 1564, 1454, 1434, 1380, 1327, 1273, 1244, 1191, 1141, 1106, 1051, 938, 884, 824, 745, 728, 620
**4b**	CH_3	45	$C_{25}H_{19}N_5O_3$	437.46	438.1 (M + 1)$^+$	246.5–251.0 (decomp.)	3337, 3151, 3106, 3059, 1786, 1697, 1626, 1574, 1504, 1430, 1398, 1369, 1330, 1274, 1236, 1212, 1193, 1166, 1126, 1102, 1050, 969, 947, 839, 817, 762, 746, 724, 607, 585, 543, 510
**4c**	Cl	69	$C_{24}H_{16}ClN_5O_3$	457.87	458.0 (M + 1)$^+$	263.5–265.0 (decomp.)	3253, 3159, 1684, 1616, 1603, 1561, 1485, 1458, 1431, 1359, 1325, 1265, 1229, 1194, 1146, 1112, 1058, 939, 895, 818, 738, 596, 532
**4d**	F	56	$C_{24}H_{16}FN_5O_3$	441.42	442.1 (M + 1)$^+$	239.5–241.0 (decomp.)	3334, 3059, 1732, 1718, 1626, 1572, 1492, 1453, 1432, 1371, 1323, 1255, 1215, 1183, 1130, 1082, 1051, 977, 950, 937, 878, 827, 796, 746, 723, 622, 542

Table S3. Analytical data for harmirins **5a-d**.

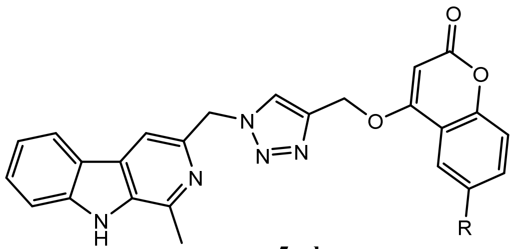

5a-d

**Compd.**	**R**	**Yield (%)**	**Molecular Formula**	***M*$_r$**	**MS**	***t*$_t$ (°C)**	**IR (*υ*/ cm^{-1})**
**5a**	H	64	$C_{25}H_{19}N_5O_3$	437.46	438.4 (M + 1)$^+$	149.5–153.5	3292, 1686, 1621, 1566, 1493, 1455, 1357, 1274, 1234, 1191, 1141, 1109, 1051, 937, 881, 812, 767, 731, 647, 588
**5b**	CH_3	45	$C_{26}H_{21}N_5O_3$	451.49	452.1 (M + 1)$^+$	263.0–267.5 (decomp.)	3149, 3092, 1721, 1629, 1576, 1499, 1431, 1384, 1355, 1323, 1281, 1250, 1208, 1132, 1102, 1059, 936, 902, 857, 821, 795, 737, 584, 533
**5c**	Cl	59	$C_{25}H_{18}ClN_5O_3$	471.90	472.0 (M + 1)$^+$	254.0–257.5 (decomp.)	3259, 3149, 3091, 1720, 1623, 1563, 1500, 1427, 1355, 1310, 1245, 1186, 1149, 1117, 1058, 931, 857, 825, 795, 735, 705, 677, 584, 532
**5d**	F	37	$C_{25}H_{18}FN_5O_3$	455.45	456.1 (M + 1)$^+$	259.0–261.5 (decomp.)	3151, 3095, 1715, 1630, 1575, 1499, 1453, 1366, 1320, 1254, 1216, 1176, 1131, 1081, 1055, 968, 922, 877, 827, 737, 704, 678, 588, 563

Table S4. Analytical data for harmirins **11a-d**.

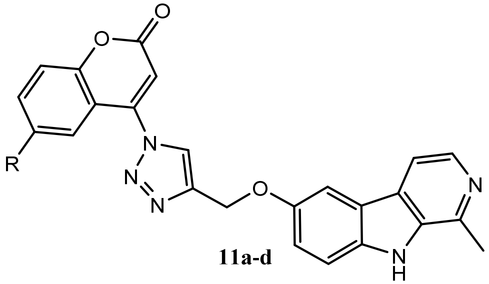

11a-d

Compd.	R	Yield (%)	Molecular Formula	M_r	MS	t_t (°C)	IR (υ/ cm^{-1})
**11a**	H	63	$C_{24}H_{17}N_5O_3$	423.13	424.1 (M + 1)$^+$	235.5–238.0 (decomp.)	3349, 3035, 1726, 1606, 1572, 1499, 1443, 1391, 1360, 1291, 1256, 1202, 1133, 1106, 1037, 1003, 944, 862, 808, 765, 701, 650, 616, 524
**11b**	CH_3	26	$C_{25}H_{19}N_5O_3$	437.46	438.1 (M + 1)$^+$	235.0–242.0 (decomp.)	3340, 3036, 1725, 1623, 1572, 1499, 1465, 1405, 1378, 1282, 1256, 1199, 1132, 1105, 1040, 1009, 945, 864, 807, 701, 610, 528
**11c**	Cl	26	$C_{24}H_{16}ClN_5O_3$	457.87	458.0 (M + 1)$^+$	220.0–225.0 (decomp.)	3245, 3165, 1731, 1620, 1558, 1500, 1479, 1452, 1401, 1344, 1284, 1261, 1230, 1213, 1185, 1111, 1052, 1013, 947, 827, 810, 682, 619, 558
**11d**	F	34	$C_{24}H_{16}FN_5O_3$	441.42	442.1 (M + 1)$^+$	224.5–225.5 (decomp.)	3357, 3028, 1730, 1527, 1512, 1488, 1464, 1416, 1353, 1291, 1261, 1238, 1202, 1159, 1074, 1035, 989, 942, 871, 808, 719, 700, 610, 523

Table S5. Analytical data for harmirins **12a-d**.

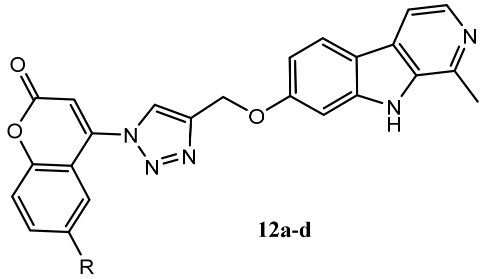

12a-d

**Compd.**	**R**	**Yield (%)**	**Molecular Formula**	M_r	**MS**	t_t **(°C)**	**IR (υ/ cm^{-1})**
**12a**	H	53	$C_{24}H_{17}N_5O_3$	423.43	424.1 (M + 1)$^+$	223.5–225.5 (decomp.)	3287, 3177, 3136, 3090, 3025, 2864, 1697, 1608, 1558, 1484, 1439, 1382, 1351, 1275, 1233, 1177, 1138, 1106, 1056, 1003, 964, 872, 820, 738, 651, 615, 572, 488
**12b**	CH_3	57	$C_{25}H_{19}N_5O_3$	437.46	438.1 (M + 1)$^+$	225.5–229.5 (decomp.)	3286, 3149, 3113, 3067, 3013, 2926, 2861, 1723, 1630, 1570, 1483, 1452, 1357, 1323, 1276, 1233, 1181, 1137, 1105, 1048, 1012, 946, 890, 801, 733, 610, 568, 519
**12c**	Cl	40	$C_{24}H_{16}ClN_5O_3$	457.87	458.0 (M + 1)$^+$	244.0–247.0 (decomp.)	3149, 3090, 3030, 2991, 1619, 1601, 1569, 1484, 1447, 1369, 1322, 1217, 1137, 1097, 1058, 1021, 910, 859, 805, 734, 669, 590, 540, 481
**12d**	F	39	$C_{24}H_{16}FN_5O_3$	441.42	442.0 (M + 1)$^+$	249.0–251.0 (decomp.)	3275, 3175, 3141, 3095, 3067, 3012, 1718, 1626, 1566, 1483, 1456, 1415, 1382, 1345, 1275, 1231, 1169, 1103, 1056, 1006, 966, 869, 806, 735, 614, 569, 528, 491

Table S6. Analytical data for harmirins **13a-d**.

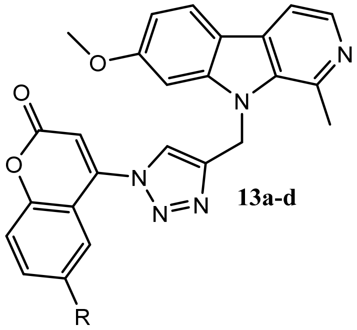

13a-d

Compd.	R	Yield (%)	Molecular Formula	M_r	MS	t_t (°C)	IR (υ/ cm^{-1})
**13a**	H	43	$C_{25}H_{19}N_5O_3$	437.46	438.1 (M + 1)$^+$	239.0–243.5 (decomp.)	3144, 3086, 3062, 2837, 1760, 1717, 1622, 1564, 1494, 1439, 1406, 1349, 1237, 1167, 1104, 1041, 948, 870, 813, 768, 646
**13b**	CH_3	53	$C_{26}H_{21}N_5O_3$	451.49	452.1 (M + 1)$^+$	253.0–255.0 (decomp.)	3145, 3096, 3058, 3000, 1728, 1622, 1568, 1496, 1445, 1408, 1367, 1336, 1284, 1243, 1195, 1165, 1137, 1039, 1010, 945, 889, 813, 748, 720, 649, 610, 550
**13c**	Cl	44	$C_{25}H_{18}ClN_5O_3$	471.90	472.0 (M + 1)$^+$	233.0–239.5 (decomp.)	3136, 3080, 2997, 2961, 1729, 1624, 1562, 1445, 1410, 1344, 1256, 1221, 1169, 1120, 1041, 971, 930, 816, 720, 682, 644, 597, 556, 512
**13d**	F	49	$C_{25}H_{18}FN_5O_3$	455.45	456.1 (M + 1)$^+$	235.0–237.0 (decomp.)	3441, 3127, 3072, 2955, 2836, 1728, 1628, 1574, 1490, 1461, 1440, 1406, 1348, 1301, 1256, 1235, 1197, 1163, 1041, 1025, 1008, 945, 887, 827, 728, 714, 611, 523

Table S7. ^{1}H and ^{13}C NMR spectroscopic data for *O*-alkylated coumarin **1d**.

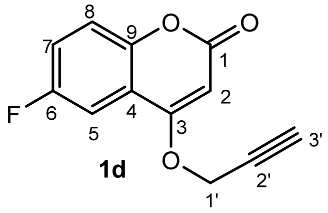

Compd.	^{1}H NMR (DMSO-d_6, δ ppm)	^{13}C NMR (DMSO-d_6, δ ppm)
**1d**	7.60-7.48 (m, 3H, 5, 7, 8), 6.04 (s, 1H, 2), 5.12 (s, 1H, 1'), 3.85 (t, 1H, 3', J = 2.39 Hz)	162.81 (3), 161.08 (1), 158.05 (d, 6, J_1 = 242.14 Hz), 149.11 (9), 120.32 (d, 7, J_2 = 29.28 Hz), 118.80-118.71 (d, 8, J_3 = 8.36 Hz), 118.76 (d, 4, J_3 = 8.37 Hz), 115.98 (d, 5, J_2 = 25.1 Hz), 92.49 (2), 80.33 (2'), 57.66 (1')

Table S8. ^{1}H and ^{13}C NMR spectroscopic data for harmirins **4a-d**.

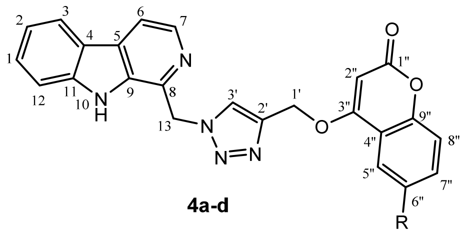

4a-d

Compd.	R	**^{1}H NMR (DMSO-d_6, δ ppm)**	**^{13}C NMR (DMSO-d_6, δ ppm)**
**4a**	H	11.99 (s, 1H, 10), 8.51 (s, 1H, 3'), 8.31 (d, 1H, 7, *J* = 5.17 Hz), 8.27 (d, 1H, 3, *J* = 7.85 Hz), 8.14 (d, 1H, 6, *J* = 5.14 Hz), 7.73 (dd, 1H, 5'', *J* = 1.64, 7.89 Hz), 7.69 (d, 1H, 12, *J* = 8.22 Hz), 7.66-7.64 (ddd, 1H, 7'', *J* = 1.65, 7.22, 8.63 Hz), 7.62-7.59 (ddd, 1H, 1, *J* = 1.21, 6.97, 8.22 Hz), 7.41 (dd, 1H, 8'', *J* = 1.03, 8.38 Hz), 7.34-7.28 (m, 2H, 2, 6''), 6.18, 6.15 (2s, 3H, 1', 2''), 5.44 (s, 2H, 13)	164.37 (3''), 161.55 (1''), 152.76 (9''), 140.87, 140.71 (8, 11) 137.94 (2'), 137.88 (7), 133.82 (9), 132.80 (7''), 128.74 (5), 128.58 (1), 126.27 (3'), 124.21 (6''), 122.86 (8''), 121.93 (3), 120.74 (4), 119.67 (2), 116.46 (5''), 115.08 (4''), 114.88 (6), 112.07 (12), 91.34 (2''), 62.76 (1'), 51.29 (13)
**4b**	$^{10''}CH_3$	11.99 (s, 1H, 10), 8.50 (s, 1H, 3'), 8.31 (d, 1H, 7, *J* = 5.14 Hz), 8.27 (d, 1H, 3, *J* = 7.80 Hz), 8.14 (d, 1H, 6, *J* = 5.16 Hz), 7.68 (d, 1H, 12, *J* = 0.92, 8.24 Hz), 7.62-7.59 (ddd, 1H, 1, *J* = 1.21, 7.03, 8.21 Hz), 7.50 (s, 1H, 5''), 7.45 (dd, 1H, 7'', *J* = 2.65, 8.82 Hz), 7.30-7.28 (m, 2H, 2, 8''), 6.15, 6.14 (2s, 3H, 1', 2''), 5.42 (s, 2H, 13), 2.33 (s, 3H, 10'')	164.36 (3''), 161.69 (1''), 150.92 (9''), 140.85, 140.70 (8, 11), 137.93 (2'), 137.87 (7), 133.82 (9), 133.62 (7''), 133.56 (6''), 128.74 (5), 128.58 (1), 126.32 (3'), 122.27 (5''), 121.93 (3), 120.74 (4), 119.67 (2), 116.28 (8''), 114.88 (6), 114.76 (4''), 112.07 (12), 91.27 (2''), 62.67 (1'), 51.28 (13), 20.28 (10'')
**4c**	Cl	11.98 (s, 1H, 10), 8.52 (s, 1H, 3'), 8.31 (d, 1H, 7, *J* = 5.18 Hz), 8.27 (d, 1H, 3, *J* = 7.85 Hz), 8.13 (d, 1H, 6, *J* = 5.14 Hz), 7.70-7.65 (m, 3H, 12, 5'', 7''), 7.60 (t, 1H, 1, *J* = 7.60 Hz), 7.46 (d, 1H, 8'', *J* = 8.83 Hz), 7.29 (t, 1H, 2, *J* = 7.45 Hz), 6.25 (s, 1H, 2''), 6.15 (s, 2H, 1'), 5.44 (s, 3H, 13)	163.19 (3''), 161.09 (1''), 151.41 (9''), 140.74, 140.71 (8, 11), 137.93 (2'), 137.87 (7), 133.81 (9), 132.51 (7''), 128.74 (5), 128.58 (1), 128.27 (6''), 126.33 (3'), 121.93 (3, 8''), 120.74 (4), 119.67 (2), 118.67 (5''), 116.56 (4''), 114.88 (6), 112.07 (12), 92.24 (2''), 63.06 (1'), 51.32 (13)
**4d**	F	11.98 (s, 1H, 10), 8.53 (s, 1H, 3'), 8.30 (d, 1H, 7, *J* = 5.18 Hz), 8.27 (d, 1H, 3, *J* = 7.84 Hz), 8.13 (d, 1H, 6, *J* = 5.13 Hz), 7.68 (d, 1H, 12, *J* = 8.20 Hz), 7.61-7.59 (ddd, 1H, 1, *J* = 1.23, 7.03, 8.23 Hz), 7.56-7.52 (td, 1H, 7'', *J* = 3.07, 8.60 Hz), 7.49-7.44 (m, 2H, 5'', 8''), 7.29 (t, 1H, 2, *J* = 7.40 Hz), 6.25 (s, 1H, 2''), 6.15 (s, 2H, 1'), 5.45 (s, 3H, 13)	163.51 (3''), 161.32 (1''), 157.99 (d, 6'', J_1 = 222.56 Hz), 149.11 (9'), 140.80, 140.70 (8, 11), 137.93 (2'), 137.86 (7), 133.80 (9), 128.73 (5), 128.57 (1), 126.25 (3'), 121.92 (3), 120.73 (4), 120.30 (d, 7'', J_2 = 20.69 Hz), 119.66 (2), 118.68 (d, 8'', J_3 = 7.88 Hz), 116.17 (d, 4'', J_3 = 11.83 Hz), 114.87 (6), 112.06 (12), 108.42 (d, 5'', J_2 = 29.57 Hz), 92.17 (2''), 63.05 (1'), 51.29 (13)

Table S9. ^{1}H and ^{13}C NMR spectroscopic data for harmirins **5a-d**.

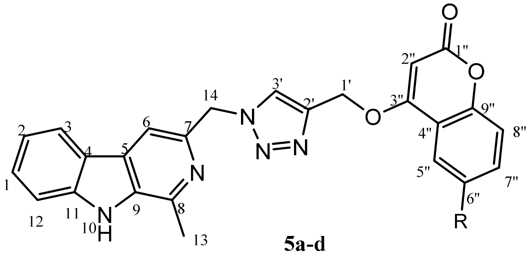

5a-d

Compd.	R	^{1}H NMR (DMSO-d_6, δ ppm)	^{13}C NMR (DMSO-d_6, δ ppm)
**5a**	H	11.67 (s, 1H, 10), 8.46 (s, 1H, 3'), 8.17 (d, 1H, 3, *J* = 7.85 Hz), 8.00 (s, 1H, 6), 7.73 (dd, 1H, 5'', *J* = 1.30, 7.90 Hz), 7.67-7.59 (m, 2H, 7'', 8''), 7.54 (t, 1H, 1, *J* = 7.60 Hz), 7.40 (d, 1H, 12, *J* = 8.23 Hz), 7.32 (t, 1H, 6'', *J* = 7.56 Hz), 7.24 (t, 1H, 2, *J* = 7.38 Hz), 6.17 (s, 1H, 2''), 5.81 (s, 2H, 1'), 5.43 (s, 2H, 14), 2.75 (s, 3H, 13)	164.34 (3''), 161.53 (1''), 152.76 (9''), 142.43, 142.18 (7, 2'), 140.96, 140.77 (8, 11), 134.00 (9), 132.79 (7''), 128.10 (1), 127.60 (5), 125.64 (3'), 124.20 (6''), 122.87 (8''), 121.69 (3), 120.91 (4), 119.46 (2), 116.45 (5''), 115.08 (4''), 112.09, 112.00 (6, 12), 91.32 (2''), 62.78 (1'), 55.28 (14), 20.38 (13)
**5b**	$^{10''}CH_3$	11.67 (s, 1H, 10), 8.45 (s, 1H, 3'), 8.13 (d, 1H, 3, *J* = 7.87 Hz), 8.00 (s, 1H, 6), 7.61-7.52 (m, 2H, 5'', 7''), 7.50 (br. s, 1H, 1), 7.45 (dd, 1H, 12, *J* = 1.81, 8.46 Hz), 7.29 (d, 1H, 8'', *J* = 8.41 Hz), 7.26-7.22 (m, 1H, 2), 6.14 (s, 1H, 2''), 5.81 (s, 2H, 1'), 5.41 (s, 2H, 14), 2.75 (s, 3H, 13), 2.33 (s, 3H, 10'')	164.34 (3''), 161.68 (1''), 150.92 (9''), 142.47, 142.18 (7, 2'), 140.94, 140.77 (8, 11), 134.00 (9), 133.61 (7''), 133.55 (6''), 128.10 (1), 127.60 (5), 125.71 (3'), 122.29 (8''), 121.68 (3), 120.91 (4), 119.45 (2), 116.26 (5''), 114.76 (4''), 120.09, 111.98 (6, 12), 91.25 (2''), 62.70 (1'), 55.27 (14), 20.38, 20.26 (13, 10'')
**5c**	Cl	11.67 (s, 1H, 10), 8.48 (s, 1H, 3'), 8.17 (d, 1H, 3, *J* = 7.88 Hz), 7.99 (s, 1H, 6), 7.69 (dd, 1H, 7'', *J* = 2.63, 8.84 Hz), 7.66 (d, 1H, 5'', *J* = 2.53 Hz), 7.60 (d, 1H, 12, *J* = 8.18 Hz), 7.55-7.53 (ddd, 1H, 1, *J* = 1.19, 6.97, 8.21 Hz), 7.46-7.44 (d, 1H, 8'', *J* = 8.87 Hz), 7.25-7.22 (ddd, 1H, 2, *J* = 1.05, 6.98, 7.98 Hz), 6.24 (s, 1H, 2''), 5.81 (s, 2H, 1'), 5.44 (s, 2H, 14), 2.75 (s, 3H, 13)	163.17 (3''), 161.09 (1''), 151.41 (9''), 142.48, 142.17 (7, 2'), 140.84, 140.78 (8, 11), 134.00 (9), 132.49 (7''), 128.26 (6''), 128.09 (1), 127.60 (5), 125.73 (3'), 121.94, 121.69 (3, 8''), 120.91 (4), 119.45 (2), 118.65 (5''), 116.57 (4''), 112.09, 111.93 (6, 12), 92.22 (2''), 63.10 (1'), 55.28 (14), 20.39 (13)
**5d**	F	11.67 (s, 1H, 10), 8.48 (s, 1H, 3'), 8.17 (d, 1H, 3, *J* = 8.27 Hz), 7.99 (s, 1H, 6), 7.60 (d, 1H, 7'', *J* = 8.18 Hz), 7.56-7.51 (m, 2H, 12, 5''), 7.48-7.45 (m, 2H, 1, 8''), 7.25-7.22 (ddd, 1H, 2, *J* = 1.04, 6.99, 8.02 Hz), 6.24 (s, 1H, 2''), 5.81 (s, 2H, 1'), 5.44 (s, 2H, 14), 2.75 (s, 3H, 13)	163.50 (3''), 161.32 (1''), 157.99 (d, 6'', J_1 = 236.63 Hz), 149.10 (9''), 142.49, 142.17 (7, 2'), 140.91, 140.78 (8, 11), 134.00 (9), 128.10 (1), 127.60 (5), 125.65 (3'), 121.69 (3), 120.91 (4), 120.17 (d, 7'', J_2 = 26.32 Hz), 119.45 (2), 118.67 (d, 8'', *J* = 9.06 Hz), 116.18 (d, 4', J_3 = 8.15 Hz), 112.10, 111.94 (6, 12), 108.44 (d, 5'', J_2 = 24.70 Hz), 92.16 (2''), 63.09 (1'), 55.28 (14), 20.38 (13)

Table S10. ^{1}H and ^{13}C NMR spectroscopic data for harmirins **11a-d**.

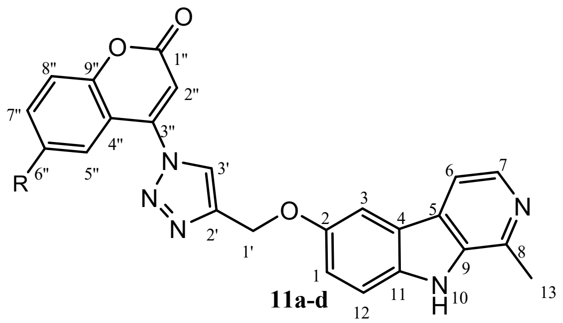

11a-d

Compd.	R	^{1}H NMR (DMSO-d_6, δppm)	^{13}C NMR (DMSO-d_6, δppm)
**11a**	H	11.43 (s, 1H, 10), 8.99 (s, 1H, 3'), 8.19 (d, 1H, 7, *J* = 5.33 Hz), 7.98 (s, 1H, 3), 7.93 (d, 1H, 6, *J* = 5.32 Hz), 7.84-7.76 (m, 2H, 5'', 7''), 7.60-7.53 (m, 2H, 12, 8''), 7.42 (t, 1H, 6'', *J* = 7.69 Hz), 7.30 (dd, 1H, 1, *J* = 2.45, 8.85 Hz), 7.00 (s, 1H, 2''), 5.40 (s, 2H, 1'), 2.75 (s, 3H, 13)	159.47 (1''), 153.66 (3''), 151.87 (2), 145.91 (9''), 143.89 (2'), 142.23 (8), 136.91 (7), 135.60 (11), 135.10 (9), 133.47 (7''), 126.75 (4), 126.37 (3'), 125.47 (5''), 124.98 (6''), 121.37 (5), 118.46 (6), 117.19 (8''), 114.36 (4''), 112.85 (1, 12), 110.66 (2''), 105.33 (3), 61.62 (1'), 20.36 (13)
**11b**	$^{10''}CH_3$	11.42 (s, 1H, 10), 8.97 (s, 1H, 3'), 8.18 (d, 1H, 7, *J* = 5.28 Hz), 7.98 (s, 1H, 3), 7.93 (d, 1H, 6, *J* = 5.31 Hz), 7.60-7.48 (m, 4H, 12, 5'', 7'', 8''), 7.30 (dd, 1H, 1, *J* = 2.45, 8.84 Hz), 6.95 (s, 1H, 2''), 5.40 (s, 1H, 1'), 2.75 (s, 3H, 13), 2.34 (s, 3H, 10'')	159.57 (1''), 151.85 (2), 145.91 (9''), 143.87 (2'), 142.23 (8), 136.94 (7), 135.58 (11), 135.11 (9), 134.41 (6''), 134.35 (7''), 126.74 (4), 126.38 (3'), 124.80 (5''), 121.38 (5), 118.42 (6), 117.00 (8''), 114.08 (4'), 112.85, 112.72 (1, 12), 110.71 (2''), 105.29 (3), 61.60 (1'), 20.41, 20.37 (13, 10'')
**11c**	Cl	11.43 (s, 1H, 10), 9.01 (s, 1H, 3'), 8.18 (d, 1H, 7, *J* = 5.33 Hz), 7.97 (s, 1H, 3), 7.92-7.91 (m, 2H, 6, 5''), 7.83 (d, 1H, 7'', *J* = 8.90 Hz), 7.64 (d, 1H, 12, *J* = 8.89 Hz), 7.55 (d, 1H, 8'', *J* = 8.77 Hz), 7.29 (d, 1H, 1, *J* = 8.83 Hz), 7.09 (s, 1H, 2''), 5.40 (s, 2H, 1'), 2.74 (s, 3H, 13)	159.05 (1''), 152.34 (3''), 151.81 (2), 144.58 (9''), 144.04 (2'), 142.22 (8), 136.96 (7), 135.55 (11), 135.08 (9), 133.00 (7''), 128.78 (6''), 126.67 (4), 126.24 (3'), 124.78 (5''), 121.36 (5), 119.23 (6), 118.32 (8''), 115.61 (4''), 112.81, 112.67 (1, 12), 111.26 (2''), 105.27 (3), 61.59 (1'), 20.36 (13)
**11d**	F	11.41 (s, 1H, 10), 9.02 (s, 1H, 3'), 8.18 (d, 1H, 7, *J* = 5.29 Hz), 7.97 (s, 1H, 3), 7.92 (d, 1H, 6, *J* = 5.26 Hz), 7.73-7.66 (m, 3H, 12, 5'', 7''), 7.54 (d, 1H, 8'', *J* = 8.80 Hz), 7.29 (dd, 1H, 1, *J* = 2.53, 8.82 Hz), 7.09 (s, 1H, 2''), 5.40 (s, 2H, 1'), 2.74 (s, 3H, 13)	159.30 (1''), 158.05 (d, 6'', J_1 = 241.22 Hz), 151.86 (2). 150.11 (3''), 144.93 (d, 9'', J_4 = 2.52 Hz), 144.05 (2'), 142.25 (8), 137.00 (7), 135.56 (11), 135.11 (9), 126.69 (4), 126.23 (3'), 121.39 (5), 120.82 (d, 7'', J_2 = 25.77 Hz), 119.33 (d, 8'', J_3 = 8.37 Hz), 118.35 (6), 115.20 (d, 4'', J_3 = 7.36 Hz), 112.83 (1, 12), 111.44 (d, 5'', J_2 = 25.53 Hz), 111.16 (2''), 105.26 (3), 61.62 (1'), 20.40 (13)

Table S11. ^{1}H and ^{13}C NMR spectroscopic data for harmirins **12a-d**.

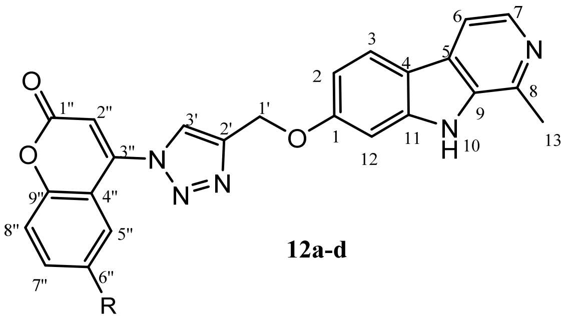

12a-d

Compd.	R	^{1}H NMR (DMSO-d_6, δppm)	^{13}C NMR (DMSO-d_6, δppm)
**12a**	H	11.52 (s, 1H, 10), 8.99 (s, 1H, 3'), 8.17 (d, 1H, 7, *J* = 5.27 Hz), 8.11 (d, 1H, 3, *J* = 8.62 Hz), 7.85-7.82 (m, 2H, 6, 5''), 7.79 (t, 1H, 7'', *J* = 8.00 Hz), 7.60 (d, 1H, 8'', *J* = 8.34 Hz), 7.45 (t, 1H, 6'', *J* = 7.66 Hz), 7.25 (s, 1H, 12), 7.00-6.97 (m, 2H, 2, 2''), 5.44 (s, 2H, 1'), 2.74 (s, 3H, 13)	159.45 (1''), 158.71 (1), 153.65 (3''), 145.90 (9''), 143.64 (2'), 141.84 (8), 141.30 (11), 137.57 (7), 134.61 (9), 133.48 (7''), 127.23 (5), 126.51 (3'), 125.45 (5''), 125.00 (6''), 122.77 (3), 117.19 (8''), 115.31 (4), 114.36 (4''), 112.04 (6), 110.72 (2), 109.46 (2''), 96.02 (12), 61.14 (1'), 20.23 (13)
**12b**	$^{10''}CH_3$	11.51 (s, 1H, 10), 8.97 (s, 1H, 3'), 8.16 (s, 1H, 7), 8.11 (d, 1H, 3'', *J* = 8.52 Hz), 7.84 (d, 1H, 6, *J* = 4.60 Hz), 7.60-7.48 (m, 3H, 5'', 7'', 8''), 7.24 (s, 1H, 12), 6.99-6.96 (m, 2H, 2, 2''), 5.45 (s, 2H, 1'), 2.74 (s, 3H, 13), 2.35 (s, 3H, 10'')	159.56 (1''), 158.68 (1), 151.85 (3''), 145.90 (9''), 143.61 (2'), 141.83 (8), 141.31 (11), 137.61 (7), 134.61 (9), 134.42 (6''), 134.36 (7''), 127.21 (5), 126.54 (3'), 124.77 (5''), 122.78 (3), 117.01 (8''), 115.32 (4), 114.08 (6), 112.04 (2), 110.78 (2''), 96.02 (12), 61.14 (1'), 20.43, 20.25 (13, 10'')
**12c**	Cl	11.51 (s, 1H, 10), 9.01 (s, 1H, 3'), 8.17 (d, 1H, 7, *J* = 5.30 Hz), 8.11 (d, 1H, 3, *J* = 8.65 Hz), 7.91 (s, 1H, 5''), 7.85-7.82 (m, 2H, 6, 7''), 7.64 (d, 1H, 8'', *J* = 8.88 Hz), 7.24 (s, 1H, 12), 7.10 (s, 1H, 2''), 6.98 (dd, 1H, 2, *J* = 2.13, 8.65 Hz), 5.45 (s, 2H, 1'), 2.74 (s, 3H, 13)	159.07 (1'), 158.68 (1), 152.37 (3''), 144.60 (9''), 143.79 (2'), 141.83 (8), 141.30 (11), 137.59 (7), 134.61 (9), 133.05 (7''), 128.82 (6''), 127.22 (5), 126.44 (3'), 124.78 (5''), 122.78 (3), 119.28 (8''), 115.65 (4''), 115.33 (4), 112.04 (6), 111.38 (2), 109.41 (2''), 96.02 (12), 61.13 (1'), 20.24 (13)
**12d**	F	11.48 (s, 1H, 10), 9.02 (s, 1H, 3'), 8.16 (d, 1H, 7, *J* = 5.24 Hz), 8.10 (d, 1H, 3, *J* = 8.61 Hz), 7.83 (d, 1H, 6, *J* = 5.28 Hz), 7.72-7.67 (m, 3H, 5'', 7'', 8''), 7.24 (s, 1H, 12), 7.09 (s, 1H, 2''), 6.98 (dd, 1H, 2, *J* = 2.27, 8.61 Hz), 5.44 (s, 2H, 1'), 2.73 (s, 3H, 13)	159.26 (1''), 158.55 (d, 6'', J_1 = 246.21 Hz), 158.64 (1), 150.10 (3''), 144.92 (d, 9'', J_4 = 2.92 Hz), 143.78 (2'), 141.77 (8), 141.36 (11), 137.72 (7), 134.62 (9), 127.12 (5), 126.38 (3'), 122.73 (3), 120.82 (d, 7'', J_2 = 31.71 Hz), 119.33 (d, 8'', J_3 = 10.57 Hz), 115.34 (4), 115.20 (d, 4''. J_3 = 10.57 Hz), 111.99 (6), 111.39 (d, 5'', J_2 = 31.70 Hz), 111.27 (2), 111.23 (2''), 96.01 (12), 61.14 (1'), 20.30 (13)

Table S12. ^1H and ^{13}C NMR spectroscopic data for harmirins **13a-d**.

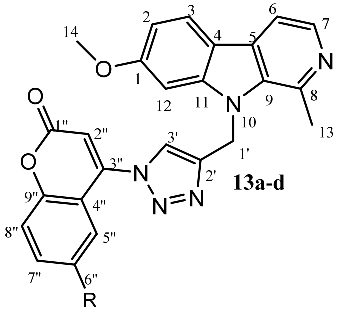

13a-d

Compd.	R	^1H NMR (DMSO-d_6, δ ppm)	^{13}C NMR (DMSO-d_6, δ ppm)
**13a**	H	8.78 (s, 1H, 3'), 8.21 (br. s, 1H, 7), 8.11 (d, 1H, 3, *J* = 8.60 Hz), 7.91 (d, 1H, 6, *J* = 4.71 Hz), 7.76-7.72 (m, 2H, 5'', 7''), 7.55 (dd, 1H, 8'', *J* = 0.95, 8.70 Hz), 7.41-7.37 (m, 2H, 12, 6''), 6.92-6.89 (m, 2H, 2, 2''), 6.03 (s, 2H, 1'), 3.92 (s, 3H, 14), 3.10 (s, 3H, 13)	160.62 (1), 159.41 (1''), 153.57 (3''), 145.69 (9''), 144.74 (2'), 142.72 (8), 141.18 (11), 138.13 (7), 134.87 (9), 133.37 (7''), 128.63 (5), 125.40 (3'), 124.90 (5'', 6''), 122.40 (3), 117.13 (8''), 114.52, 114.36 (4, 4''), 112.34 (6), 110.71 (2''), 109.47 (2), 94.16 (12), 55.66 (14), 23.30 (13)
**13b**	$^{10''}CH_3$	8.78 (s, 1H, 3'), 8.21 (d, 1H, 7, *J* = 5.16 Hz), 8.11 (d, 1H, 3, *J* = 8.60 Hz), 7.90 (d, 1H, 6, *J* = 5.17 Hz), 7.55 (dd, 1H, 5'', *J* = 1.48, 8.55 Hz), 7.45-7.40 (m, 3H, 12, 7'', 8''), 6.91 (dd, 1H, 2, *J* = 2.02, 8.60 Hz), 6.86 (s, 1H, 2''), 6.03 (s, 2H, 1'), 3.91 (s, 3H, 14), 3.11 (s, 3H, 13), 2.31 (s, 3H, 10'')	160.63 (1), 159.51 (1''), 151.77 (3''), 145.67 (9''), 144.69 (2'), 142.70 (8), 141.16 (11), 138.18 (7), 134.77 (9), 134.30 (6''), 134.24 (7''), 128.64 (5), 124.95 (3'), 124.64 (5''), 122.41 (3), 116.96 (8''), 114.53, 114.06 (4, 4''), 112.30 (6), 110.72 (2''), 109.45 (2), 94.16 (12), 55.66 (14), 54.91 (1'), 23.30 (13), 20.38 (10'')
**13c**	Cl	8.79 (s, 1H, 3'), 8.20 (d, 1H, 7, *J* = 5.19 Hz), 8.11 (d, 1H, 3, *J* = 8.60 Hz), 7.90 (d, 1H, 6, *J* = 5.19 Hz), 7.82-7.78 (m, 2H, 5'', 7''), 7.60 (d, 1H, 8'', *J* = 8.68 Hz), 7.39 (s, 1H, 12), 7.01 (s, 1H, 2''), 6.90 (dd, 1H, 2, *J* = 2.05, 8.60 Hz), 6.03 (s, 2H, 1'), 3.91 (s, 3H, 14), 3.09 (s, 3H, 13)	160.64 (1), 159.01 (1''), 152.27 (3''), 144.95 (9''), 144.41 (2'), 142.72 (8), 141.12 (11), 138.17 (7), 134.71 (9), 132.93 (7''), 128.73, 128.65 (5, 6''), 124.79, 124.67 (3', 5''), 122.39 (3), 119.22 (8''), 115.65, 114.50 (4, 4''), 112.29 (6), 111.36 (2), 109.48 (2''), 94.12 (12), 55.67 (14), 23.25 (13)
**13d**	F	8.81 (s, 1H, 3'), 8.21 (d, 1H, 7, *J* = 5.18 Hz), 8.12 (d, 1H, 3, *J* = 8.60 Hz), 7.92 (d, 1H, 6, *J* = 5.17 Hz), 7.65-7.61 (m, 3H, 5'', 7'', 8''), 7.39 (s, 1H, 12), 7.01 (s, 1H, 2''), 6.91 (dd, 1H, 2, *J* = 2.17, 8.56 Hz), 6.03 (s, 2H, 1'), 3.91 (s, 3H, 14), 3.09 (s, 3H, 13)	160.62 (1''), 159.24 (1), 158.49 (d, 6'', J_1 = 235.40 Hz), 150.02 (3''), 144.92, 144.75 (2', 9''), 142.70 (8), 141.15 (11), 138.18 (7), 134.73 (9), 128.63 (5), 124.77 (3'), 122.40 (3), 120.72 (d, 7'', J_2 = 29.19 Hz), 119.28 (d, 8'', J_3 = 8.63 Hz), 115.21 (d, 4'', J_3 = 10.88 Hz), 114.52 (4), 112.30 (6), 111.33 (d, 5'', J_2 = 24.99 Hz), 111.24 (2), 109.46 (2''), 94.14 (12), 55.65 (14), 23.27 (13)

Table S13. Properties of the harmirins calculated with Chemicalize.org program [34]. The Lipinski and Gelovani parameters..[a]

Compd.	Molecular Formula	Number of Atoms	MW	log *P*	H-bond Donor	H-bond Acceptor	Lipinski Score [b]	MR (cm^3/mol)	PSA (Å2)
**4a**	$C_{24}H_{17}N_5O_3$	49	423.43	2.701	1	5	4	128.89	94.92
**4b**	$C_{25}H_{19}N_5O_3$	52	437.46	3.215	1	5	4	133.93[c]	94.92
**4c**	$C_{24}H_{16}ClN_5O_3$	49	457.87	3.306	1	5	4	133.70[c]	94.92
**4d**	$C_{24}H_{16}FN_5O_3$	49	441.42	2.844	1	5	4	129.11	94.92
**5a**	$C_{25}H_{19}N_5O_3$	52	437.46	2.833	1	5	4	133.48[c]	94.92
**5b**	$C_{26}H_{21}N_5O_3$	55	451.49	3.346	1	5	4	138.52[c]	94.92
**5c**	$C_{25}H_{18}ClN_5O_3$	52	471.90	3.437	1	5	4	138.29[c]	94.92
**5d**	$C_{25}H_{18}FN_5O_3$	52	455.45	2.976	1	5	4	133.70[c]	94.92
**11a**	$C_{24}H_{17}N_5O_3$	49	423.43	2.718	1	5	4	128.90	94.92
**11b**	$C_{25}H_{19}N_5O_3$	52	437.46	3.231	1	5	4	133.94[c]	94.92
**11c**	$C_{24}H_{16}ClN_5O_3$	49	457.87	3.322	1	5	4	133.71[c]	94.92
**11d**	$C_{24}H_{16}FN_5O_3$	49	441.42	2.861	1	5	4	129.12	94.92
**12a**	$C_{24}H_{17}N_5O_3$	49	423.43	2.718	1	5	4	128.90	94.92
**12b**	$C_{25}H_{19}N_5O_3$	52	437.46	3.231	1	5	4	133.94[c]	94.92
**12c**	$C_{24}H_{16}ClN_5O_3$	49	457.87	3.322	1	5	4	133.71[c]	94.92
**12d**	$C_{24}H_{16}FN_5O_3$	49	441.42	2.861	1	5	4	129.12	94.92
**13a**	$C_{25}H_{19}N_5O_3$	52	437.46	2.942	0	5	4	133.08[c]	84.06
**13b**	$C_{26}H_{21}N_5O_3$	55	451.49	3.455	0	5	4	138.84[c]	84.06
**13c**	$C_{25}H_{18}ClN_5O_3$	52	471.90	3.546	0	5	4	138.60[c]	84.06
**13d**	$C_{25}H_{18}FN_5O_3$	52	455.45	3.084	0	5	4	134.01[c]	84.06

[a] Calculated with Chemicalize.org program [34]; [b] Out of 4; [c] Minimal aberrations of the rules; MR – molar refractivity, PSA – polar surface area.

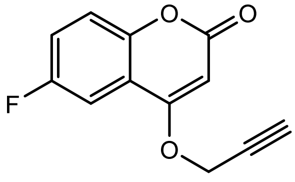

1d

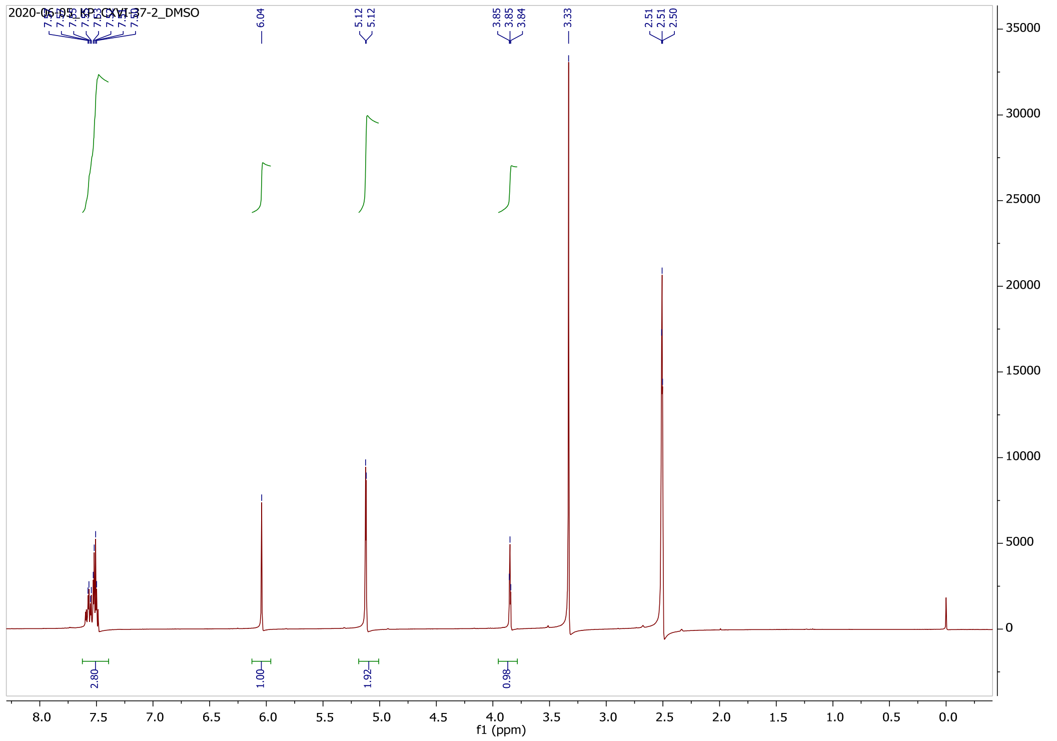
2020-06-05_KP-CXVI-37-2_DMSO
6.04
5.12
5.12
3.85
3.85
3.84
3.33
2.51
2.51
2.50
2.80
1.00
1.92
0.98
f1 (ppm)

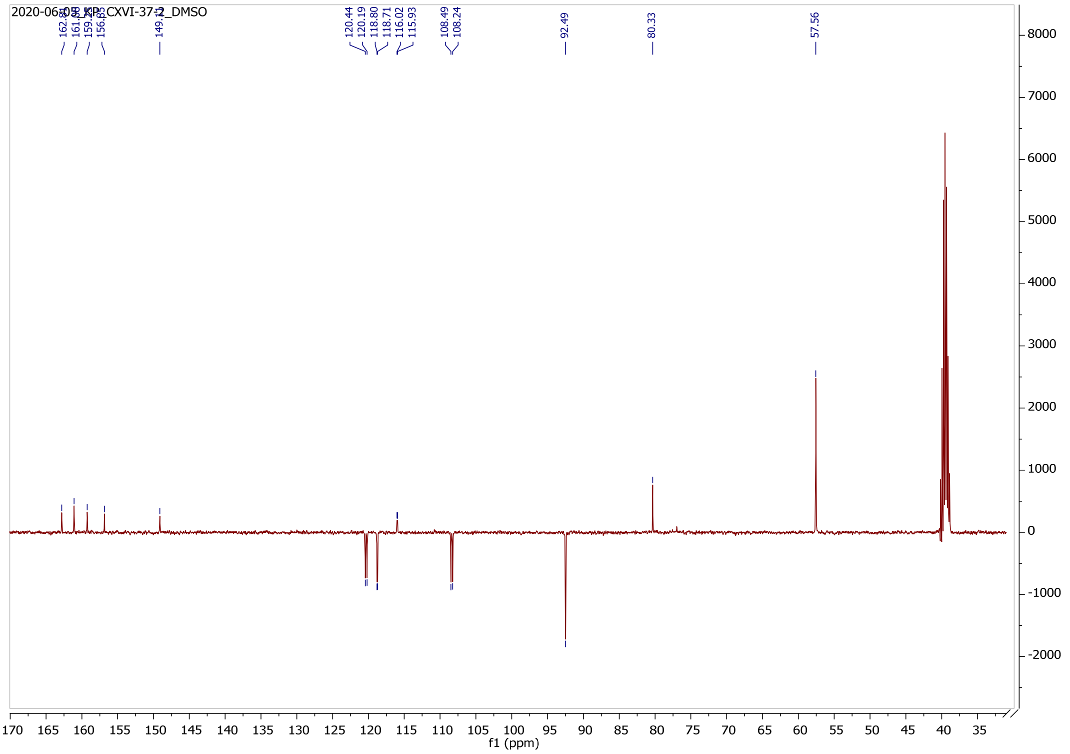
2020-06-05_KP-CXVI-37-2_DMSO
120.44
120.19
118.80
118.71
116.02
115.93
108.49
108.24
92.49
80.33
57.56
f1 (ppm)

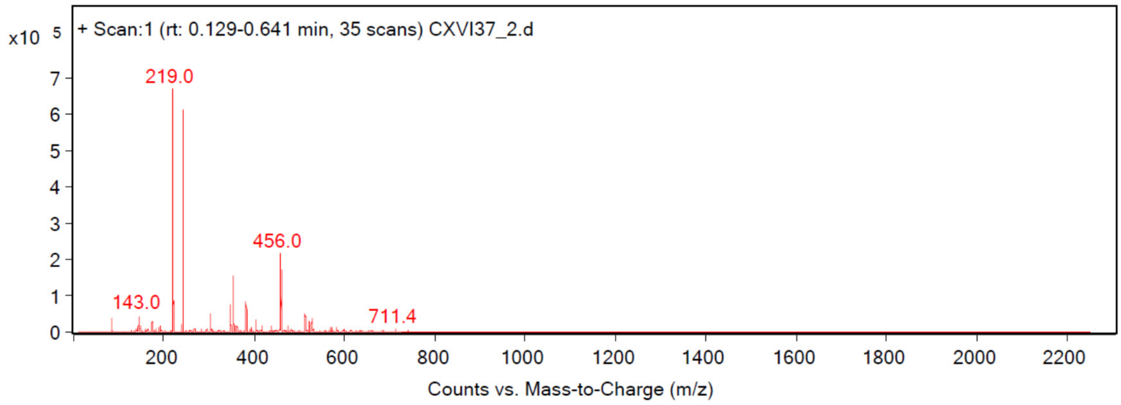
+ Scan:1 (rt: 0.129-0.641 min, 35 scans) CXVI37_2.d
x10 5
219.0
456.0
143.0
711.4
Counts vs. Mass-to-Charge (m/z)

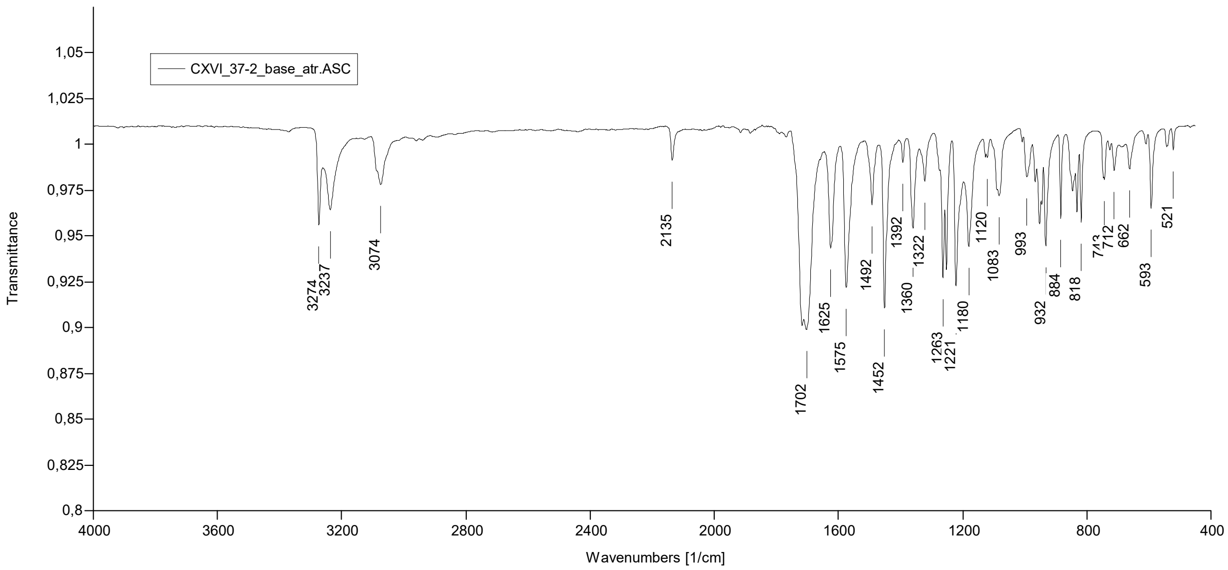
CXVI_37-2_base_atr.ASC
Transmittance
Wavenumbers [1/cm]
3274
3237
3074
2135
1702
1625
1575
1492
1452
1392
1360
1322
1263
1221
1180
1120
1083
993
932
884
818
712
662
593
521

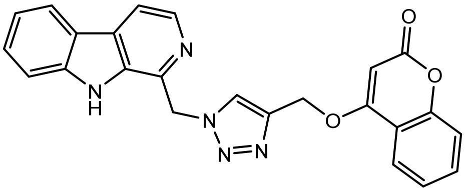
N
N
H
N
N=N
O
O
O

4a

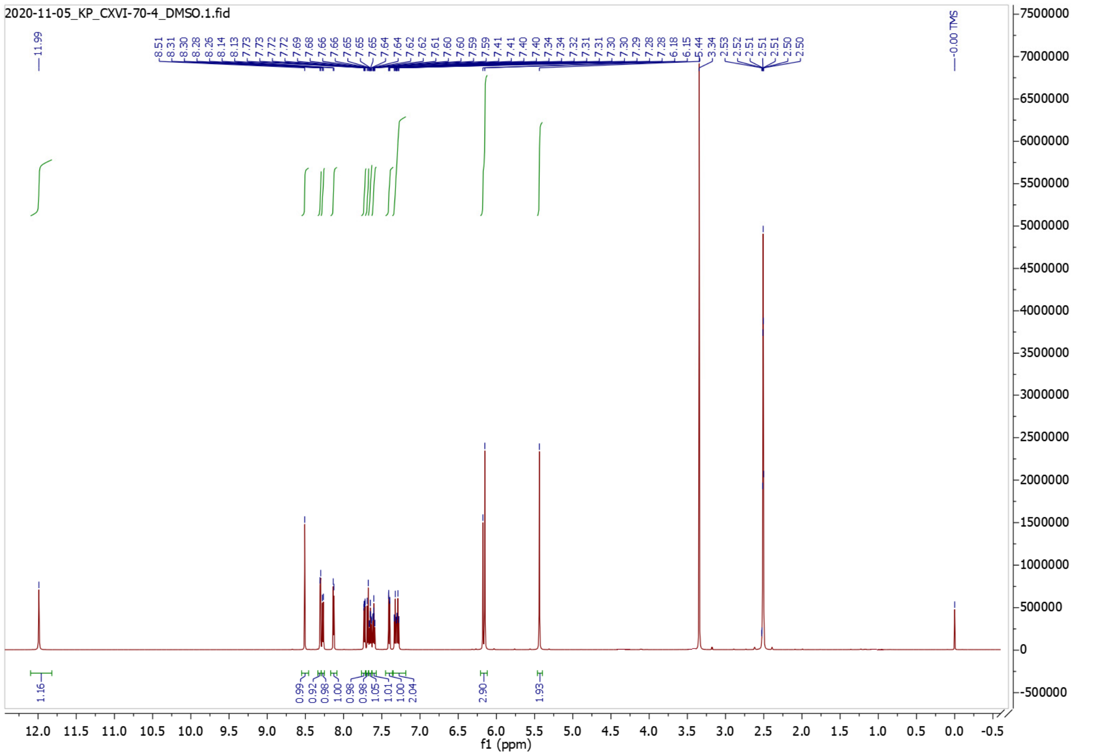
2020-11-05_KP_CXVI-70-4_DMSO.1.fid
11.99
8.51
8.31
8.30
8.28
8.26
8.14
8.13
7.73
7.73
7.72
7.72
7.69
7.68
7.66
7.66
7.65
7.65
7.65
7.64
7.64
7.62
7.62
7.61
7.60
7.60
7.59
7.59
7.41
7.41
7.40
7.40
7.34
7.34
7.32
7.31
7.30
7.30
7.29
7.28
7.28
6.18
6.15
5.44
3.34
2.53
2.52
2.51
2.51
2.51
2.50
2.50
0.00 TMS
1.16
0.99
0.92
0.98
1.00
0.98
0.98
1.05
1.01
1.00
2.04
2.90
1.93
f1 (ppm)

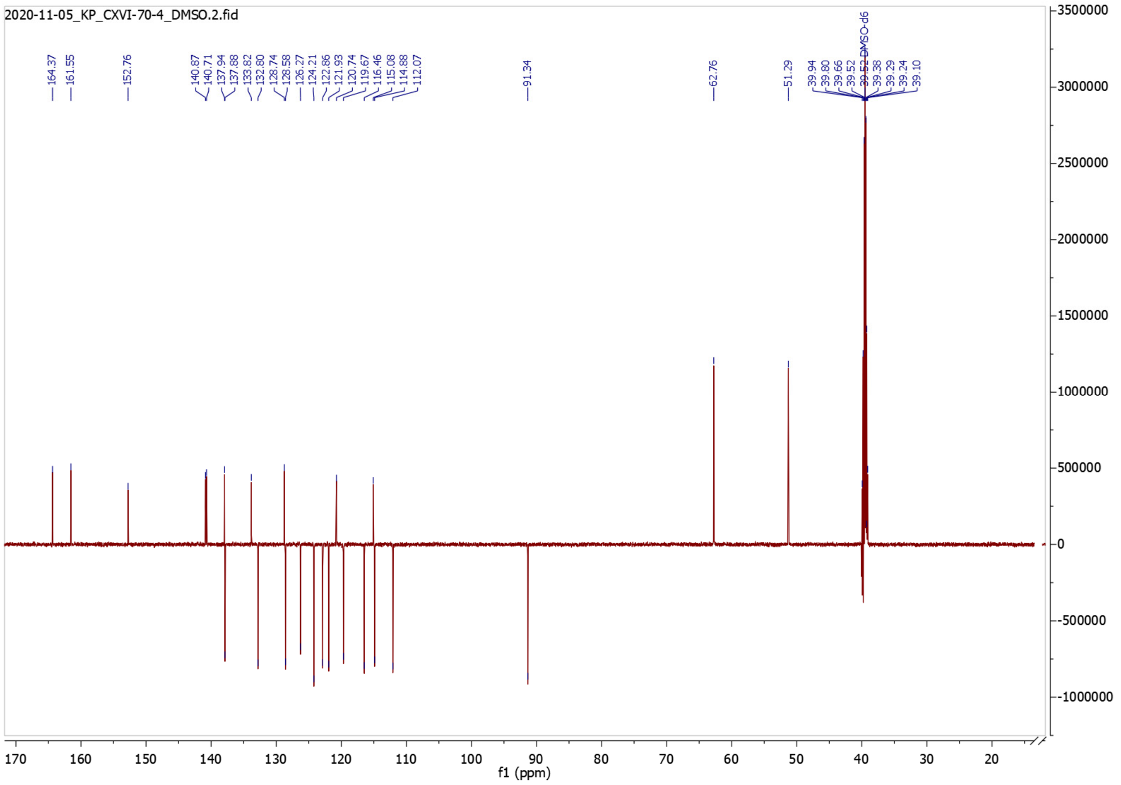
2020-11-05_KP_CXVI-70-4_DMSO.2.fid
164.37
161.55
152.76
140.87
140.71
137.94
137.88
133.82
132.80
128.74
128.58
126.27
124.21
122.86
121.93
120.74
119.67
116.46
115.08
114.88
112.07
91.34
62.76
51.29
39.94
39.80
39.66
39.52 DMSO-d6
39.38
39.29
39.24
39.10
f1 (ppm)

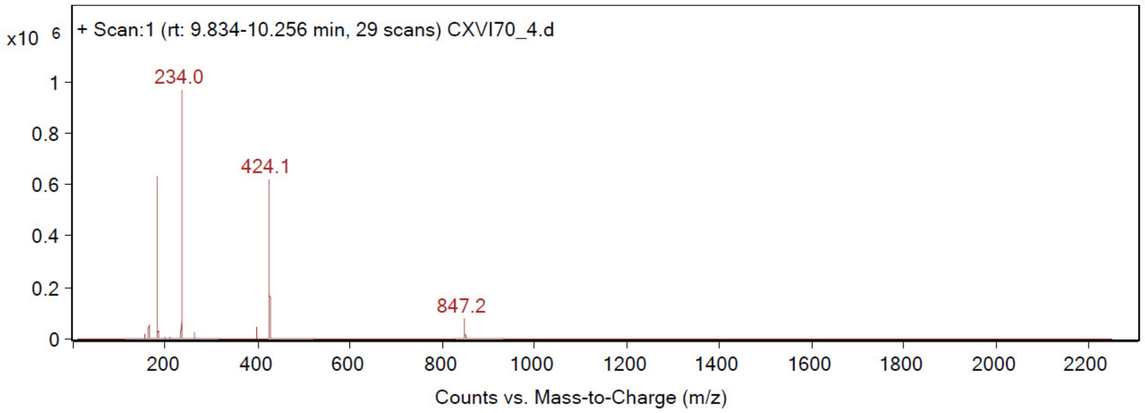
+ Scan:1 (rt: 9.834-10.256 min, 29 scans) CXVI70_4.d
234.0
424.1
847.2
Counts vs. Mass-to-Charge (m/z)

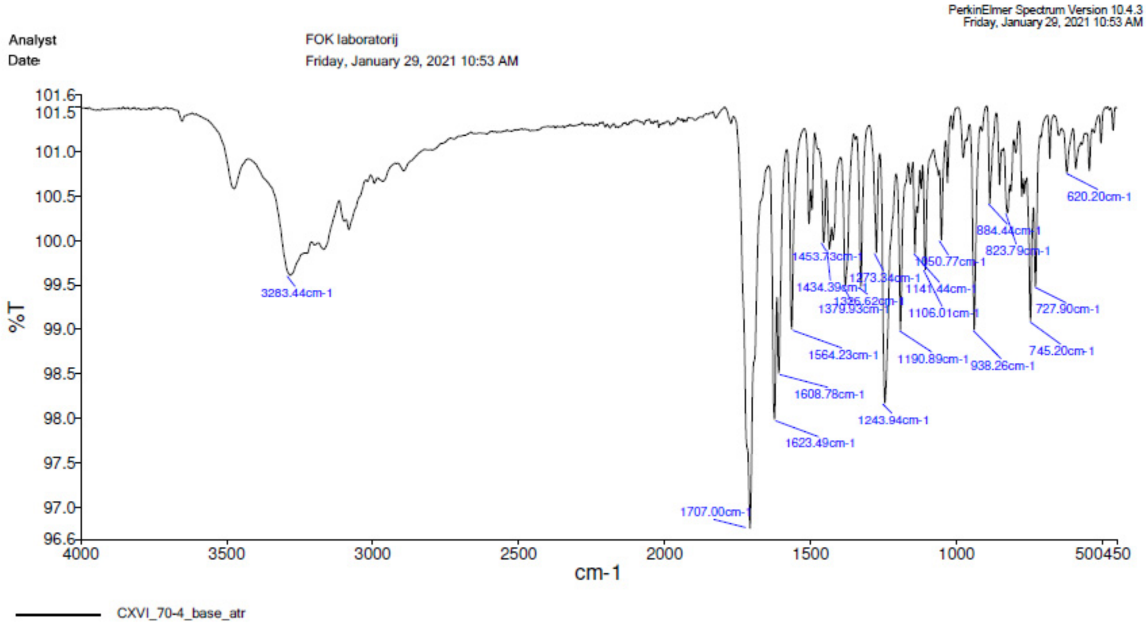
PerkinElmer Spectrum Version 10.4.3
Friday, January 29, 2021 10:53 AM
Analyst FOK laboratorij
Date Friday, January 29, 2021 10:53 AM
%T
cm-1
3283.44cm-1
1707.00cm-1
1623.49cm-1
1608.78cm-1
1564.23cm-1
1453.73cm-1
1434.39cm-1
1273.34cm-1
1243.94cm-1
1190.89cm-1
1141.44cm-1
1106.01cm-1
1050.77cm-1
938.26cm-1
884.44cm-1
823.79cm-1
745.20cm-1
727.90cm-1
620.20cm-1
CXVI_70-4_base_atr

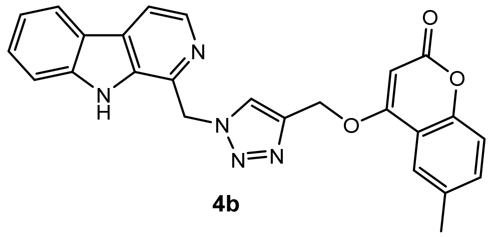

4b

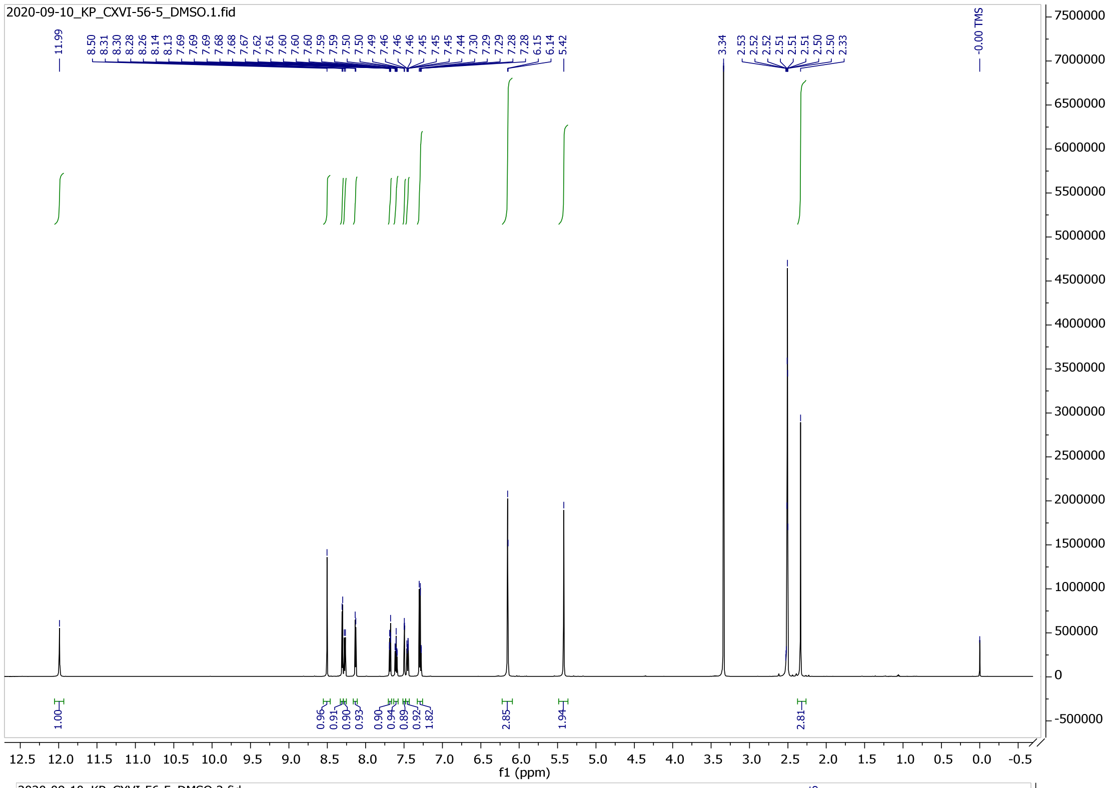
2020-09-10_KP_CXVI-56-5_DMSO.1.fid
f1 (ppm)

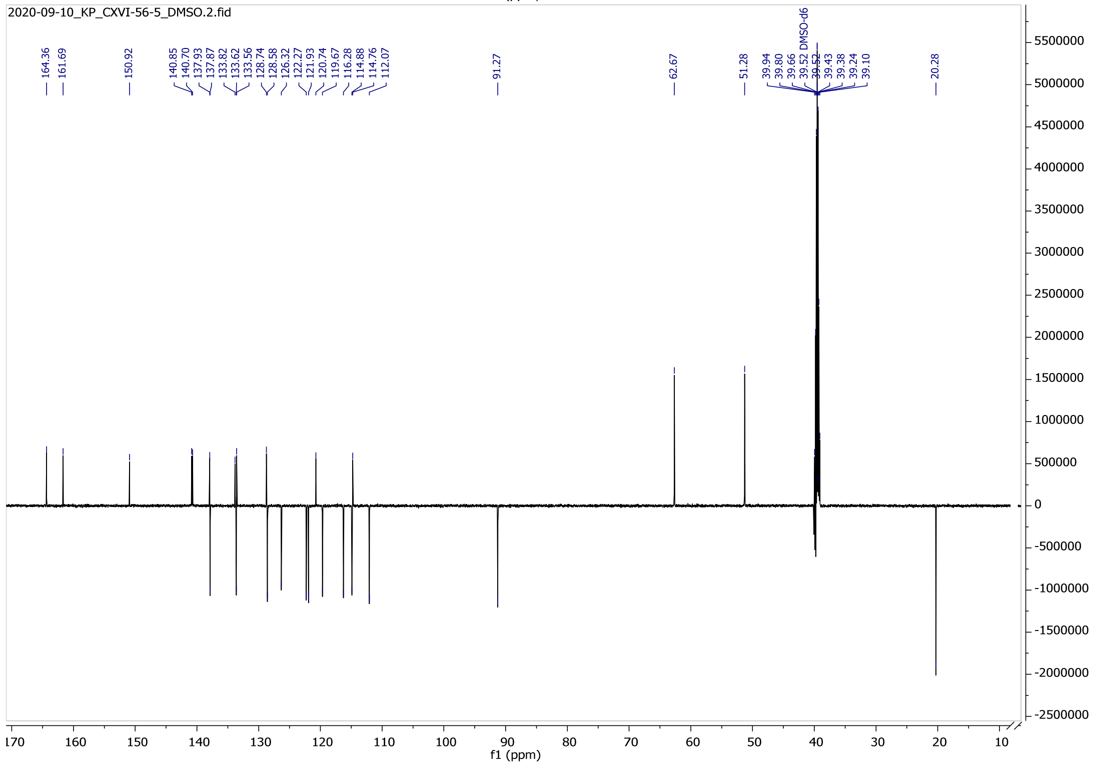
2020-09-10_KP_CXVI-56-5_DMSO.2.fid
f1 (ppm)

This image cannot currently be displayed.

This image cannot currently be displayed.

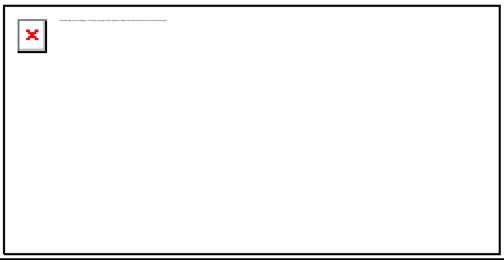

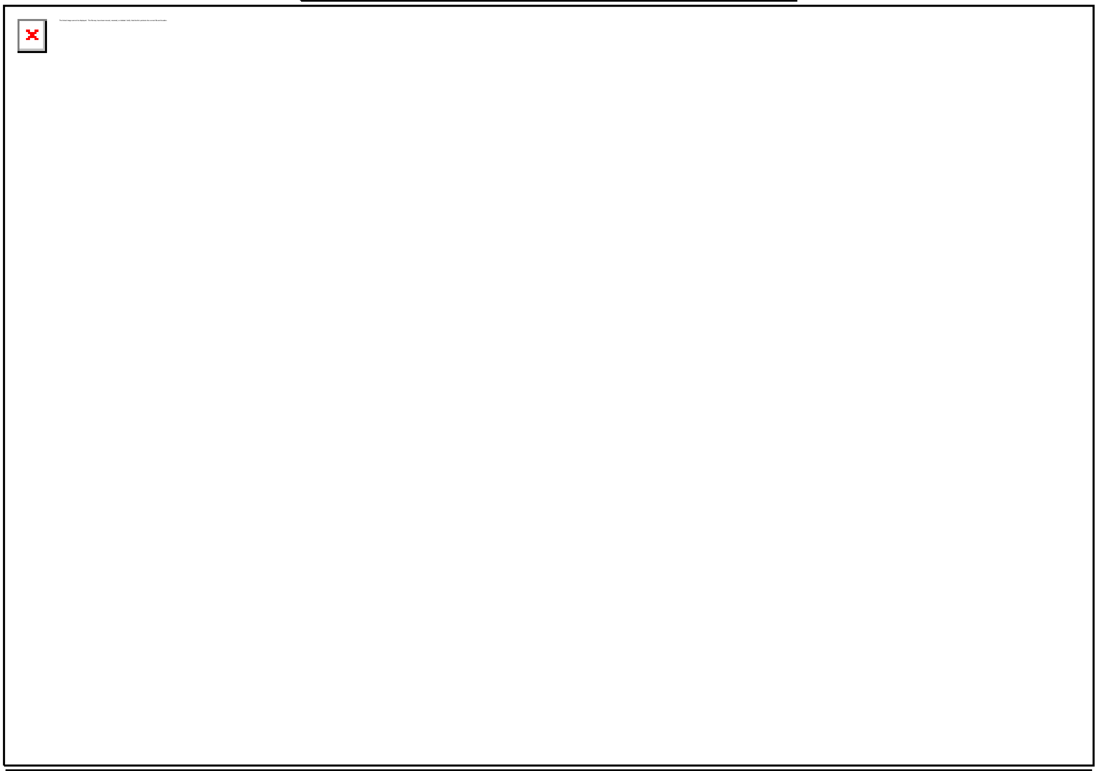

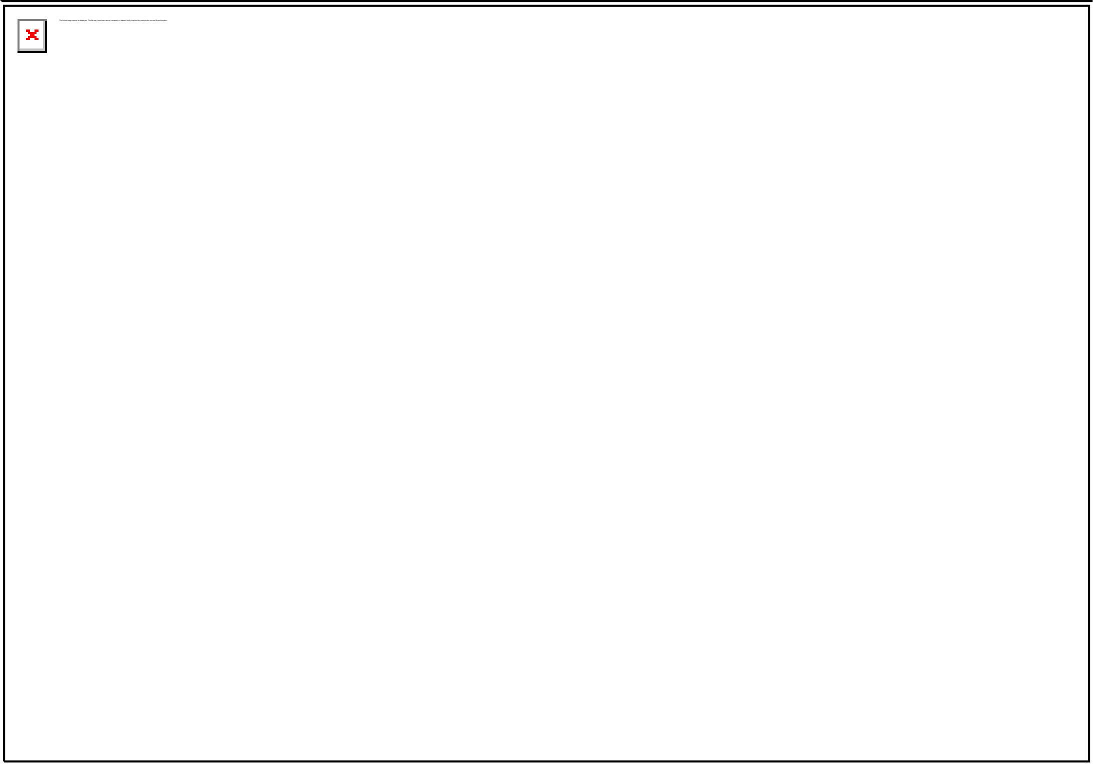

This image cannot currently be displayed.

This image cannot currently be displayed.

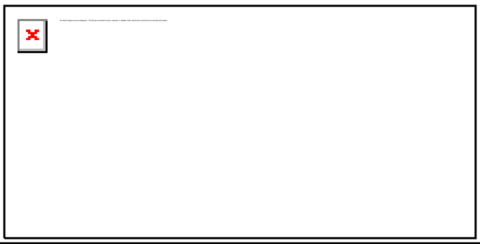

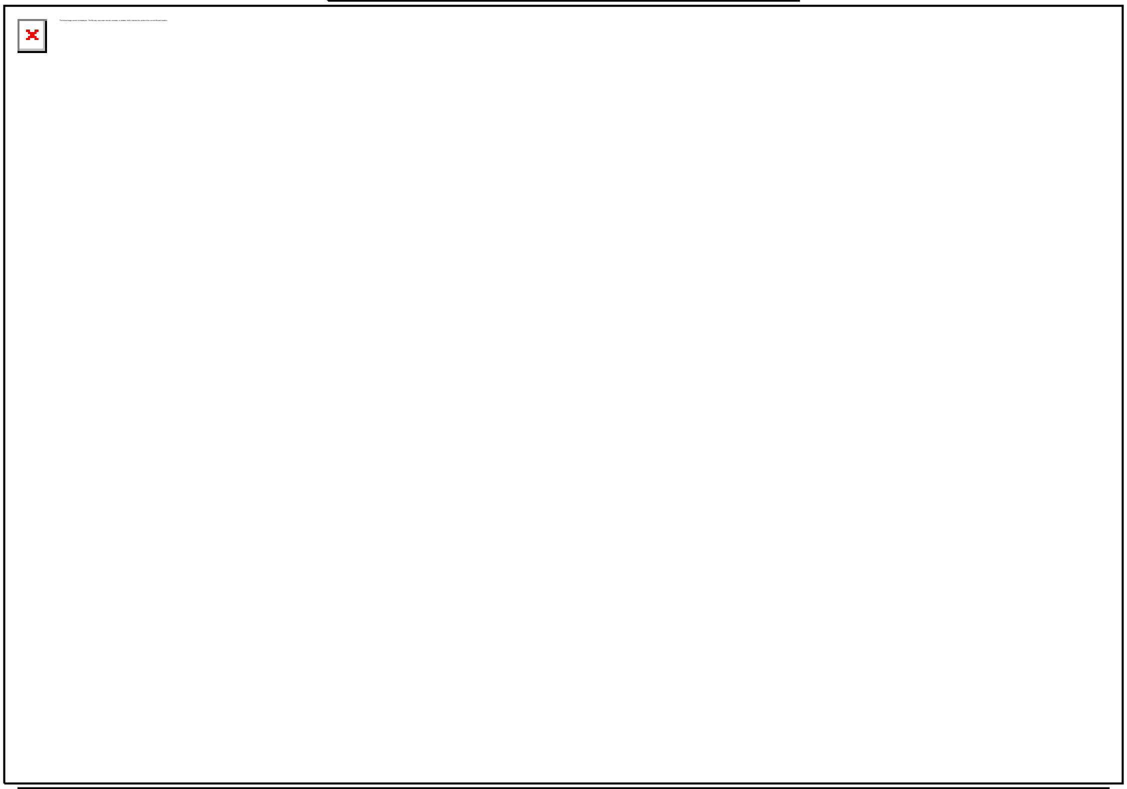

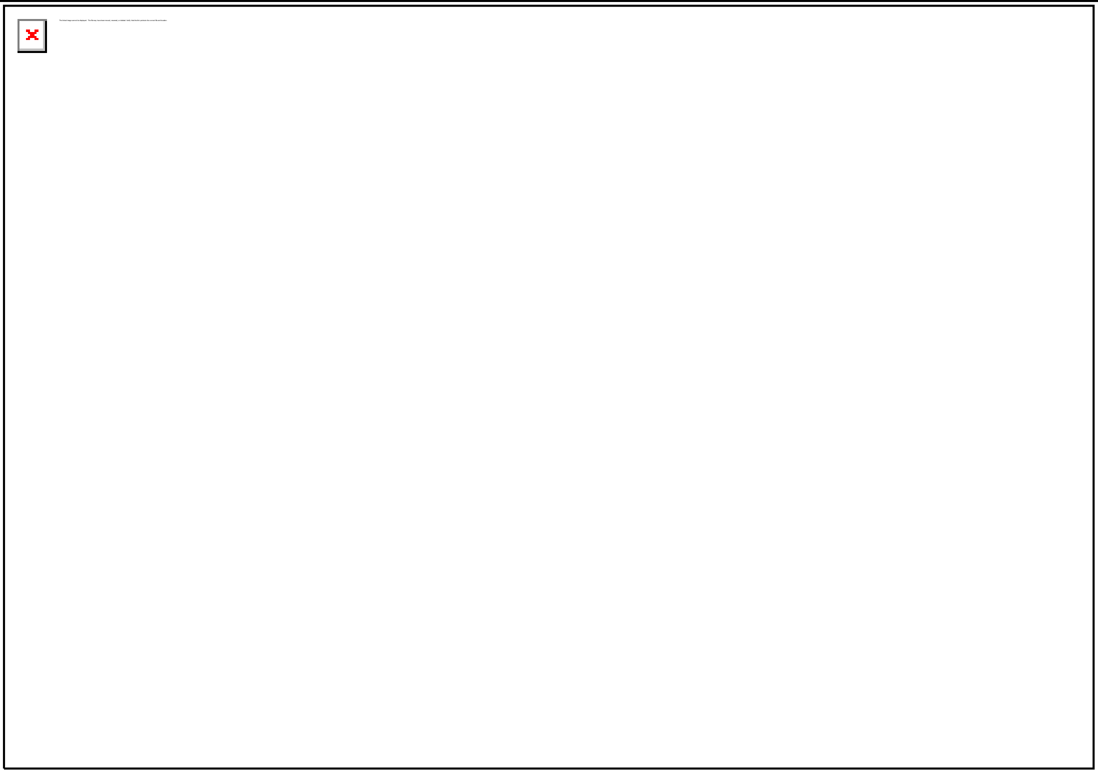

This image cannot currently be displayed.

This image cannot currently be displayed.

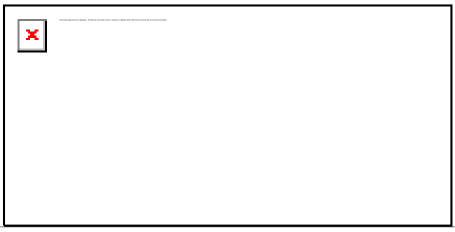

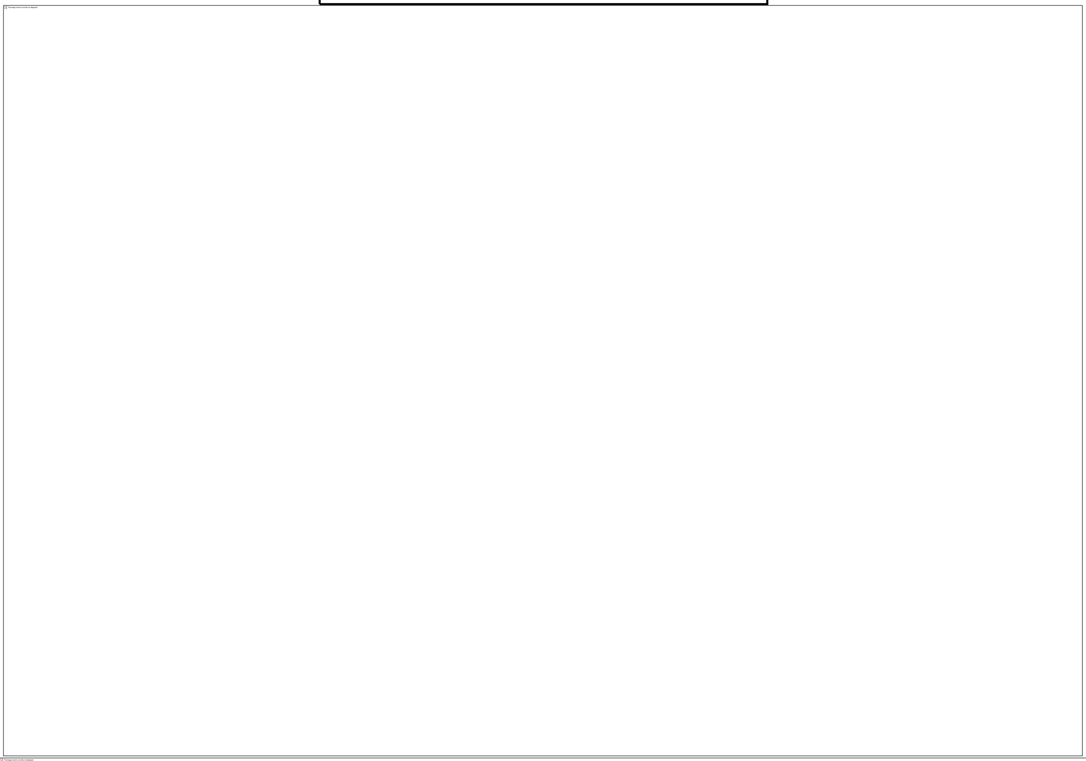

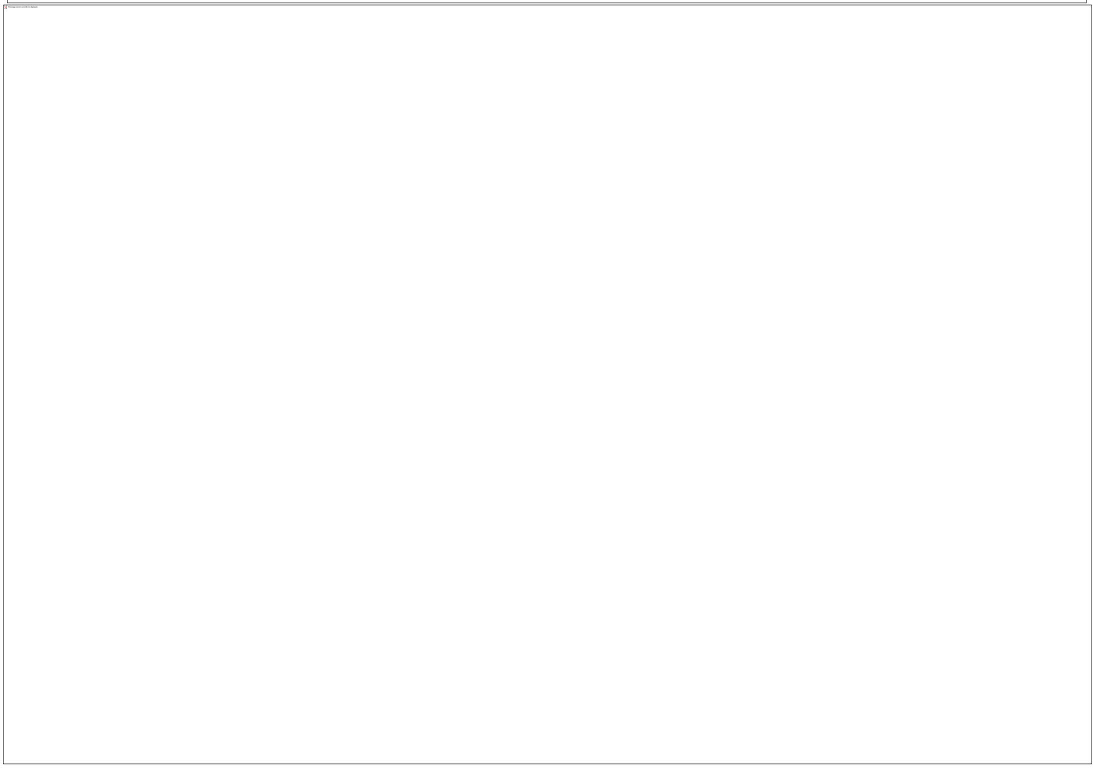

This image cannot currently be displayed.

This image cannot currently be displayed.

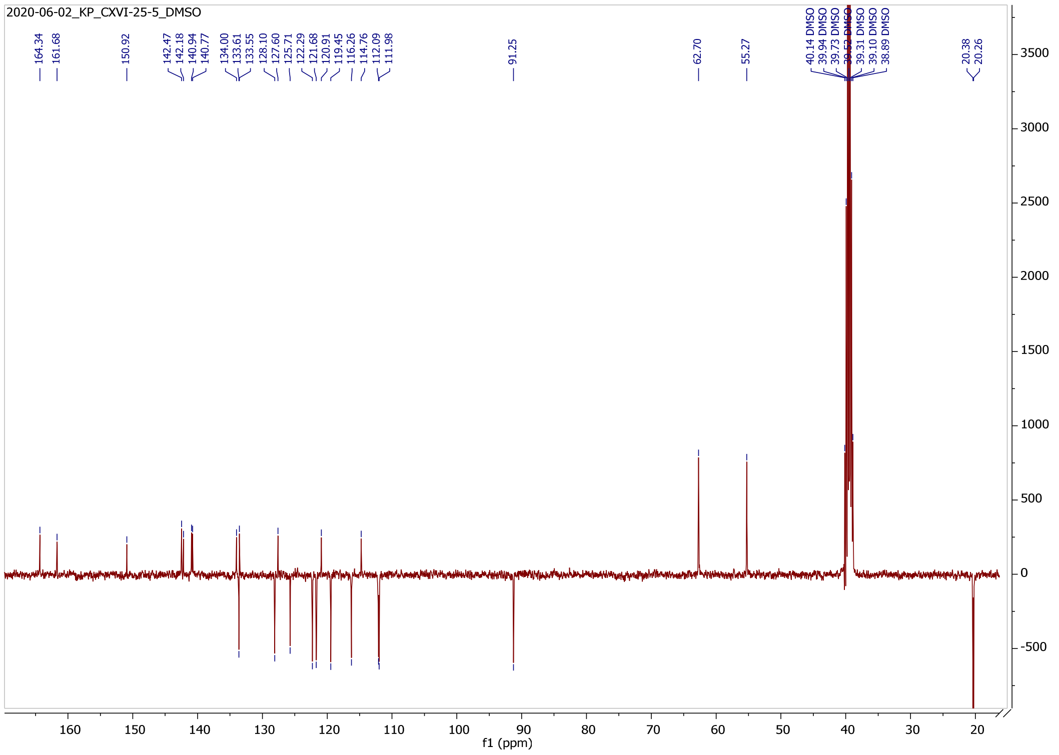
2020-06-02_KP_CXVI-25-5_DMSO
164.34
161.68
150.92
142.47
142.18
140.94
140.77
134.00
133.61
133.55
128.10
127.60
125.71
122.29
121.68
120.91
119.45
116.26
114.76
112.09
111.98
91.25
62.70
55.27
40.14 DMSO
39.94 DMSO
39.73 DMSO
39.31 DMSO
39.10 DMSO
38.89 DMSO
20.38
20.26
3500
3000
2500
2000
1500
1000
500
0
-500
160
150
140
130
120
110
100
90
80
70
60
50
40
30
20
f1 (ppm)

This image cannot currently be displayed.

This image cannot currently be displayed.

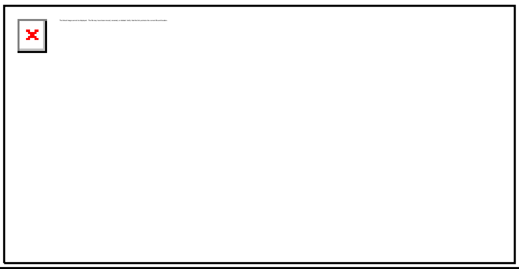

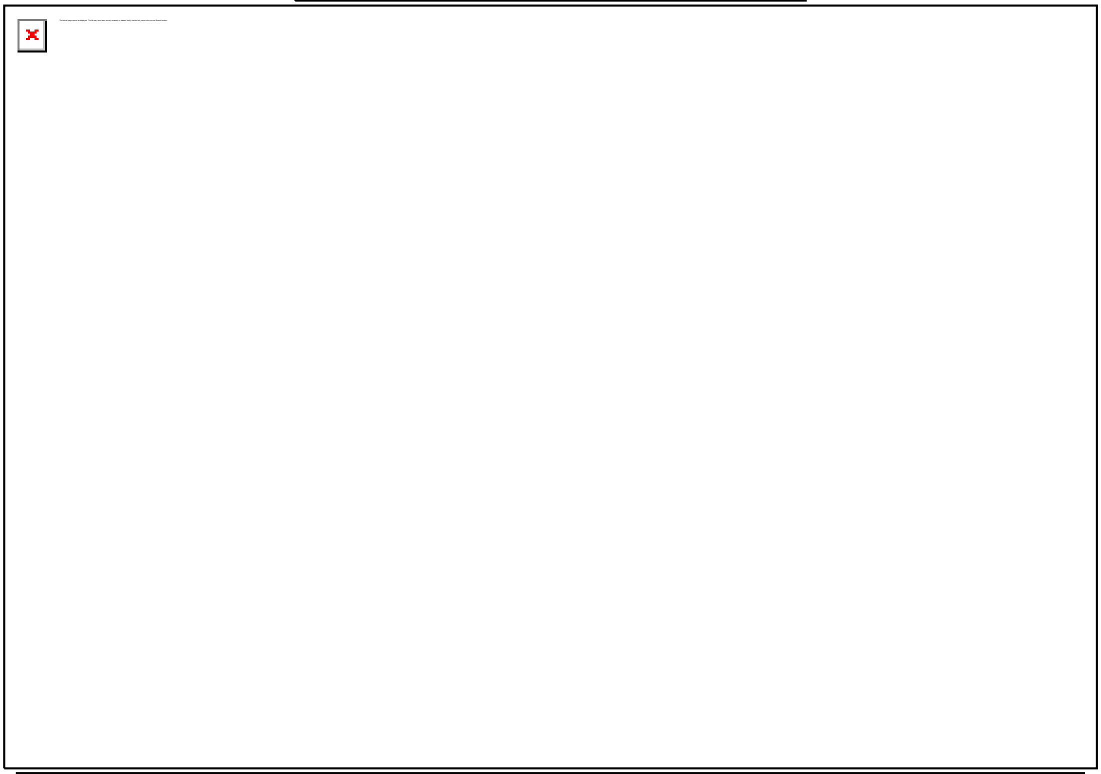

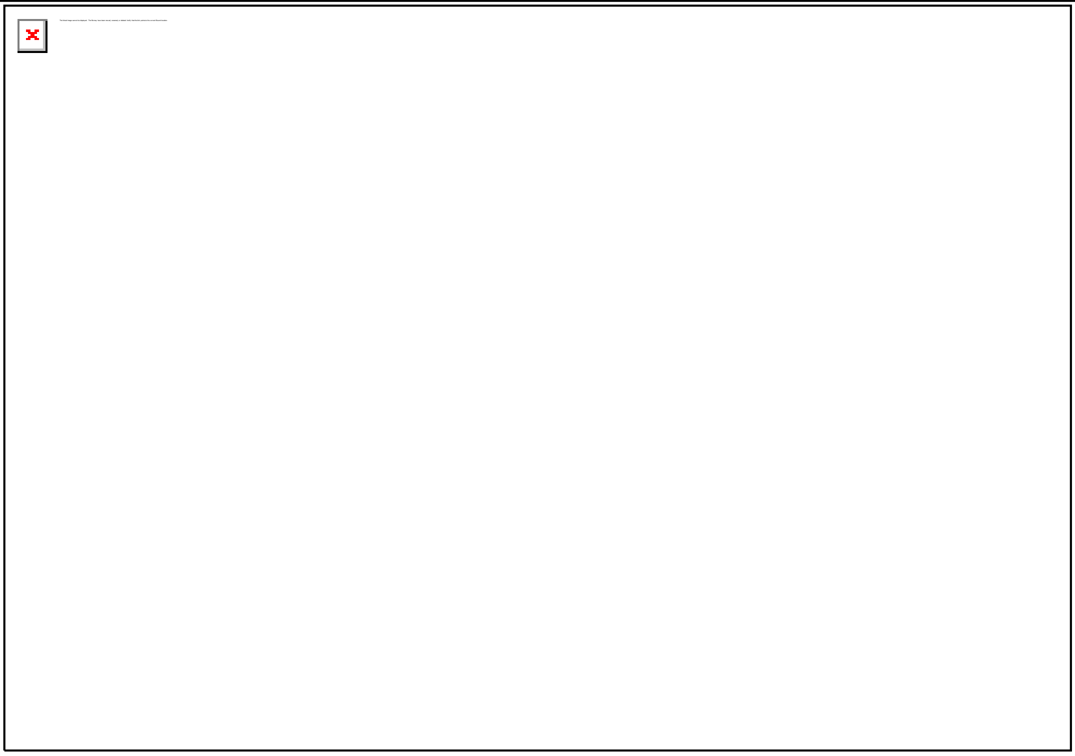

This image cannot currently be displayed.

This image cannot currently be displayed.

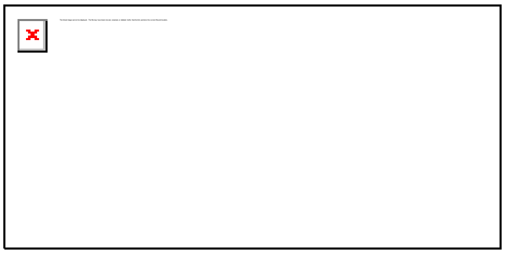

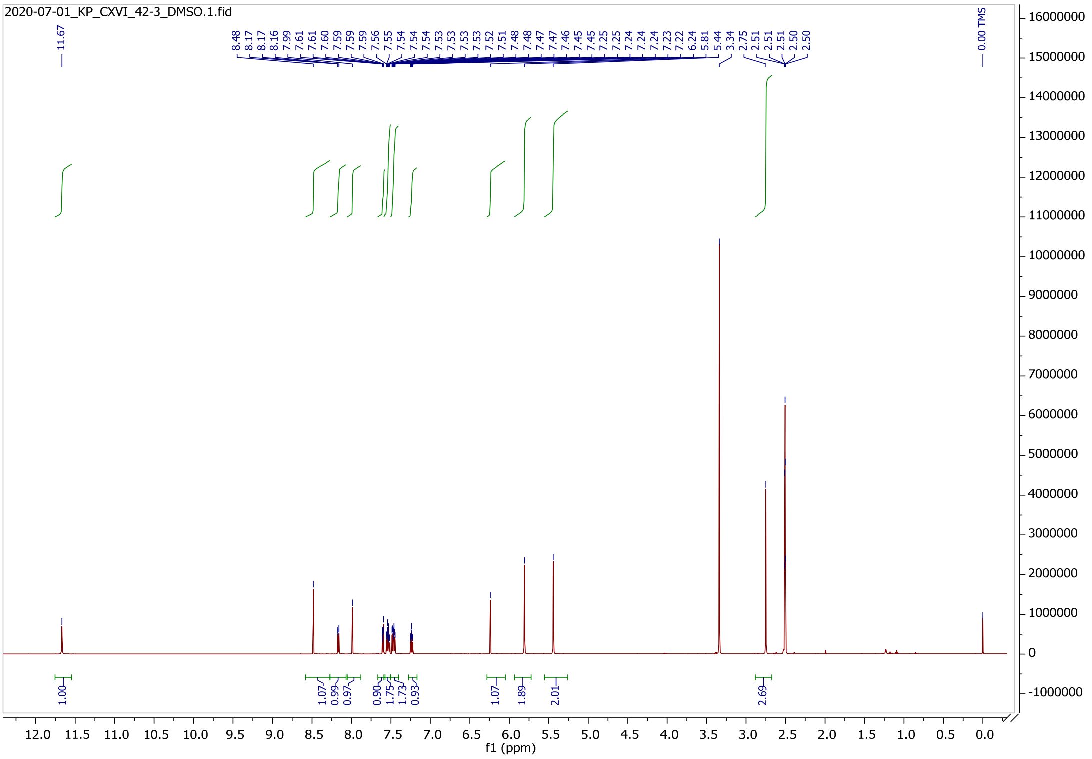

2020-07-01_KP_CXVI_42-3_DMSO.1.fid
11.67
8.48 8.17 8.17 8.16 7.99 7.61 7.61 7.60 7.59 7.59 7.59 7.56 7.55 7.54 7.54 7.54 7.54 7.53 7.53 7.53 7.53 7.52 7.51 7.48 7.48 7.47 7.46 7.45 7.45 7.25 7.25 7.24 7.24 7.23 7.22 6.24 5.81 5.44 3.34 2.75 2.51 2.51 2.51 2.50 2.50
0.00 TMS
1.00 1.07 0.99 0.97 0.90 1.75 1.73 0.93 1.07 1.89 2.01 2.69
f1 (ppm)

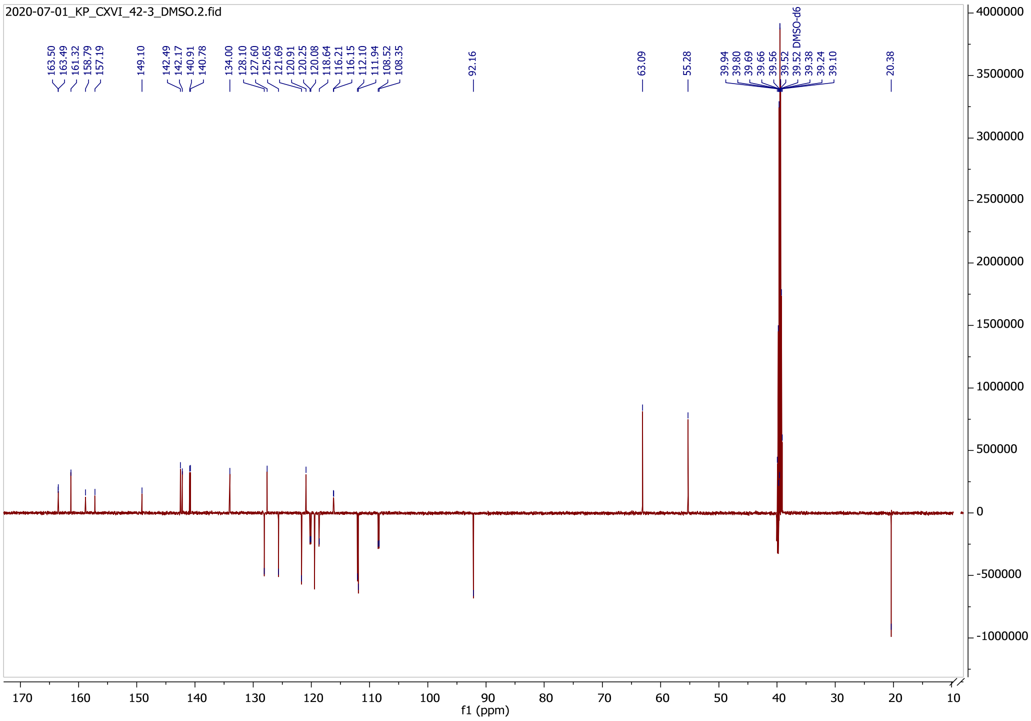

2020-07-01_KP_CXVI_42-3_DMSO.2.fid
163.50 163.49 161.32 158.79 157.19 149.10 142.49 142.17 140.91 140.78 134.00 128.10 127.60 125.65 121.69 120.91 120.25 120.08 118.64 116.21 116.15 112.10 111.94 108.52 108.35 92.16 63.09 55.28
39.94 39.80 39.69 39.66 39.56 39.52 39.52 DMSO-d6 39.38 39.24 39.10 20.38
f1 (ppm)

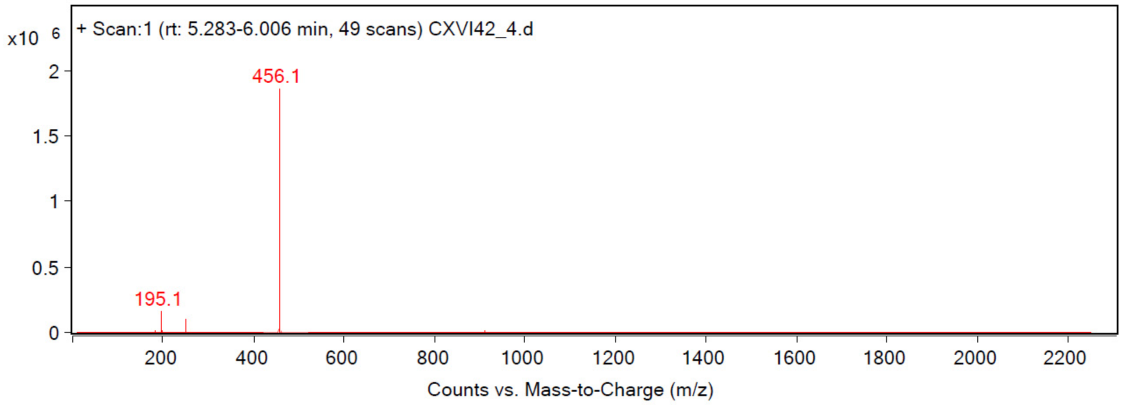
x10 6
+ Scan:1 (rt: 5.283-6.006 min, 49 scans) CXVI42_4.d
456.1
195.1
Counts vs. Mass-to-Charge (m/z)

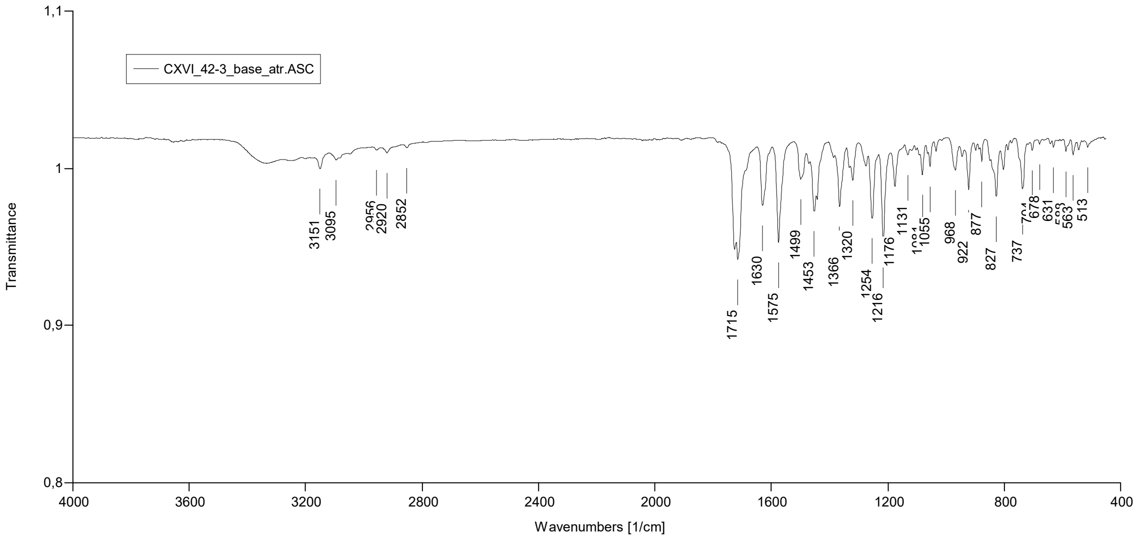
CXVI_42-3_base_atr.ASC
Transmittance
Wavenumbers [1/cm]
3151
3095
2920
2852
1715
1630
1575
1499
1453
1366
1320
1254
1216
1176
1131
1055
968
922
877
827
737
678
631
563
513

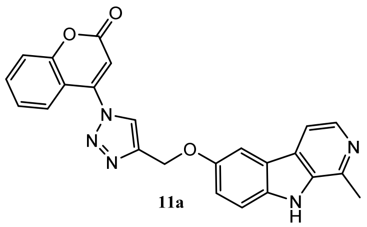
O
O
N
N
N
O
N
H
N

11a

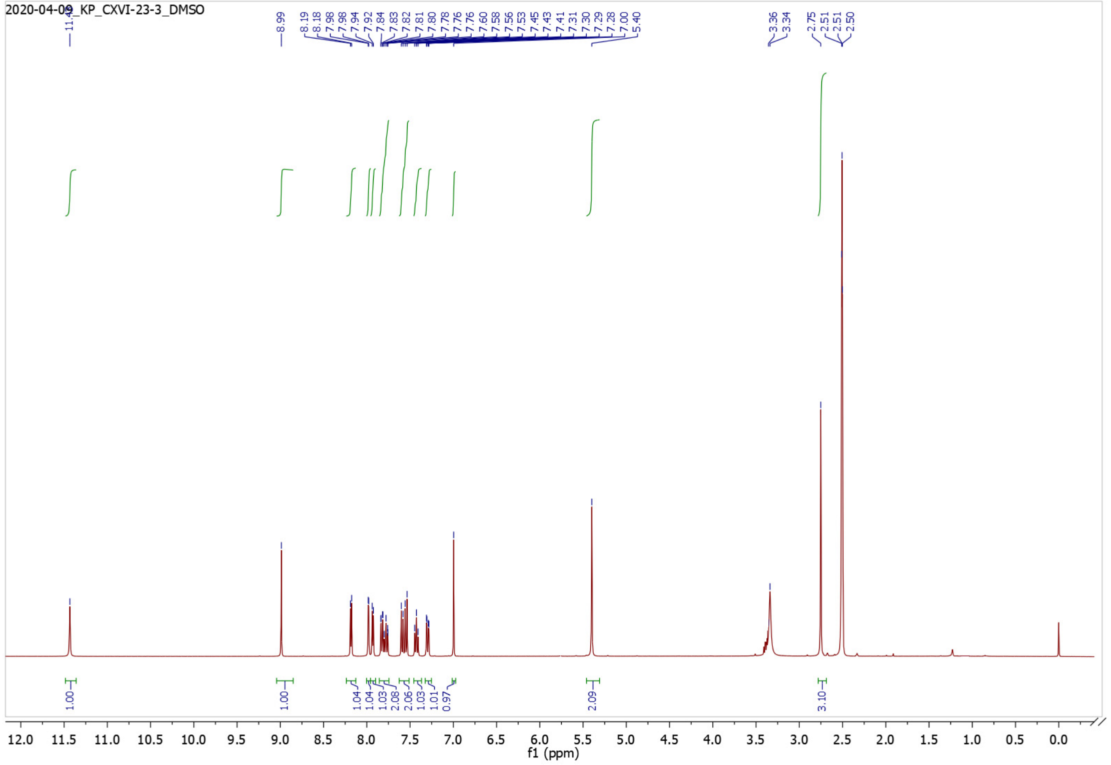
2020-04-09_KP_CXVI-23-3_DMSO
f1 (ppm)

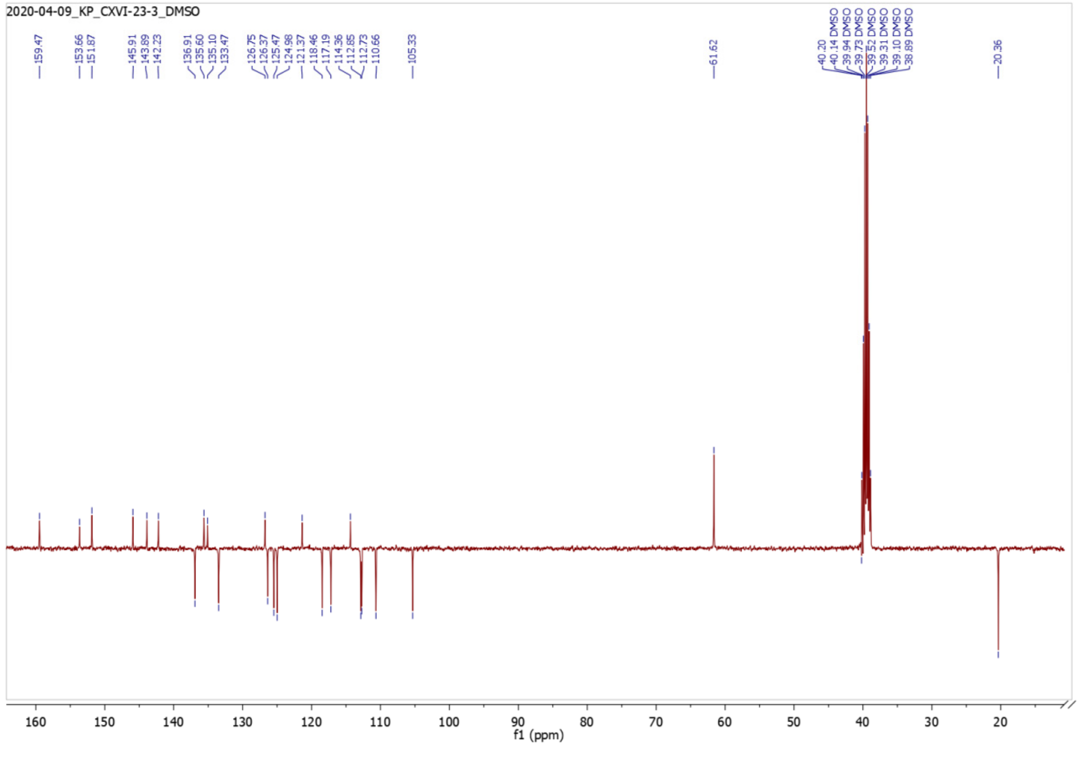
2020-04-09_KP_CXVI-23-3_DMSO
f1 (ppm)

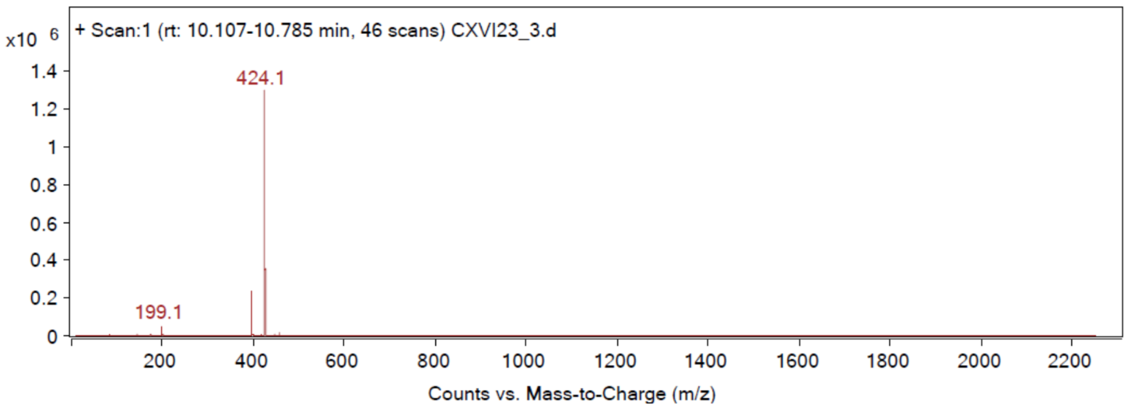
+ Scan:1 (rt: 10.107-10.785 min, 46 scans) CXVI23_3.d
x10 6
424.1
199.1
Counts vs. Mass-to-Charge (m/z)

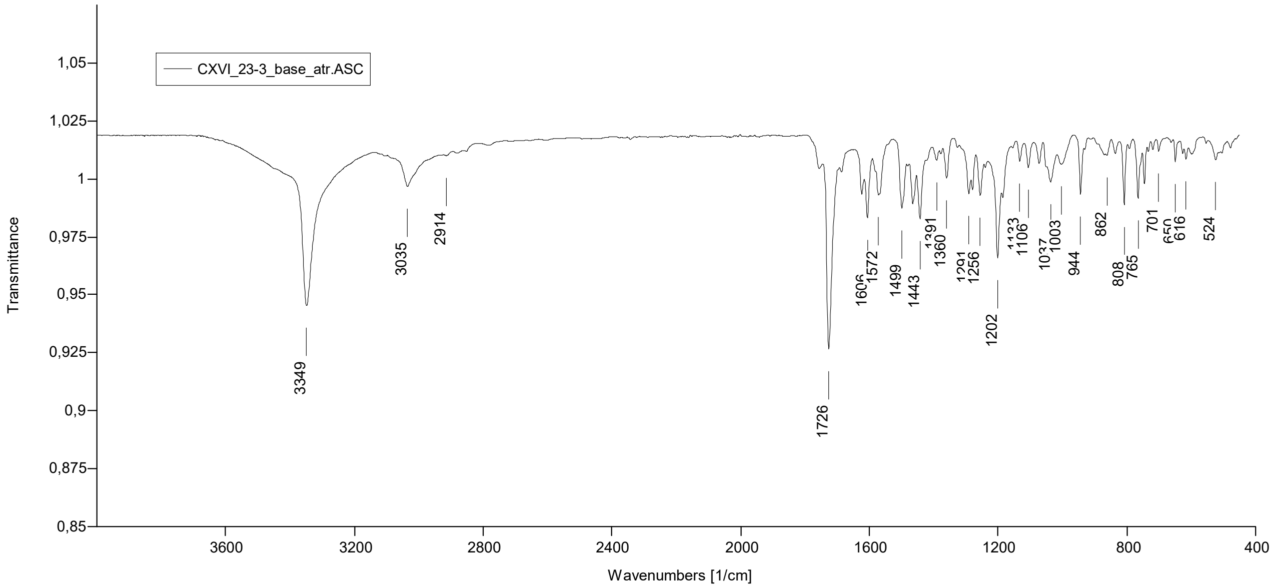
CXVI_23-3_base_atr.ASC
Transmittance
Wavenumbers [1/cm]
3349
3035
2914
1726
1572
1499
1443
1360
1256
1202
1106
1003
944
862
808
765
701
616
524

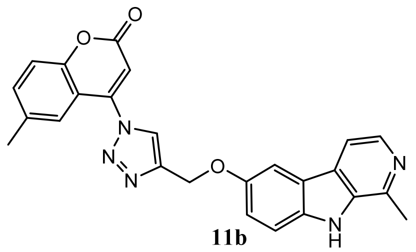

11b

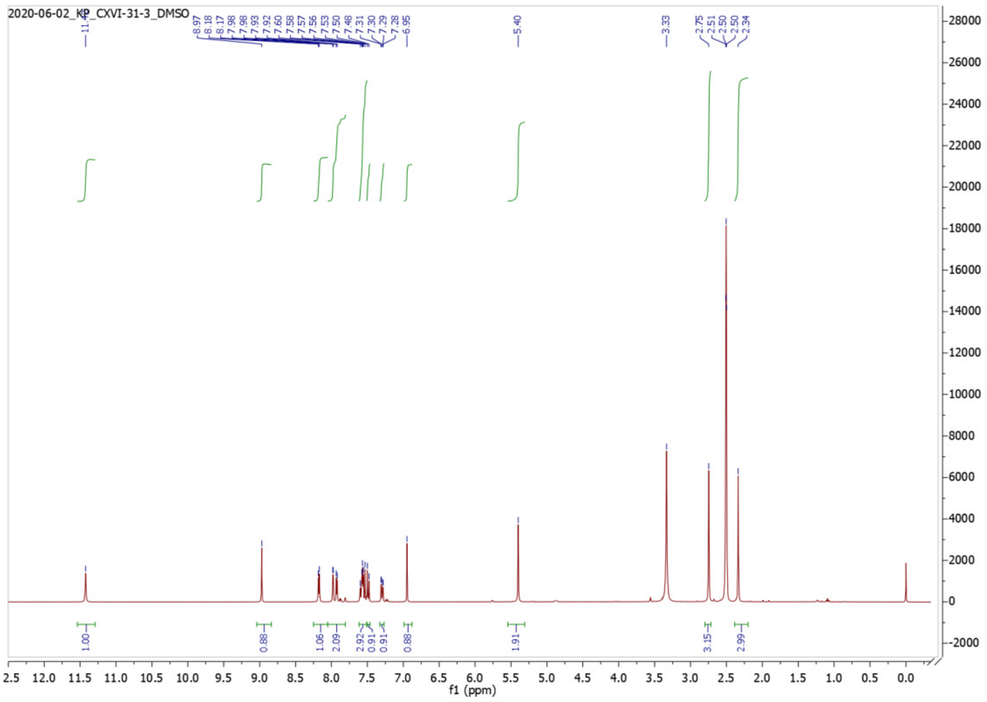
2020-06-02_KP_CXVI-31-3_DMSO
f1 (ppm)

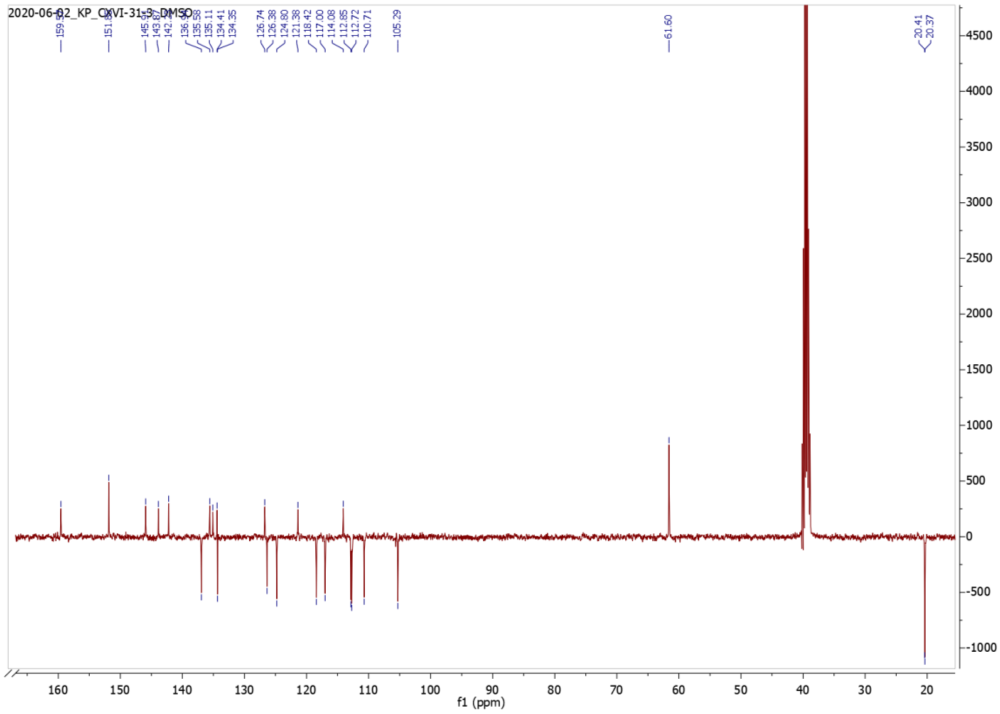
2020-06-02_KP_CXVI-31-3_DMSO
f1 (ppm)

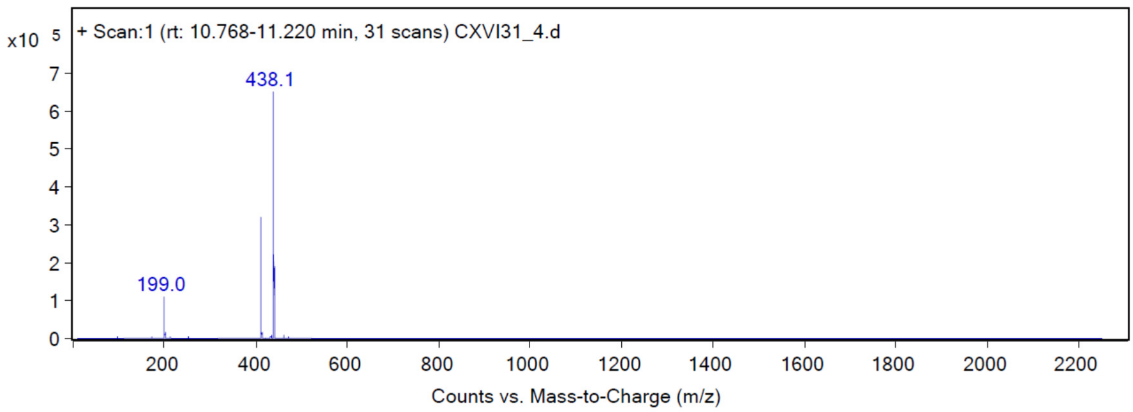
x10 5
+ Scan:1 (rt: 10.768-11.220 min, 31 scans) CXVI31_4.d
438.1
199.0
Counts vs. Mass-to-Charge (m/z)

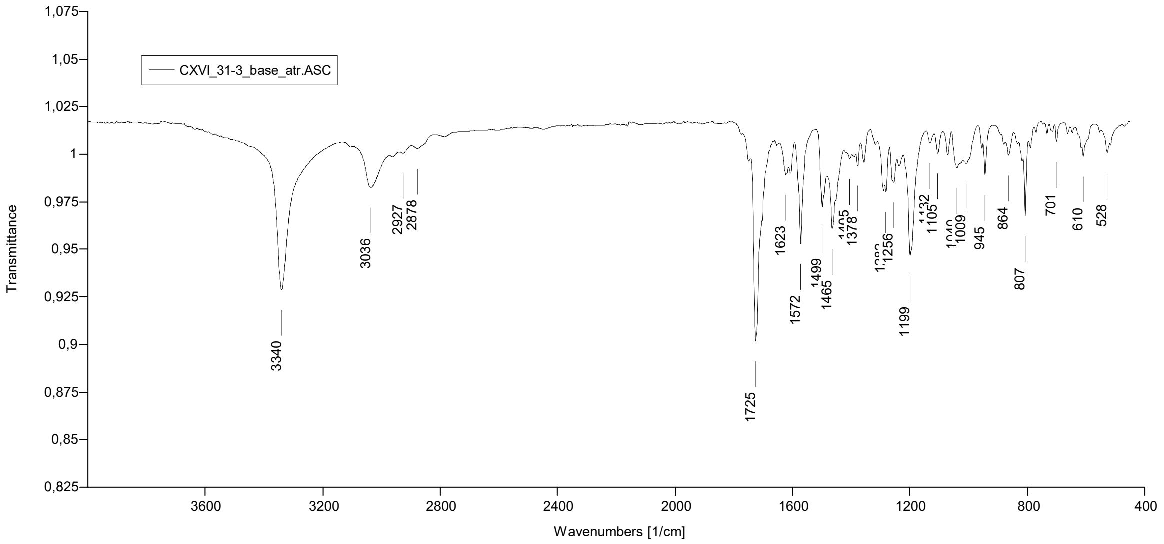
CXVI_31-3_base_atr.ASC
Transmittance
Wavenumbers [1/cm]
3340
3036
2927
2878
1725
1623
1572
1499
1465
1378
1256
1199
1132
1105
1009
945
864
807
701
610
528

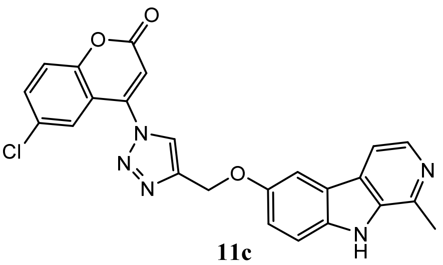

11c

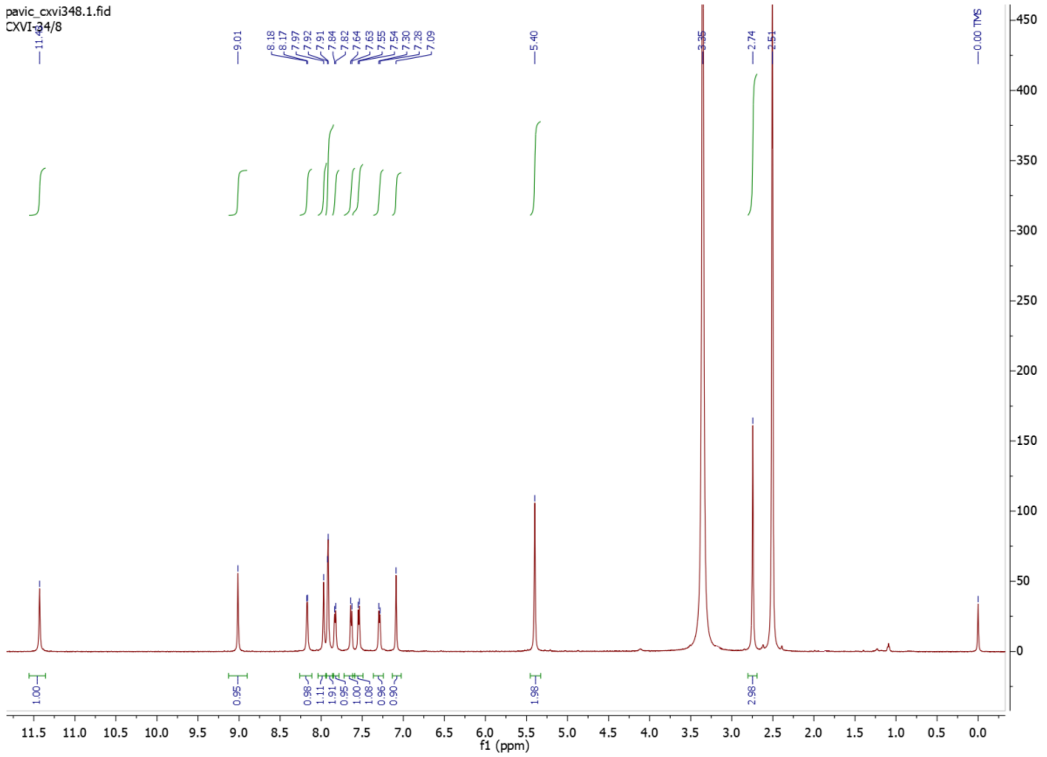
pavic_cxvi348.1.fid
CXVI-34/8
f1 (ppm)

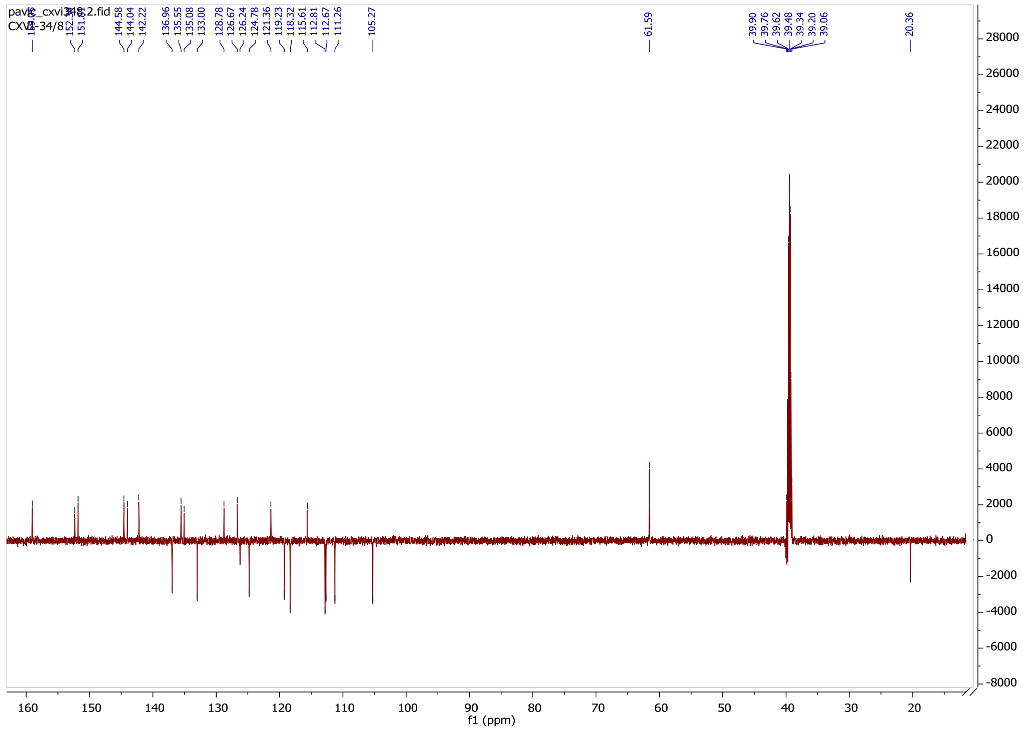
pavic_cxvi348.2.fid
CXVI-34/8
f1 (ppm)

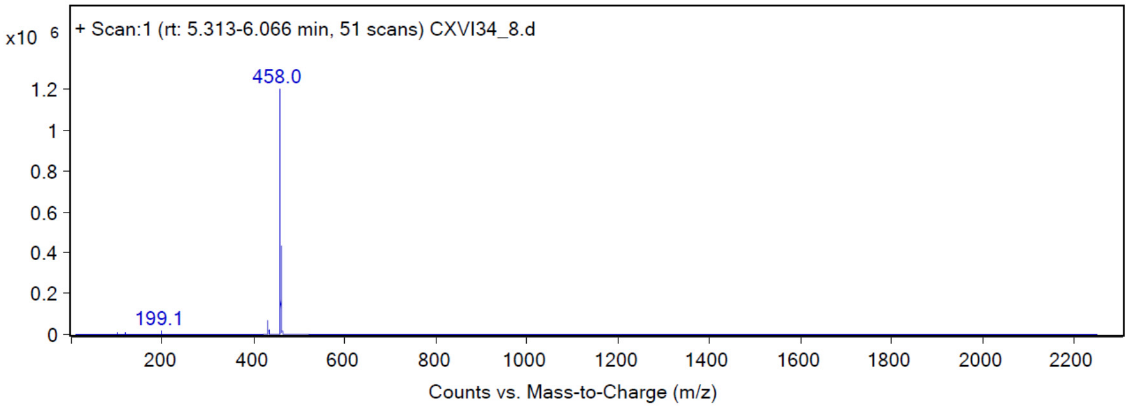
+ Scan:1 (rt: 5.313-6.066 min, 51 scans) CXVI34_8.d
x10 6
458.0
199.1
Counts vs. Mass-to-Charge (m/z)

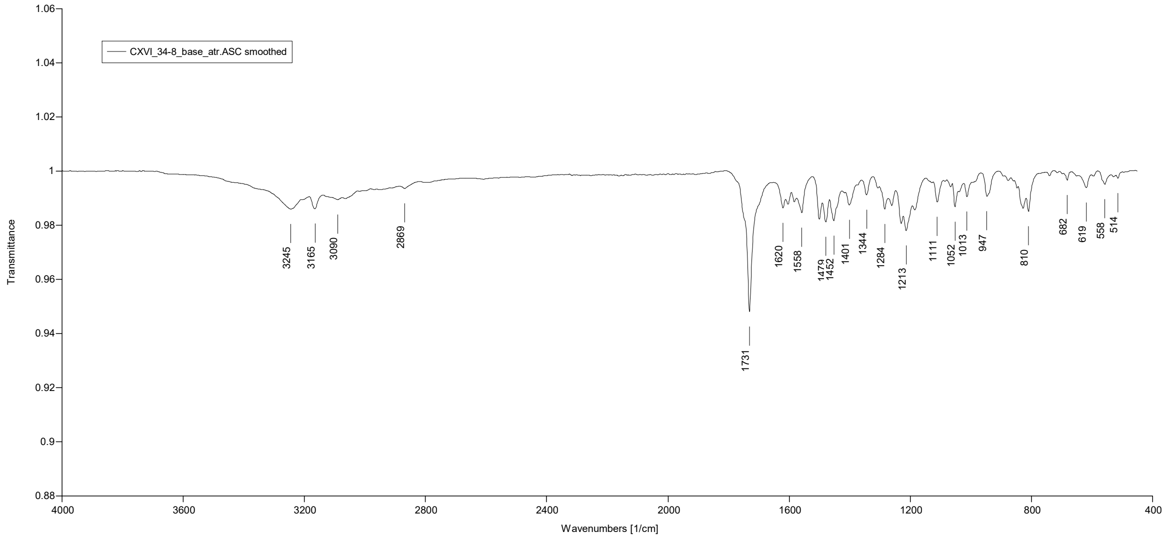
CXVI_34-8_base_atr.ASC smoothed
Transmittance
Wavenumbers [1/cm]
3245
3165
3090
2869
1731
1620
1558
1479
1452
1401
1344
1284
1213
1111
1052
1013
947
810
682
619
558
514

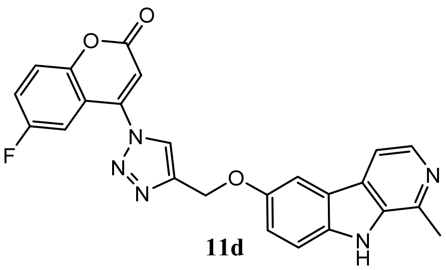

11d

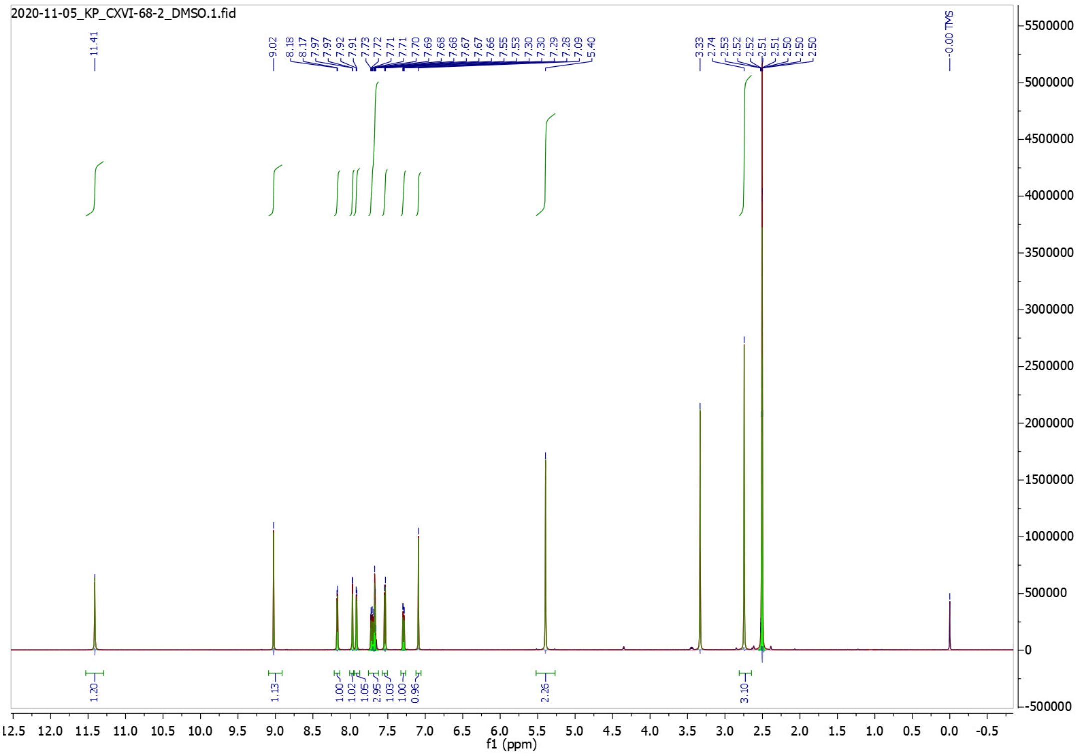
2020-11-05_KP_CXVI-68-2_DMSO.1.fid
11.41
9.02 8.18 8.17 7.97 7.97 7.92 7.91 7.73 7.72 7.71 7.71 7.70 7.69 7.68 7.68 7.67 7.67 7.66 7.55 7.53 7.30 7.30 7.29 7.28 7.09 5.40
3.33 2.74 2.53 2.52 2.52 2.51 2.51 2.50 2.50 2.50
0.00 TMS
1.20
1.13
1.00 1.02 1.05 2.95 1.03 1.00 0.96
2.26
3.10
f1 (ppm)

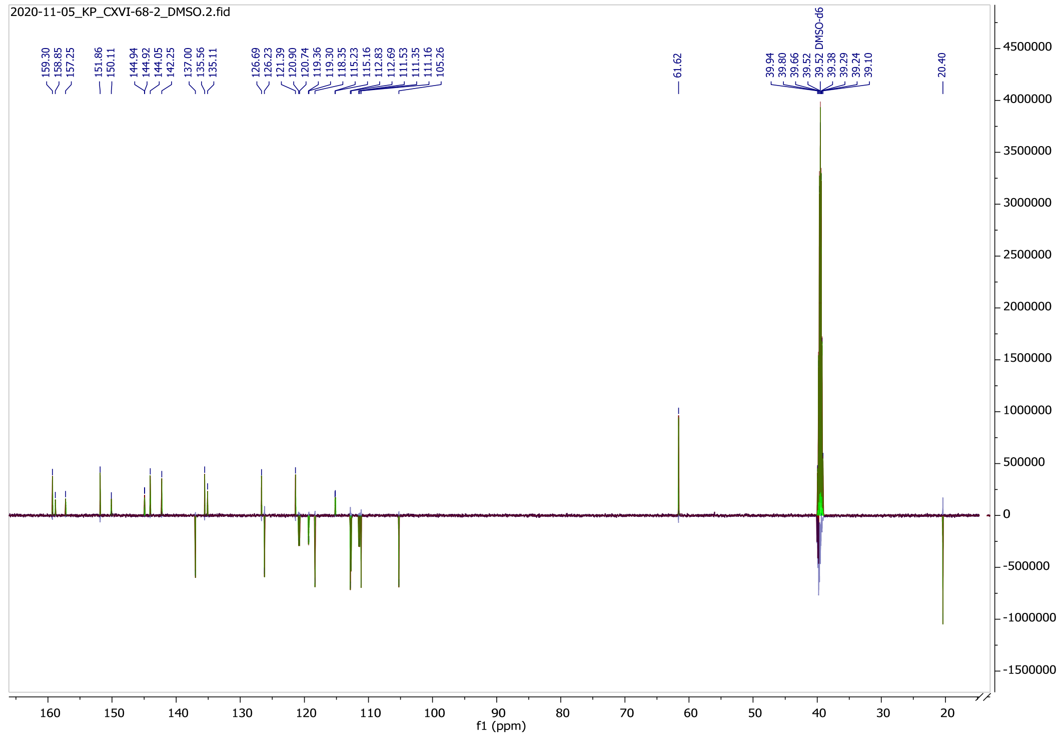
2020-11-05_KP_CXVI-68-2_DMSO.2.fid
159.30 158.85 157.25
151.86 150.11
144.94 144.92 144.05 142.25
137.00 135.56 135.11
126.69 126.23 121.39 120.90 120.74 119.36 119.30 118.35 115.23 115.16 112.83 112.69 111.53 111.35 111.16 105.26
61.62
39.94 39.80 39.66 39.52 39.52 DMSO-d6 39.38 39.29 39.24 39.10
20.40
f1 (ppm)

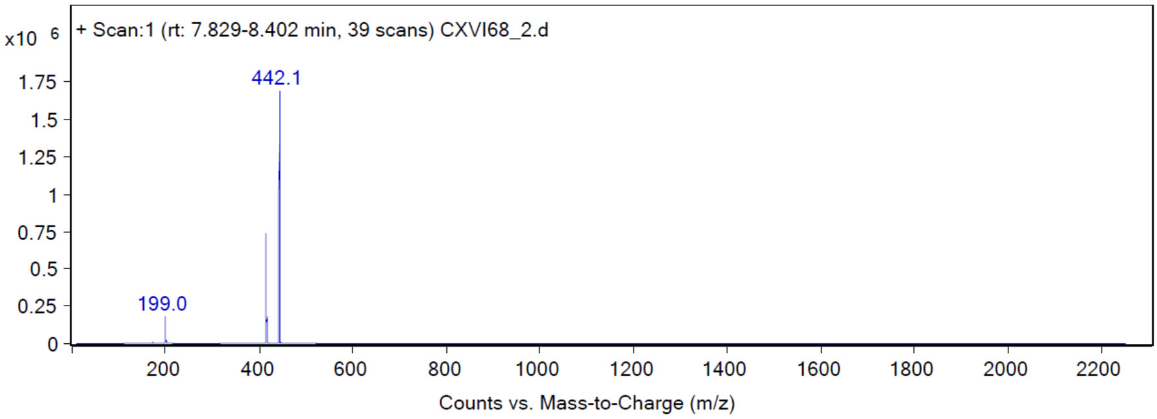
x10 6
+ Scan:1 (rt: 7.829-8.402 min, 39 scans) CXVI68_2.d
442.1
199.0
Counts vs. Mass-to-Charge (m/z)

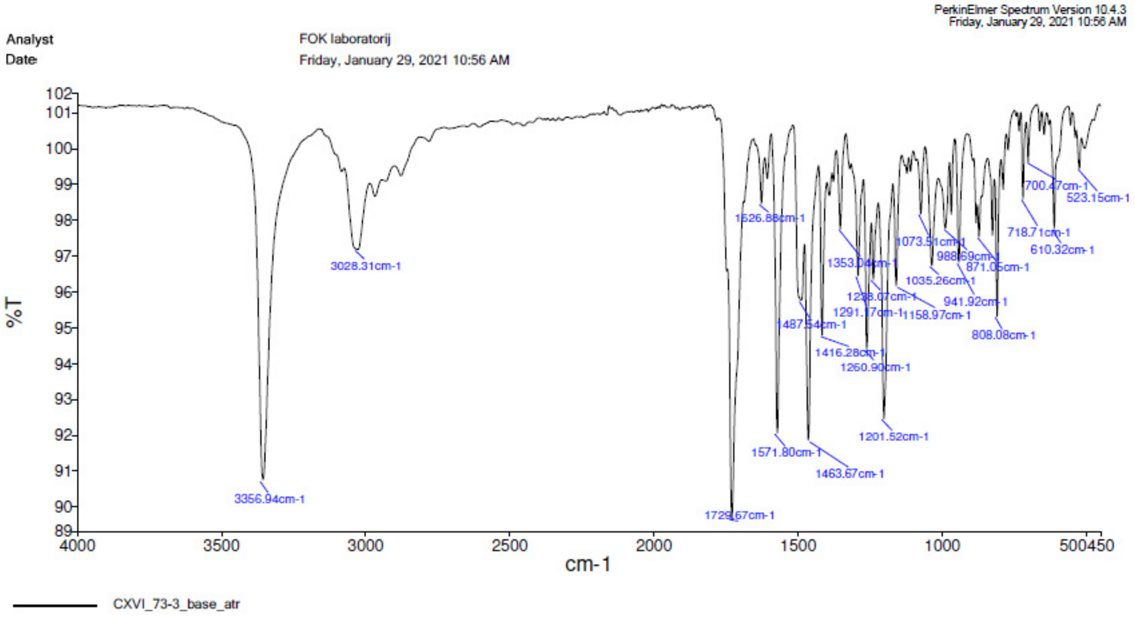
PerkinElmer Spectrum Version 10.4.3
Friday, January 29, 2021 10:56 AM
Analyst
Date
FOK laboratorij
Friday, January 29, 2021 10:56 AM
%T
cm-1
3356.94cm-1
3028.31cm-1
1729.67cm-1
1626.88cm-1
1571.80cm-1
1487.54cm-1
1463.67cm-1
1416.28cm-1
1353.04cm-1
1291.17cm-1
1260.90cm-1
1238.07cm-1
1201.52cm-1
1158.97cm-1
1073.51cm-1
1035.26cm-1
988.69cm-1
941.92cm-1
871.05cm-1
808.08cm-1
718.71cm-1
700.47cm-1
610.32cm-1
523.15cm-1
CXVI_73-3_base_atr

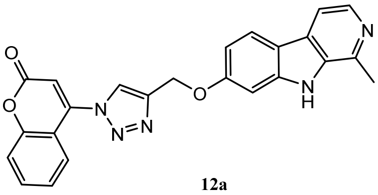

12a

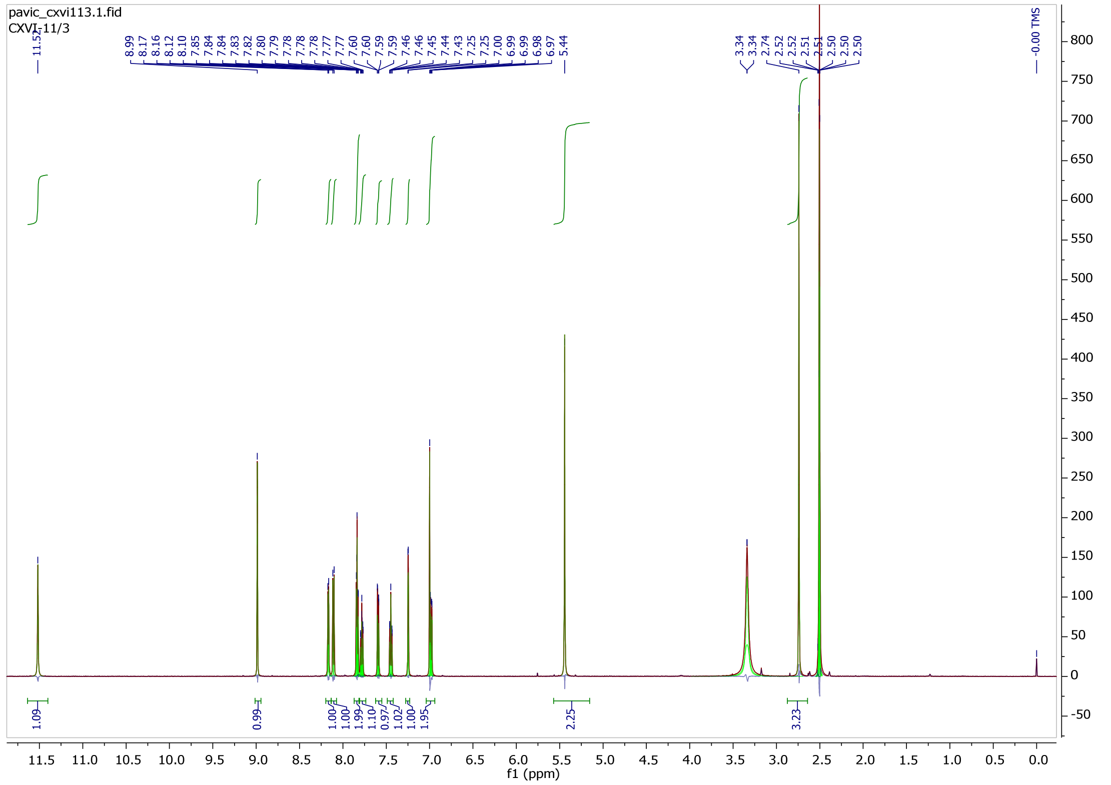

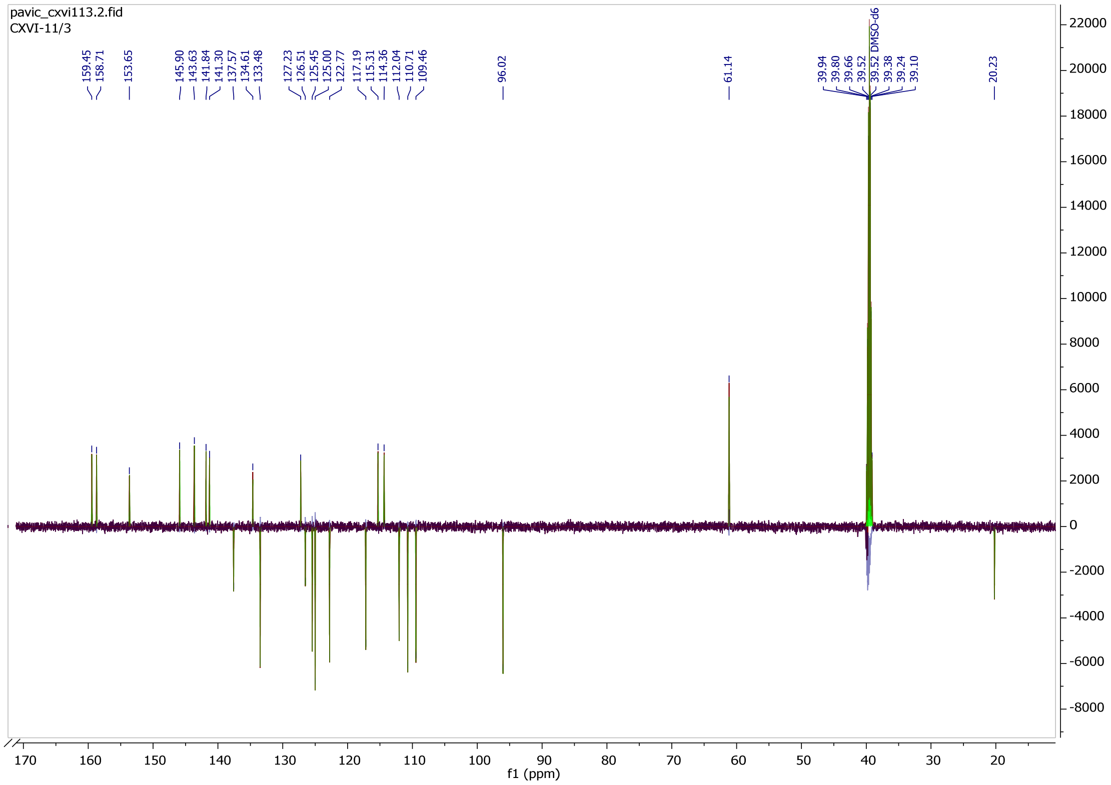

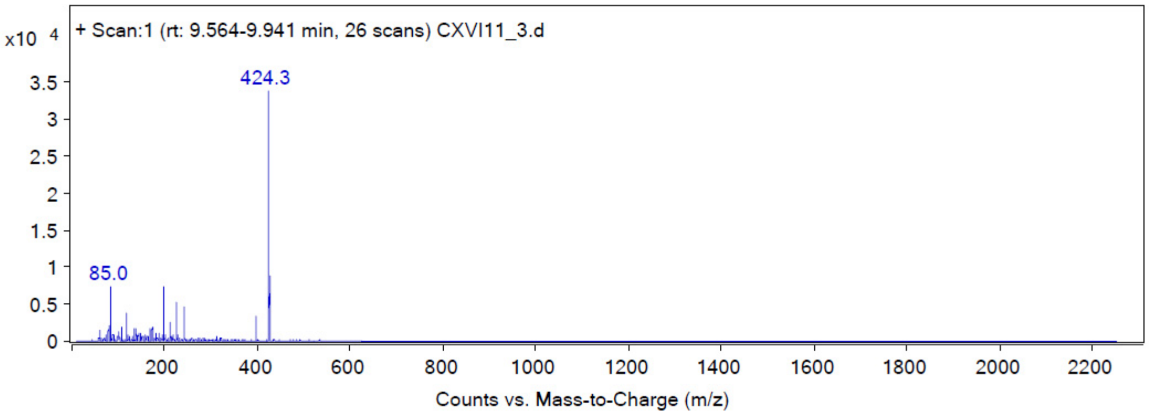
+ Scan:1 (rt: 9.564-9.941 min, 26 scans) CXVI11_3.d
x10 4
424.3
85.0
Counts vs. Mass-to-Charge (m/z)

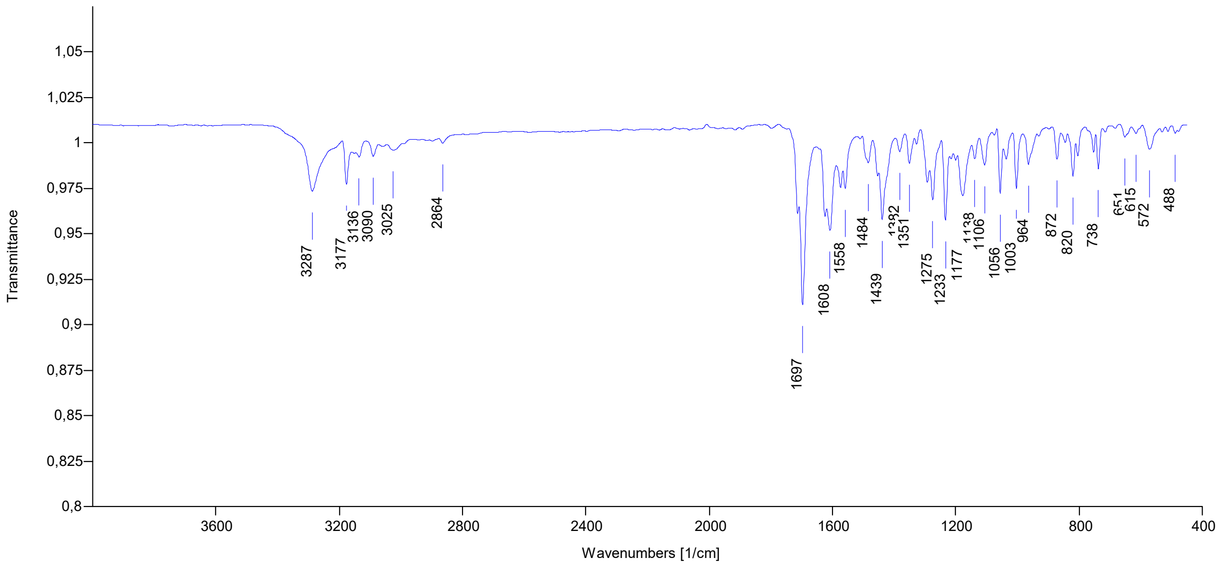
Transmittance
Wavenumbers [1/cm]
3287
3177
3136
3090
3025
2864
1697
1608
1558
1484
1439
1351
1275
1233
1177
1138
1106
1056
1003
964
872
820
738
651
615
572
488

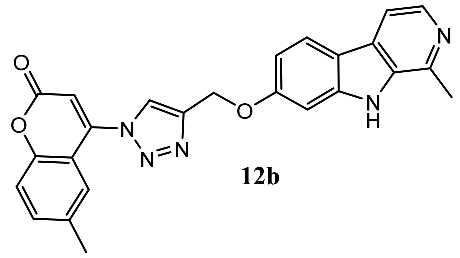
O
O
N
N
N
O
N
N
H

12b

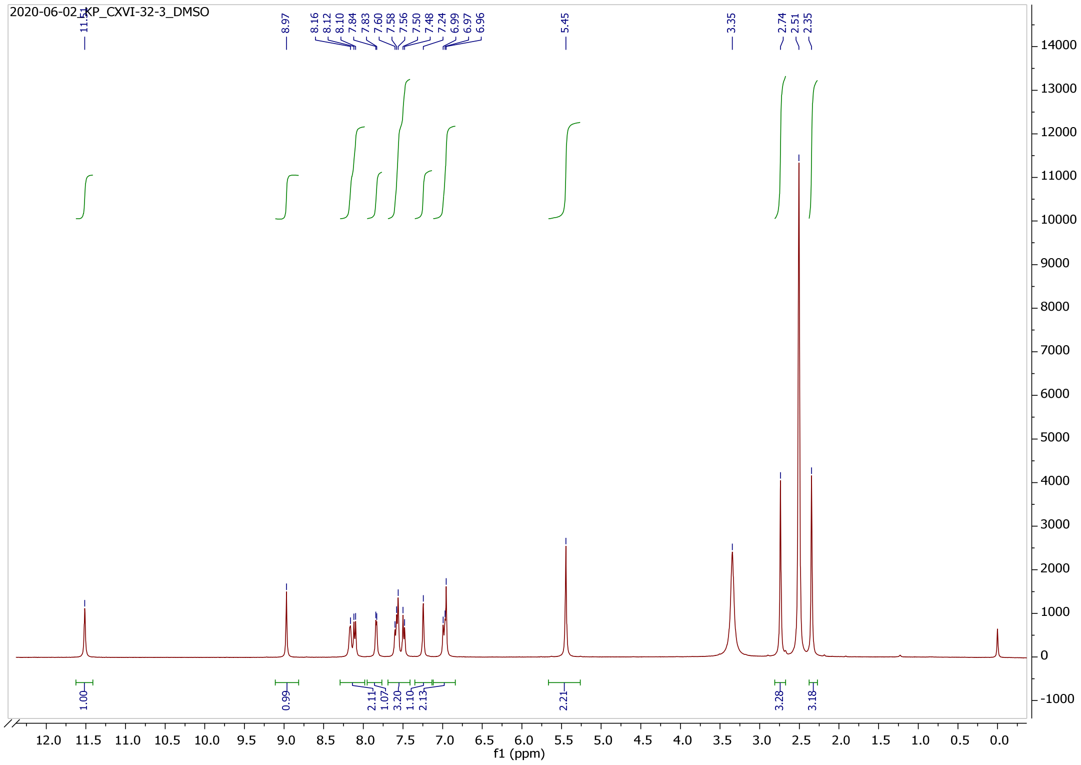
2020-06-02_KP_CXVI-32-3_DMSO
11.51
8.97
8.16
8.12
8.10
7.84
7.83
7.60
7.58
7.56
7.50
7.48
7.24
6.99
6.97
6.96
5.45
3.35
2.74
2.51
2.35
1.00
0.99
2.11
1.07
3.20
1.10
2.13
2.21
3.28
3.18
f1 (ppm)

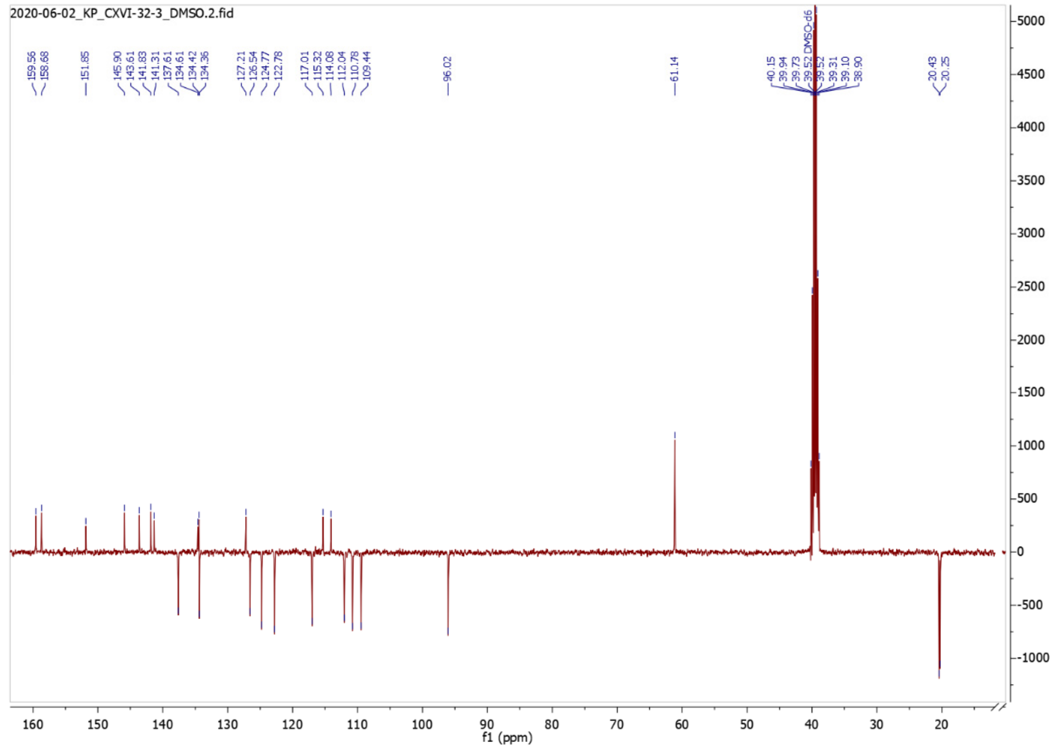
2020-06-02_KP_CXVI-32-3_DMSO.2.fid
159.56
158.68
151.85
145.90
143.61
141.83
141.31
137.61
134.61
134.42
134.36
127.21
126.54
124.77
122.78
117.01
115.32
114.08
112.04
110.78
109.44
96.02
61.14
40.15
39.94
39.73
39.52 DMSO-d6
39.52
39.31
39.10
38.90
20.43
20.25
f1 (ppm)

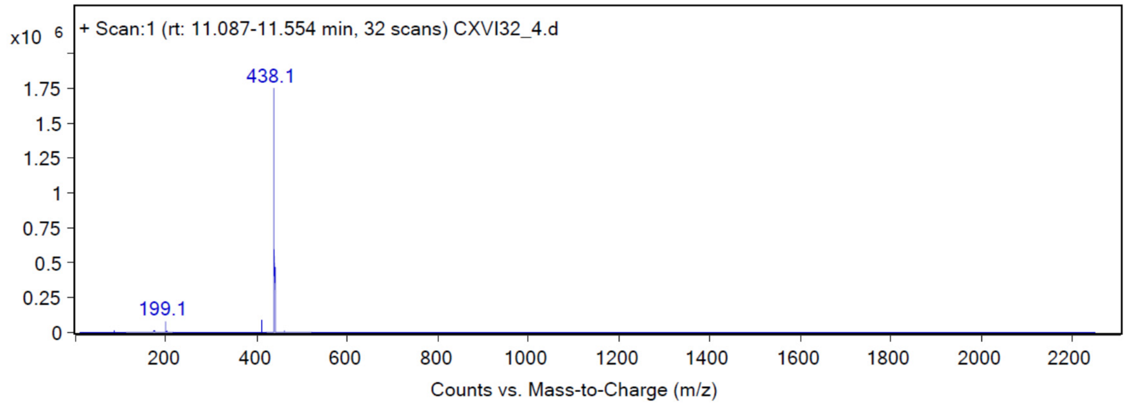
x10 6
+ Scan:1 (rt: 11.087-11.554 min, 32 scans) CXVI32_4.d
438.1
199.1
Counts vs. Mass-to-Charge (m/z)

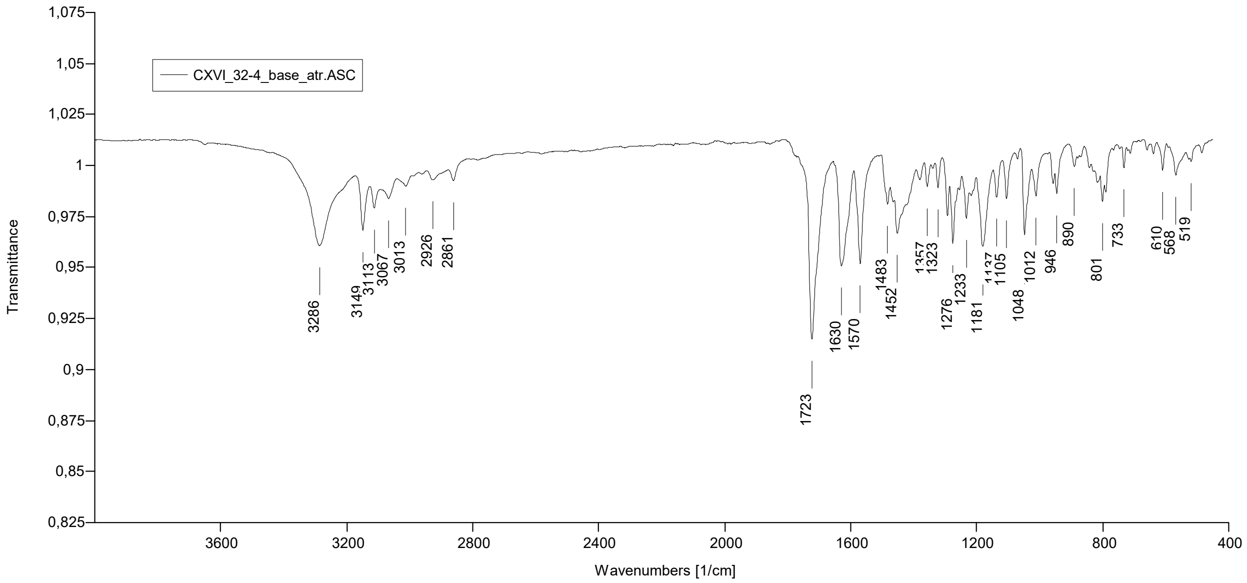
CXVI_32-4_base_atr.ASC
Transmittance
Wavenumbers [1/cm]
3286
3149
3113
3067
3013
2926
2861
1723
1630
1570
1483
1452
1357
1323
1276
1233
1181
1127
1105
1048
1012
946
890
801
733
610
568
519

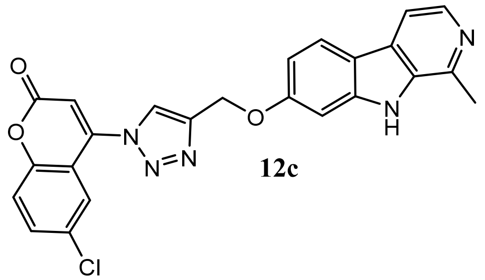
O
O
N
N
N
O
N
N
H
Cl

12c

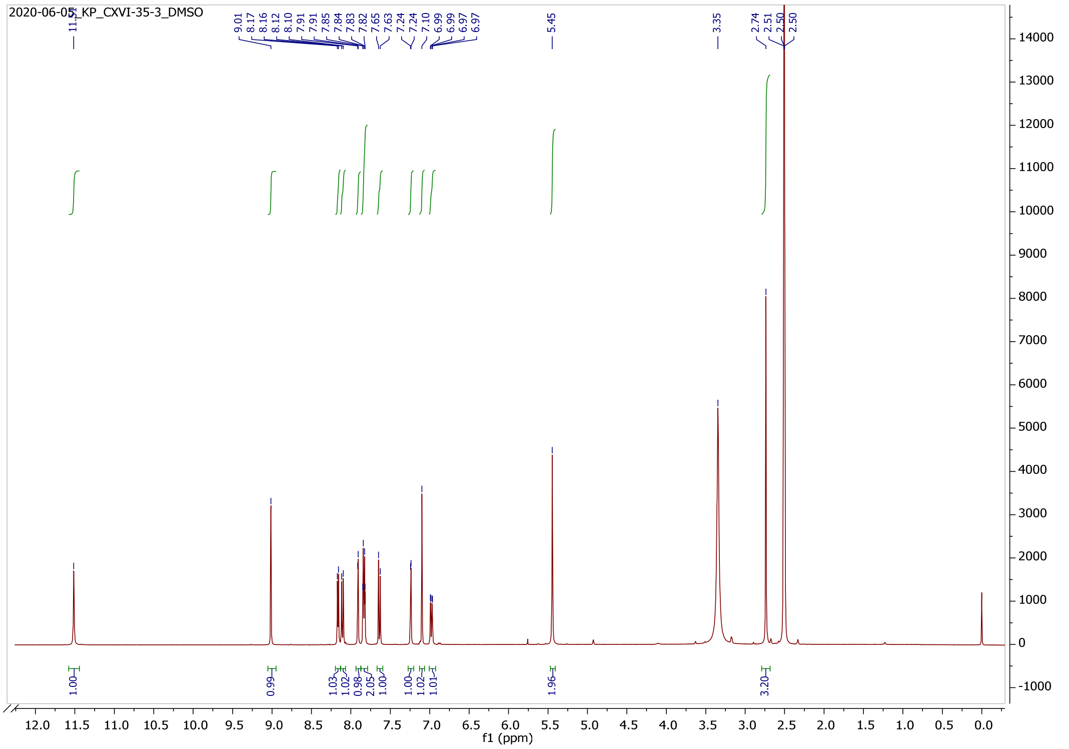
2020-06-05_KP_CXVI-35-3_DMSO
11.57
9.01
8.17
8.16
8.12
8.10
7.91
7.91
7.85
7.84
7.83
7.82
7.65
7.63
7.24
7.24
7.10
6.99
6.99
6.97
6.97
5.45
3.35
2.74
2.51
2.50
2.50
1.00
0.99
1.03
1.02
0.98
2.05
1.00
1.00
1.02
1.01
1.96
3.20
f1 (ppm)

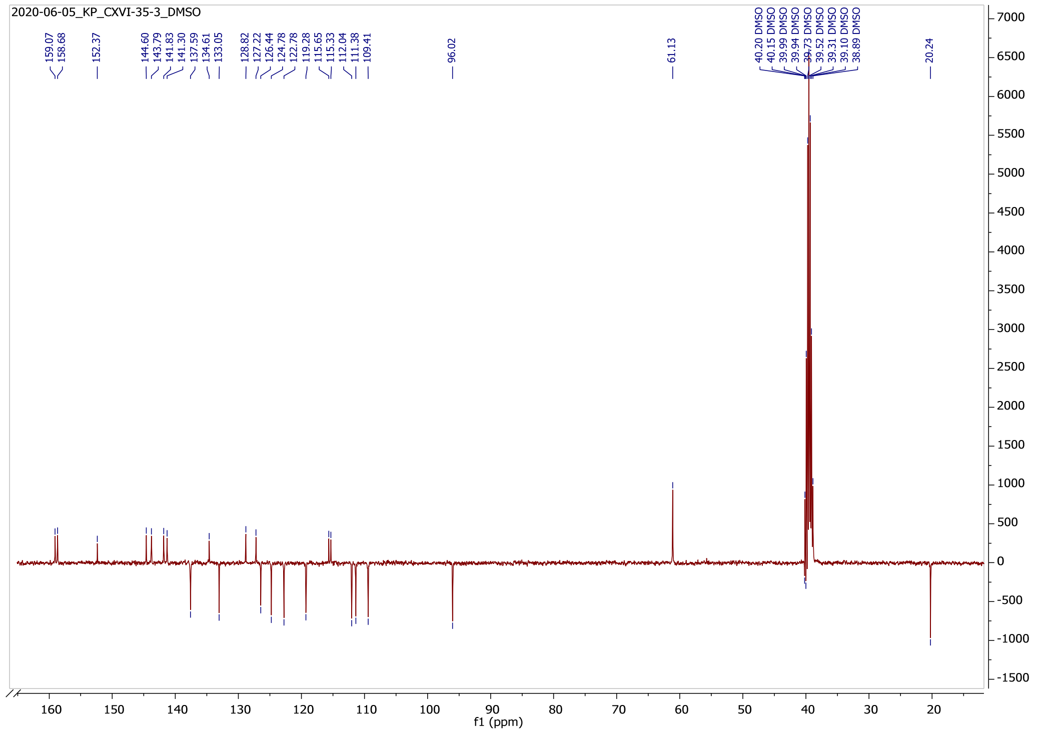
2020-06-05_KP_CXVI-35-3_DMSO
159.07
158.68
152.37
144.60
143.79
141.83
141.30
137.59
134.61
133.05
128.82
127.22
126.44
124.78
122.78
119.28
115.65
115.33
112.04
111.38
109.41
96.02
61.13
40.20 DMSO
40.15 DMSO
39.99 DMSO
39.94 DMSO
39.73 DMSO
39.52 DMSO
39.31 DMSO
39.10 DMSO
38.89 DMSO
20.24
f1 (ppm)

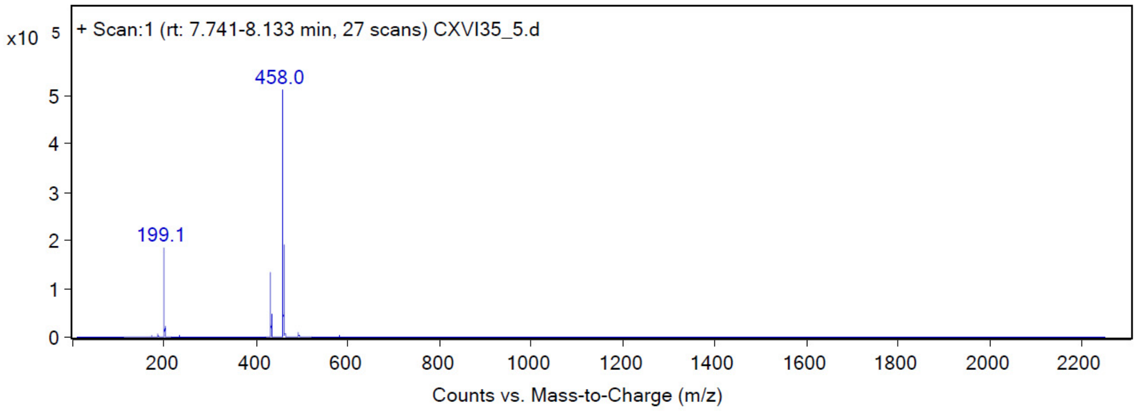

x10 5
+ Scan:1 (rt: 7.741-8.133 min, 27 scans) CXVI35_5.d
458.0
199.1
Counts vs. Mass-to-Charge (m/z)

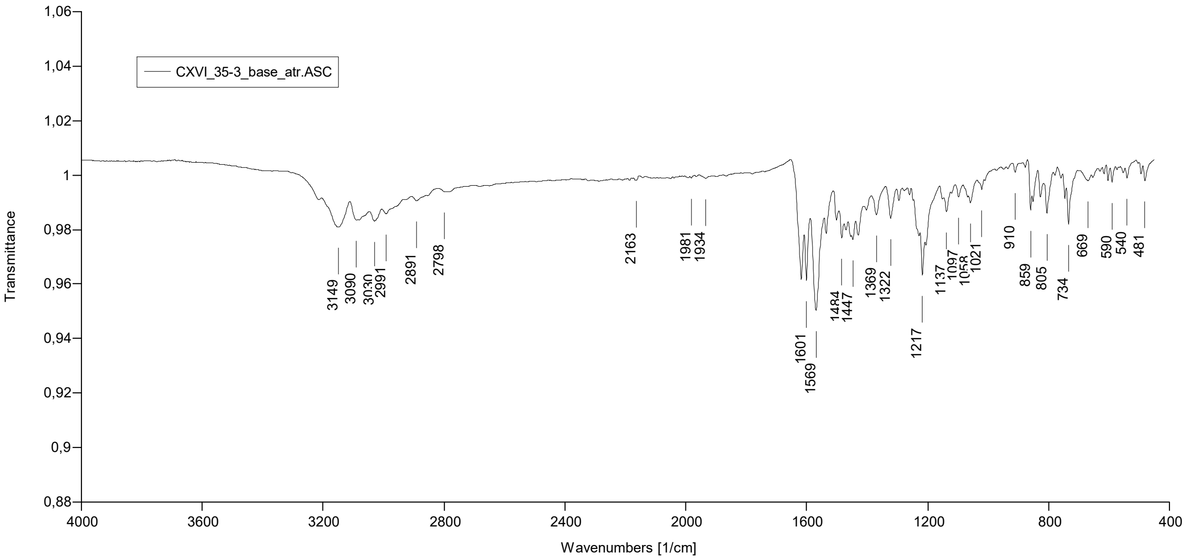

CXVI_35-3_base_atr.ASC
Transmittance
Wavenumbers [1/cm]
3149
3090
3030
2991
2891
2798
2163
1981
1934
1601
1569
1484
1447
1369
1322
1217
1137
1097
1058
1021
910
859
805
734
669
590
540
481

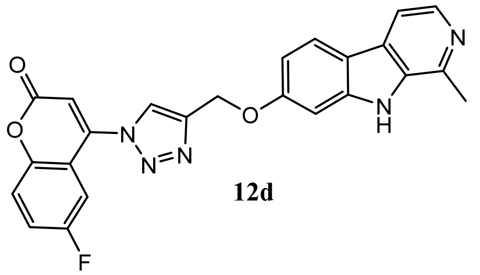
O
O
N
N
N
O
N
H
N
F

12d

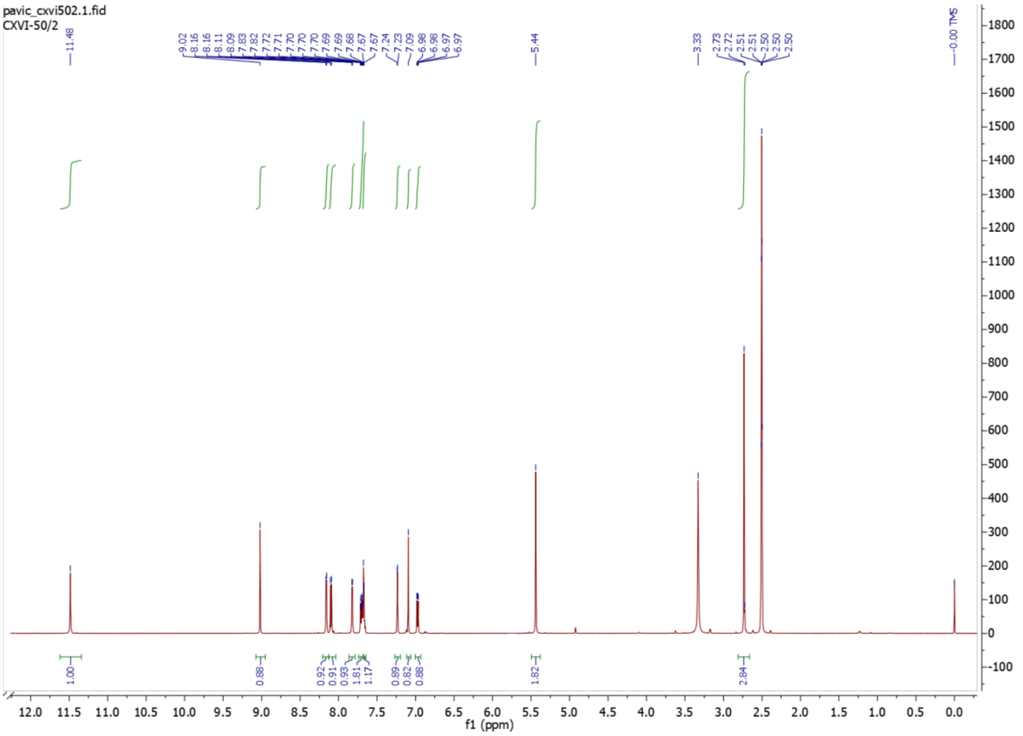
pavic_cxvi502.1.fid
CXVI-50/2
11.48
9.02
8.16
8.11
8.09
7.83
7.82
7.72
7.71
7.70
7.70
7.70
7.69
7.69
7.68
7.67
7.67
7.24
7.23
7.09
6.98
6.98
6.97
6.97
5.44
3.33
2.73
2.72
2.51
2.50
2.50
2.50
0.00 TMS
1.00
0.88
0.92
0.91
0.93
1.81
1.17
0.89
0.82
0.88
1.82
2.84
f1 (ppm)

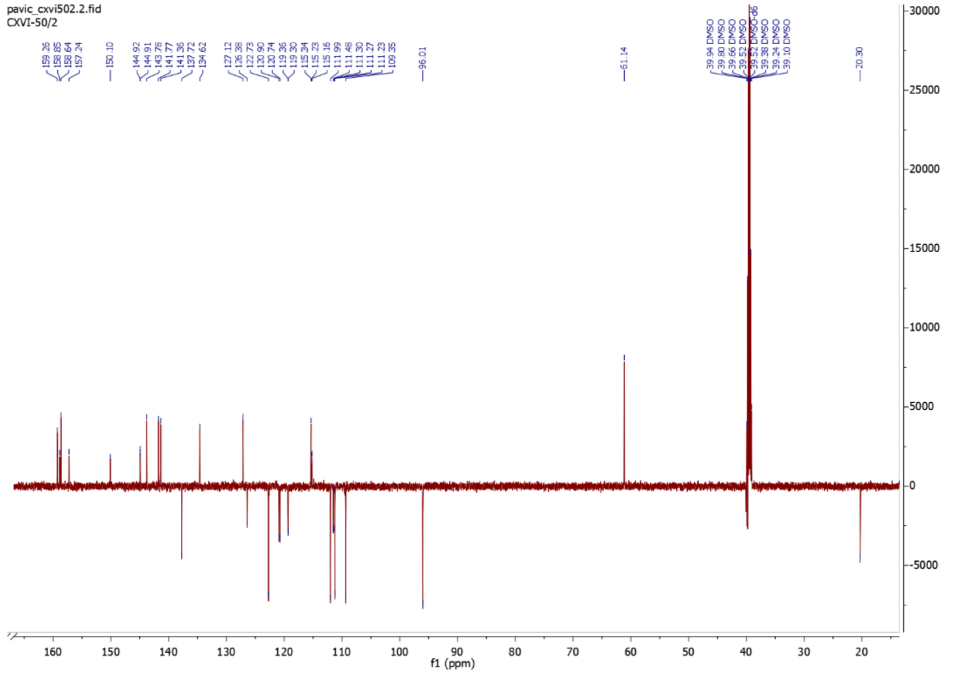
pavic_cxvi502.2.fid
CXVI-50/2
159.26
158.85
158.64
157.24
150.10
144.92
144.91
143.78
141.77
141.36
137.72
134.62
127.12
126.38
122.73
120.90
120.74
119.36
119.30
115.34
115.23
115.16
111.99
111.48
111.30
111.27
111.23
109.35
96.01
61.14
39.94 DMSO
39.80 DMSO
39.66 DMSO
39.52 DMSO
39.52 DMSO-d6
39.38 DMSO
39.24 DMSO
39.10 DMSO
20.30
f1 (ppm)

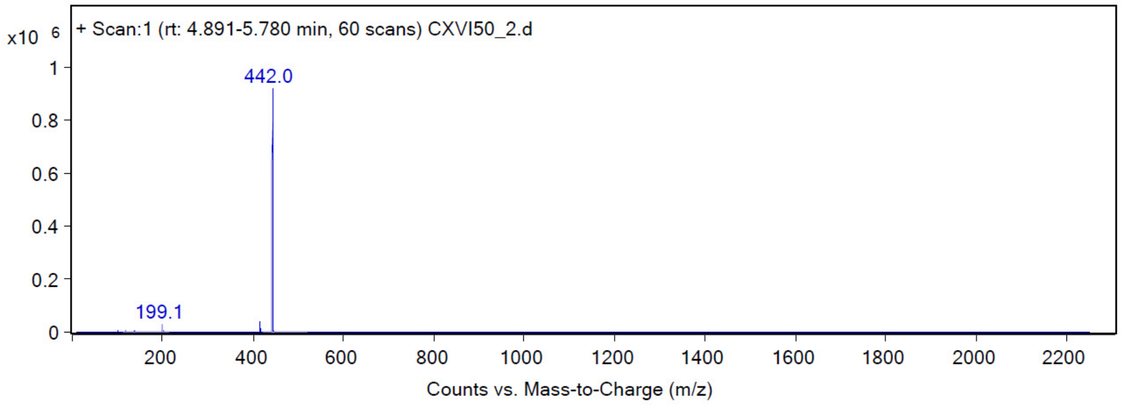

x10 6
+ Scan:1 (rt: 4.891-5.780 min, 60 scans) CXVI50_2.d
442.0
199.1
Counts vs. Mass-to-Charge (m/z)

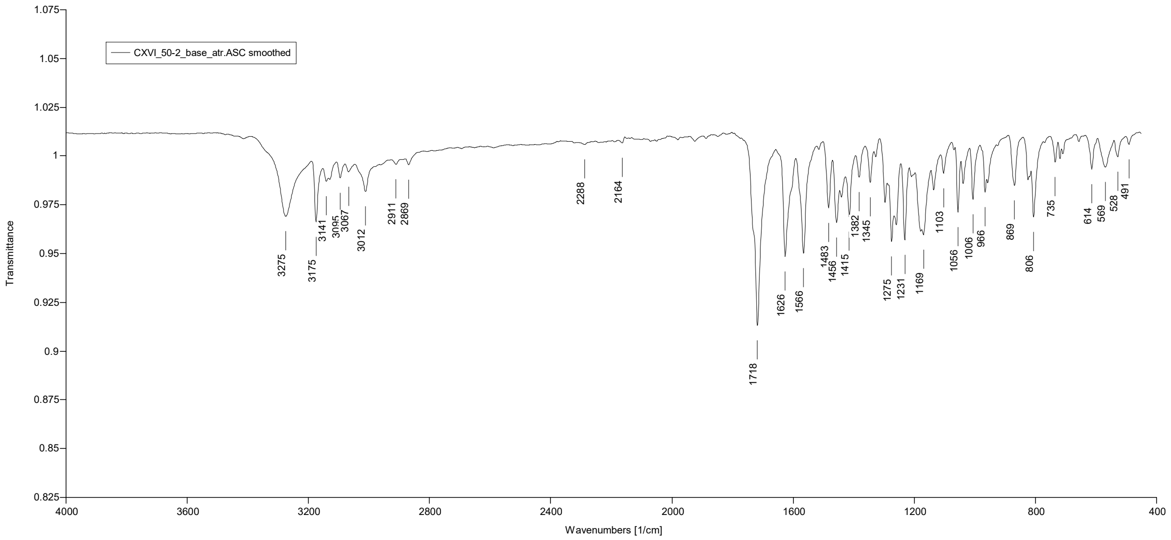

CXVI_50-2_base_atr.ASC smoothed
Transmittance
Wavenumbers [1/cm]
3275
3175
3141
3095
3067
3012
2911
2869
2288
2164
1718
1626
1566
1483
1456
1415
1382
1345
1275
1231
1169
1103
1056
1006
966
869
806
735
614
569
528
491

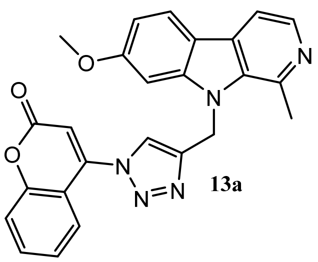

13a

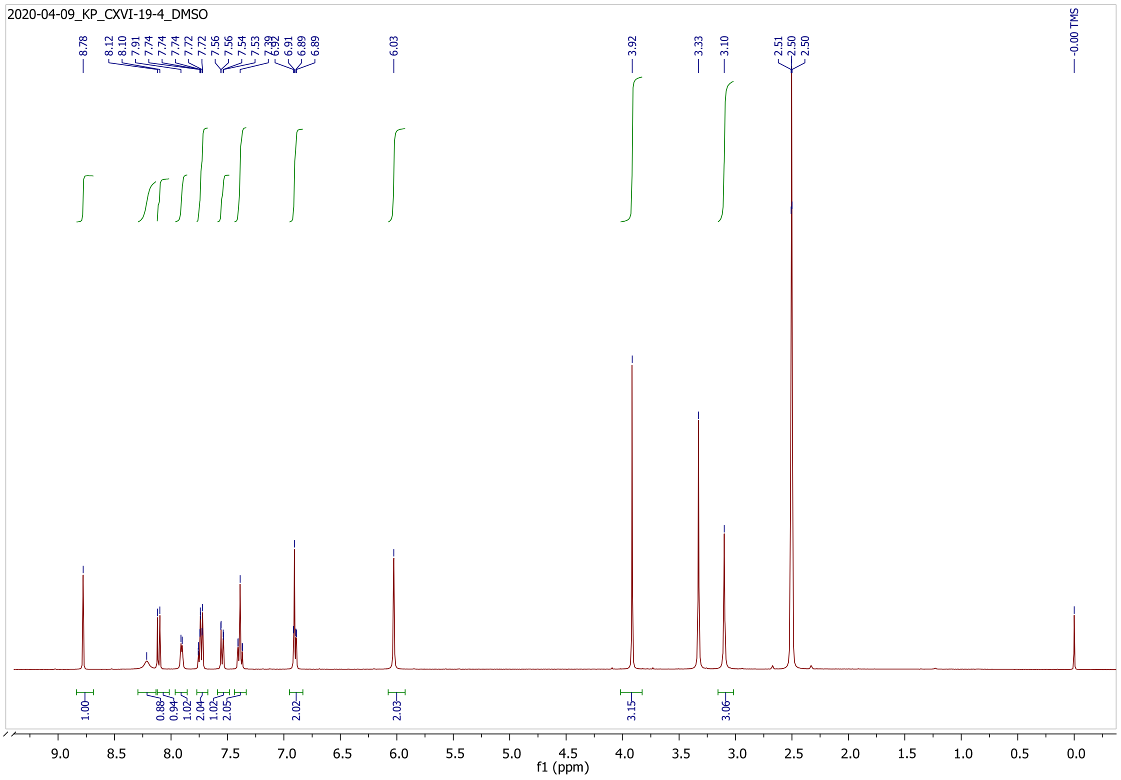

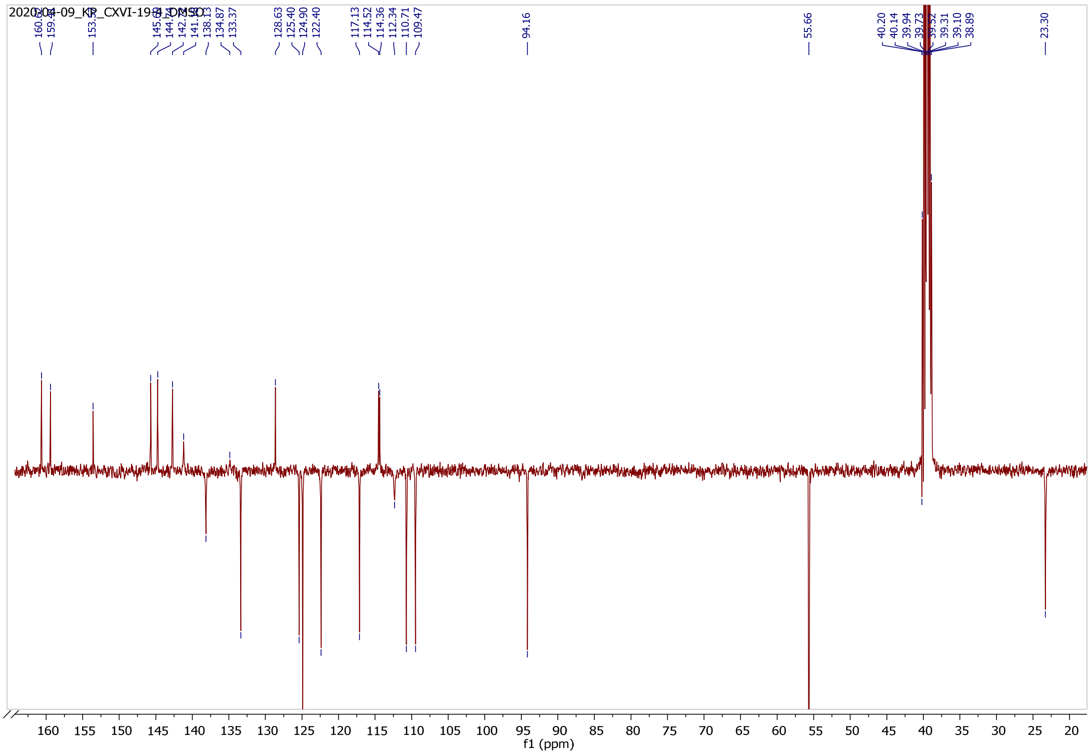

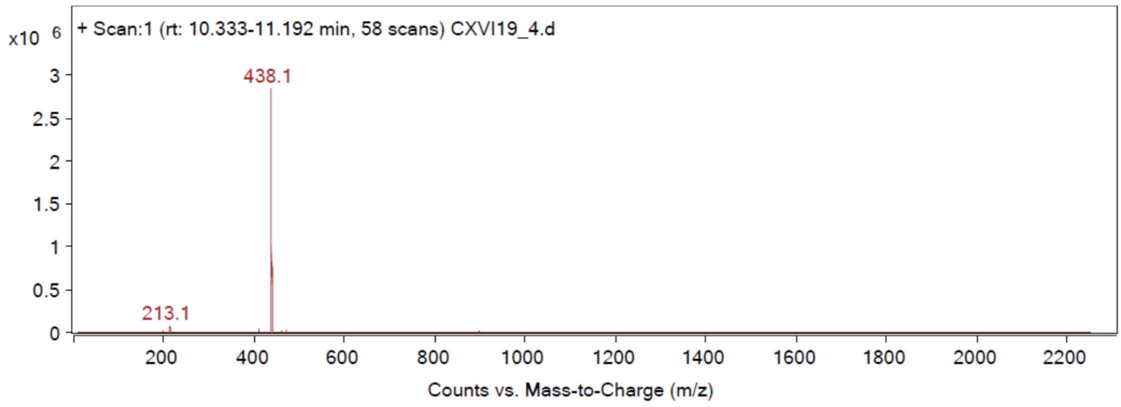

+ Scan:1 (rt: 10.333-11.192 min, 58 scans) CXVI19_4.d
x10 6
438.1
213.1
Counts vs. Mass-to-Charge (m/z)

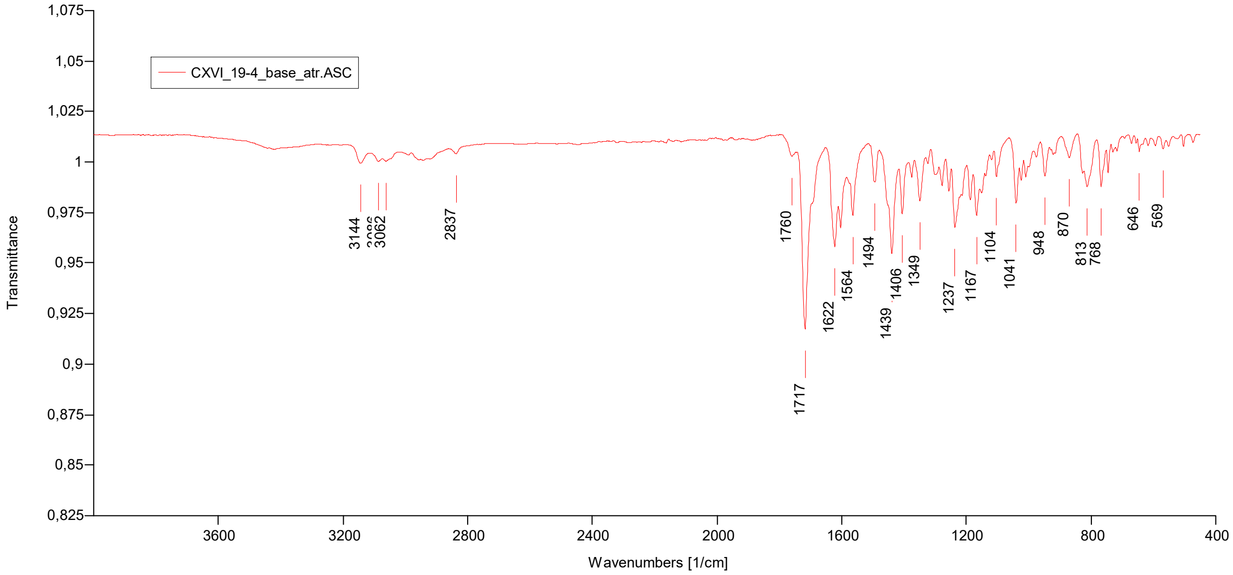

CXVI_19-4_base_atr.ASC
Transmittance
Wavenumbers [1/cm]
3144
3062
2837
1760
1717
1622
1564
1494
1439
1406
1349
1237
1167
1104
1041
948
870
813
768
646
569

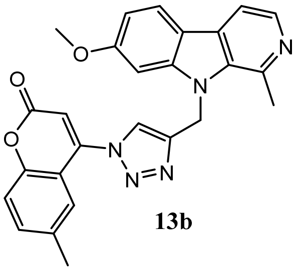
O
N
N
N
N
N
O
O

13b

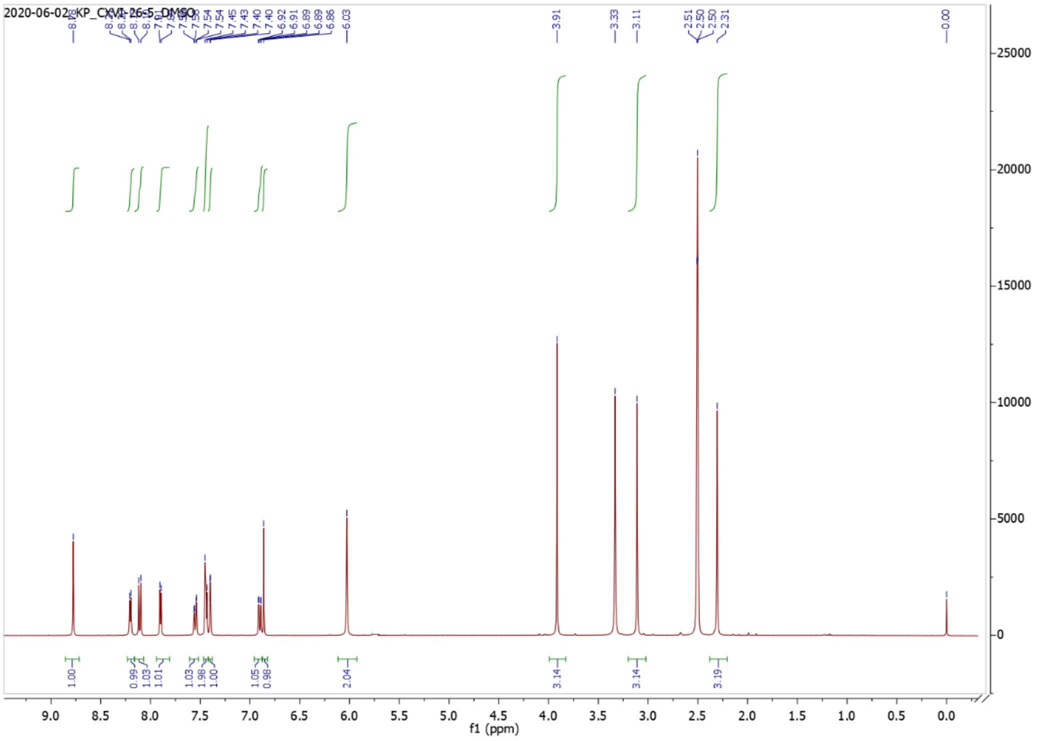
2020-06-02_KP_CXVI-26-5_DMSO
7.54
7.54
7.45
7.43
7.40
7.40
6.92
6.91
6.89
6.89
6.86
6.03
3.91
3.33
3.11
2.51
2.50
2.50
2.31
0.00
1.00
0.99
1.03
1.01
1.03
1.98
1.00
1.05
0.98
2.04
3.14
3.14
3.19
25000
20000
15000
10000
5000
0
9.0
8.5
8.0
7.5
7.0
6.5
6.0
5.5
5.0
4.5
4.0
3.5
3.0
2.5
2.0
1.5
1.0
0.5
0.0
f1 (ppm)

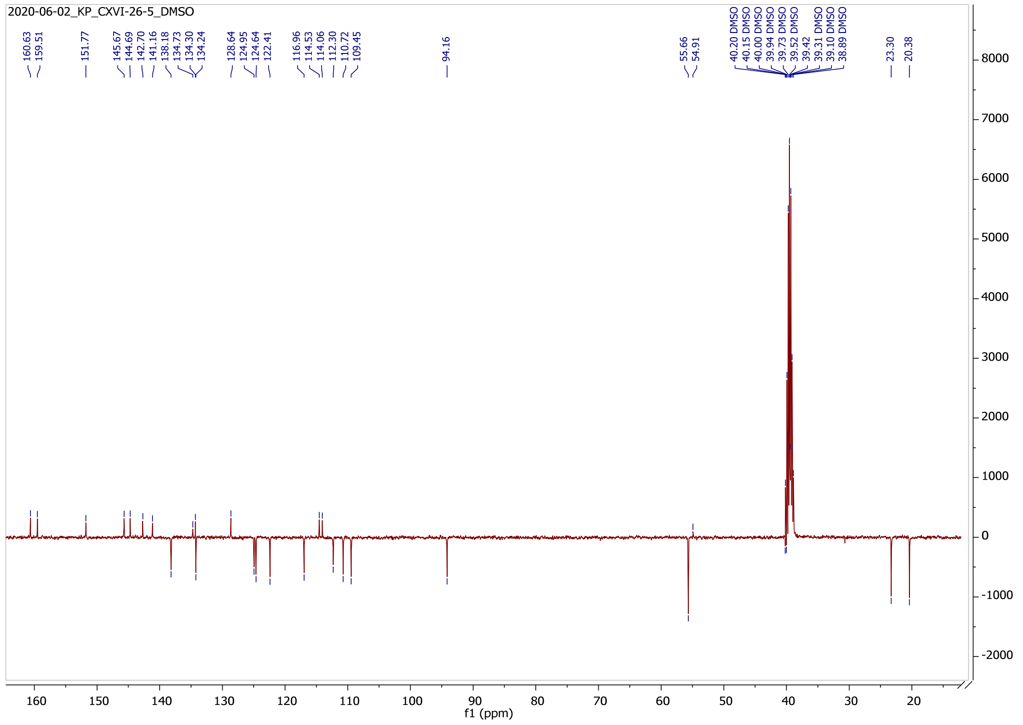
2020-06-02_KP_CXVI-26-5_DMSO
160.63
159.51
151.77
145.67
144.69
142.70
141.16
138.18
134.73
134.30
134.24
128.64
124.95
124.64
122.41
116.96
114.53
114.06
112.30
110.72
109.45
94.16
55.66
54.91
40.20 DMSO
40.15 DMSO
40.00 DMSO
39.94 DMSO
39.73 DMSO
39.52 DMSO
39.42
39.31 DMSO
39.10 DMSO
38.89 DMSO
23.30
20.38
8000
7000
6000
5000
4000
3000
2000
1000
0
-1000
-2000
160
150
140
130
120
110
100
90
80
70
60
50
40
30
20
f1 (ppm)

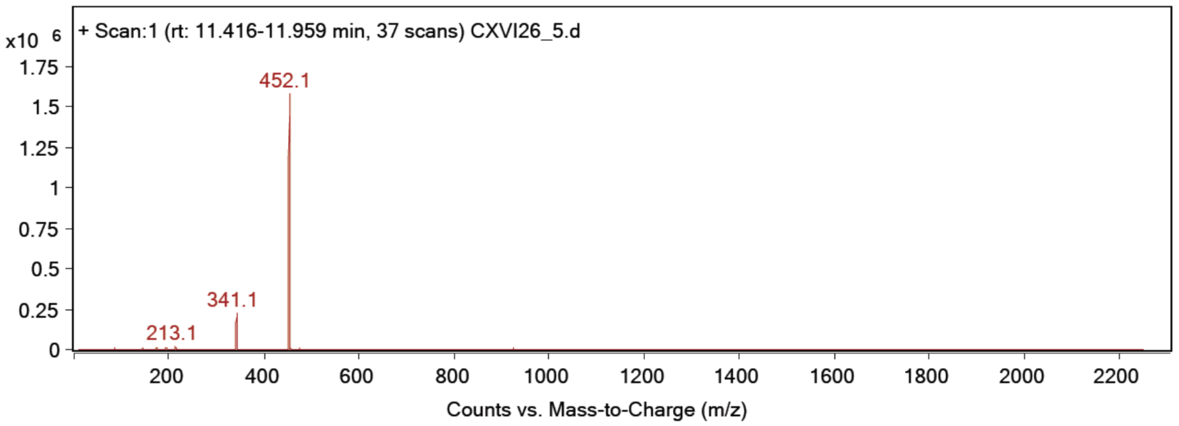
x10 6
+ Scan:1 (rt: 11.416-11.959 min, 37 scans) CXVI26_5.d
452.1
341.1
213.1
Counts vs. Mass-to-Charge (m/z)

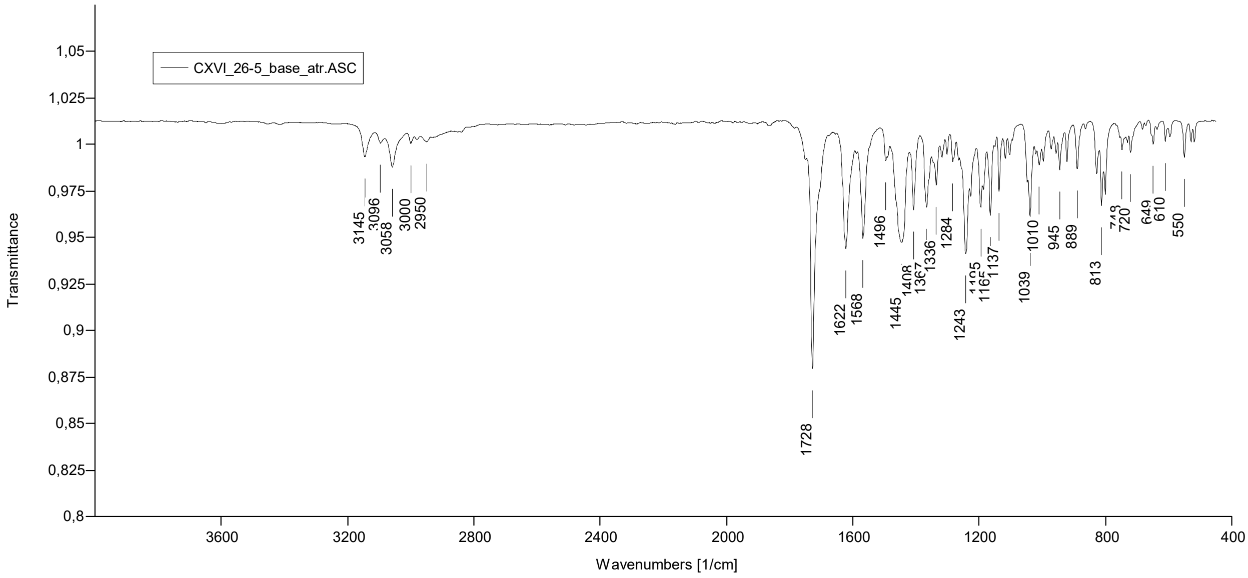
CXVI_26-5_base_atr.ASC
Transmittance
Wavenumbers [1/cm]
3145
3096
3058
3000
2950
1728
1622
1568
1496
1445
1336
1284
1243
1137
1039
1010
945
889
813
720
649
610
550

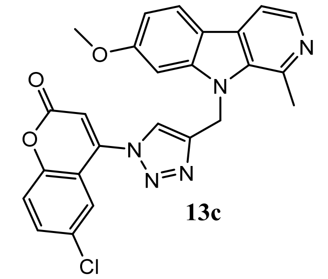
O
O
N
N
N
N
N
O
Cl

13c

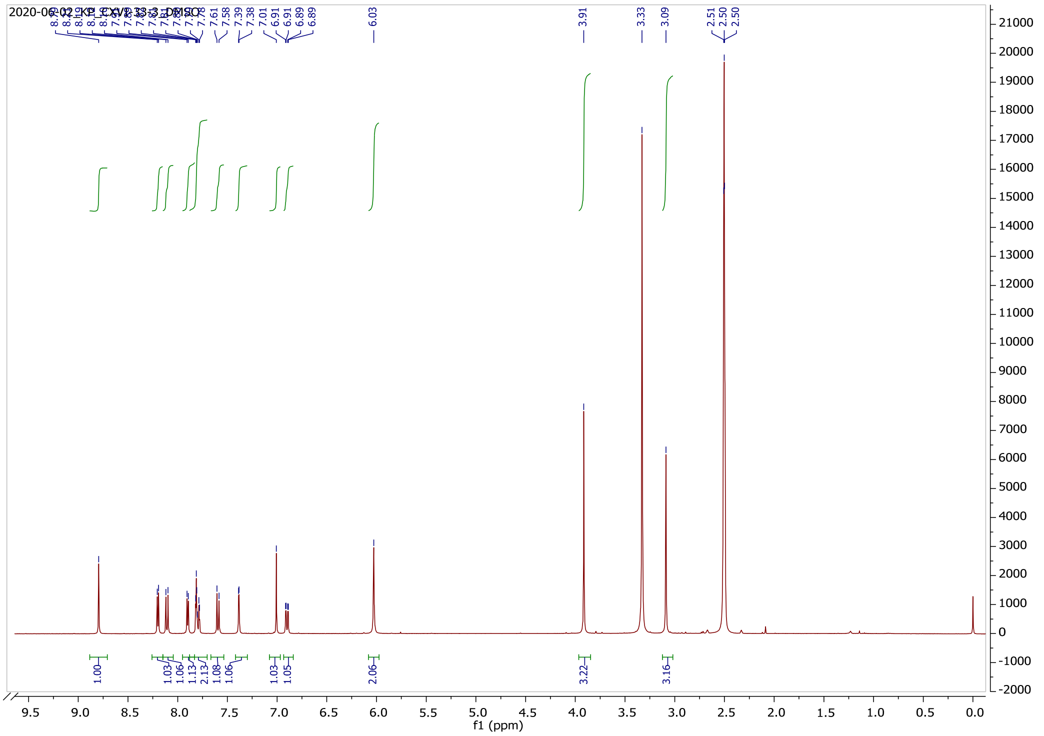
7.61
7.58
7.39
7.38
7.01
6.91
6.91
6.89
6.89
6.03
3.91
3.33
3.09
2.51
2.50
2.50
1.00
1.03
1.06
1.13
2.13
1.08
1.06
1.03
1.05
2.06
3.22
3.16
f1 (ppm)

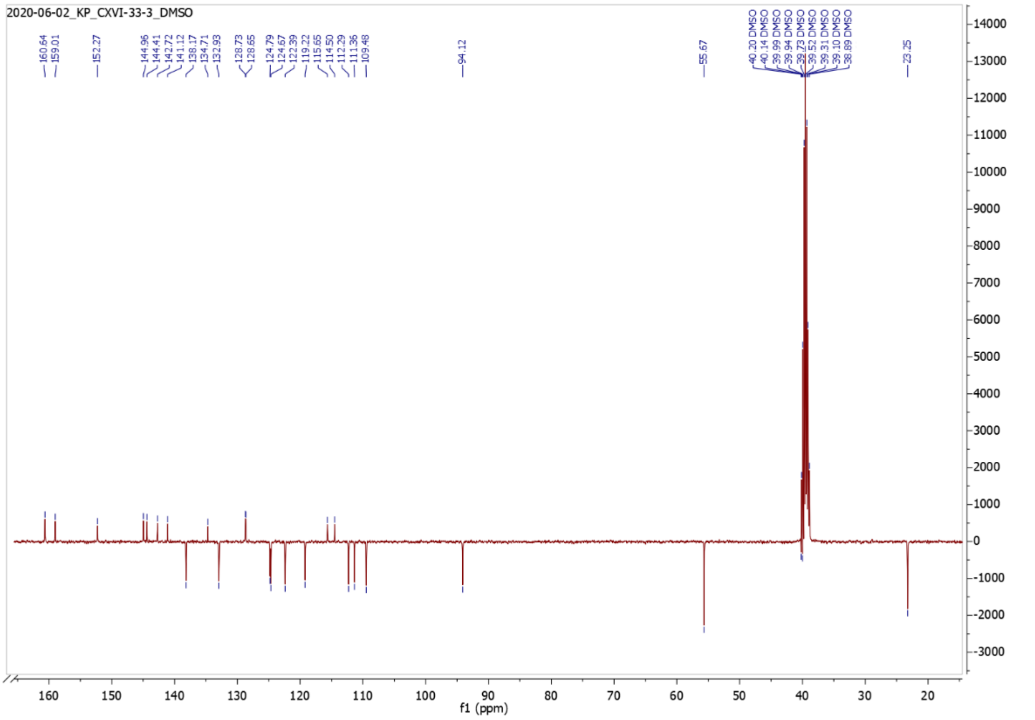
2020-06-02_KP_CXVI-33-3_DMSO
160.64
159.01
152.27
144.96
144.41
142.72
141.12
138.17
134.71
132.93
128.73
128.65
124.79
124.67
122.39
119.22
115.65
114.50
112.29
111.36
109.48
94.12
55.67
40.20 DMSO
40.14 DMSO
39.99 DMSO
39.94 DMSO
39.73 DMSO
39.52 DMSO
39.31 DMSO
39.10 DMSO
38.89 DMSO
23.25
f1 (ppm)

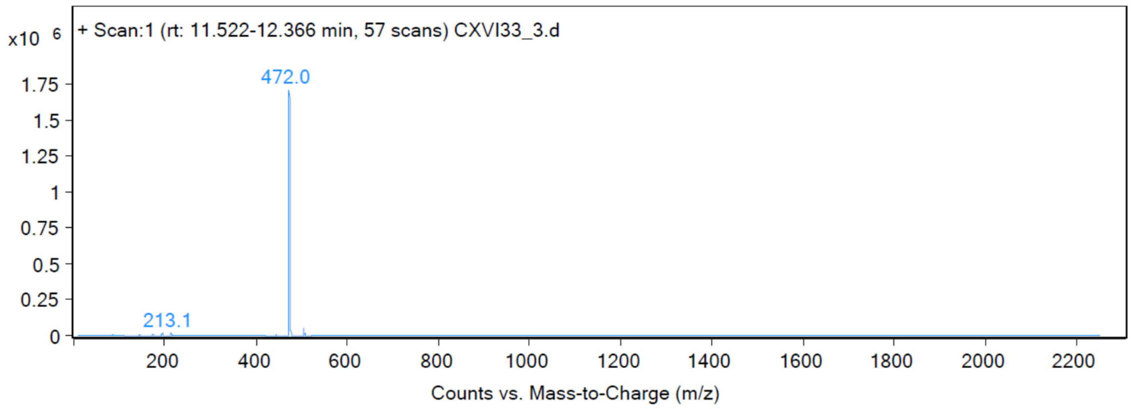
x10 6
+ Scan:1 (rt: 11.522-12.366 min, 57 scans) CXVI33_3.d
472.0
213.1
Counts vs. Mass-to-Charge (m/z)

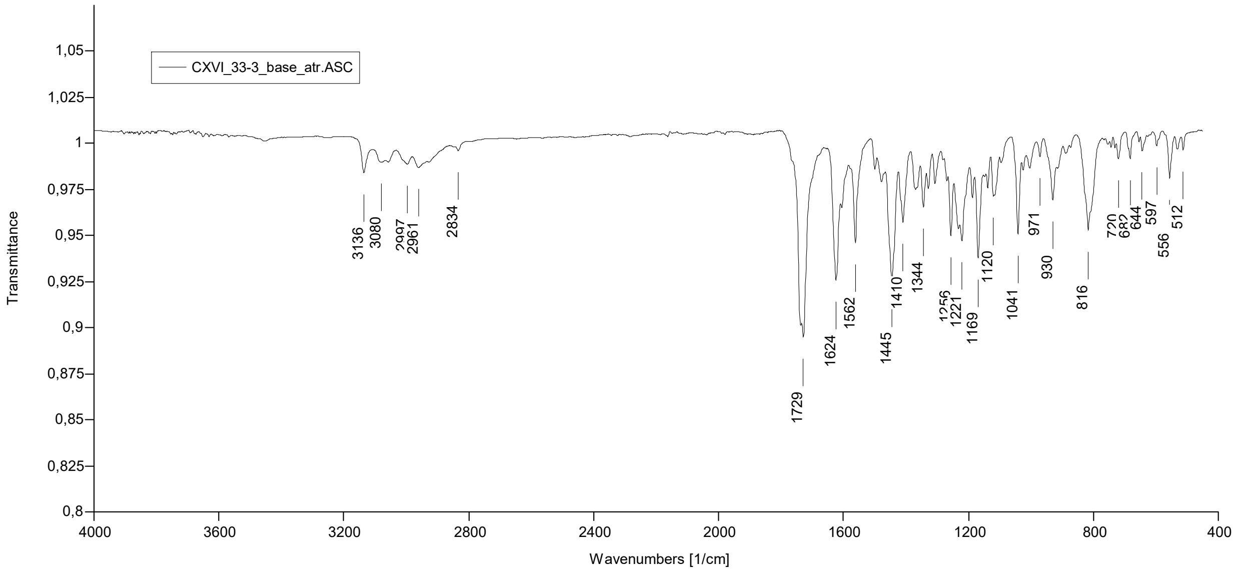
CXVI_33-3_base_atr.ASC
Transmittance
Wavenumbers [1/cm]
3136
3080
2997
2961
2834
1729
1624
1562
1445
1410
1344
1256
1221
1169
1120
1041
971
930
816
720
682
644
597
556
512

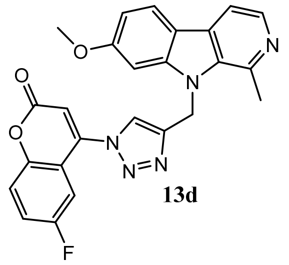

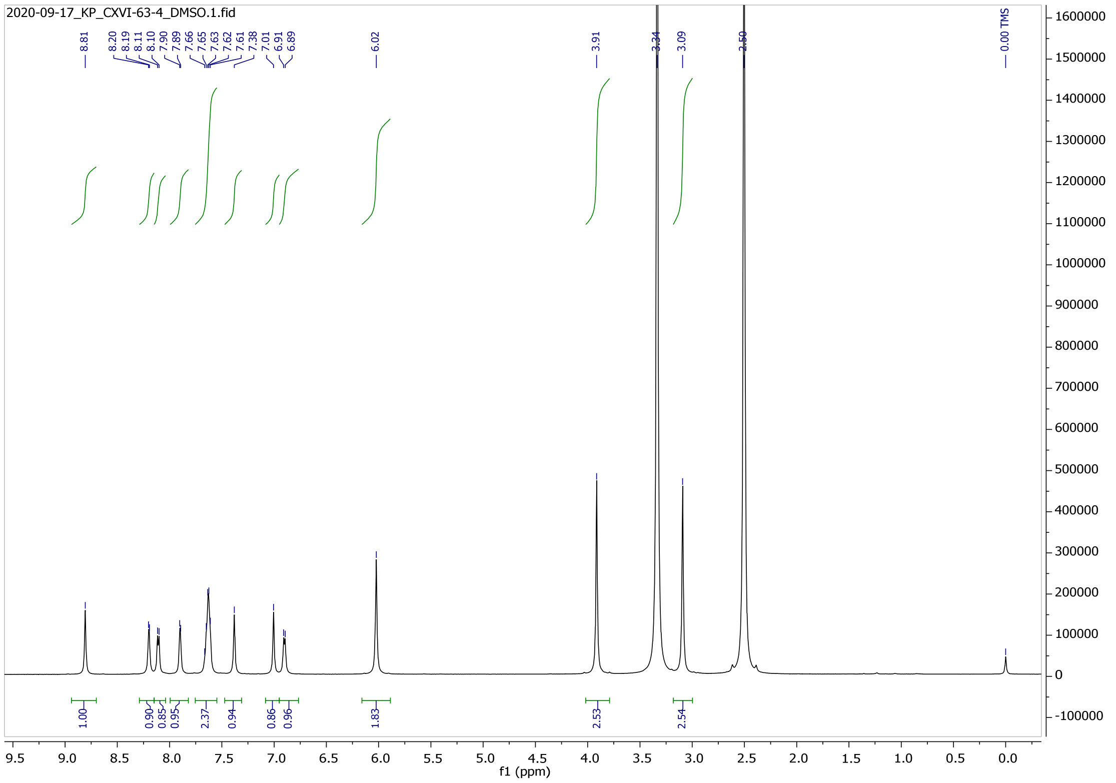

2020-09-17_KP_CXVI-63-4_DMSO.1.fid
f1 (ppm)

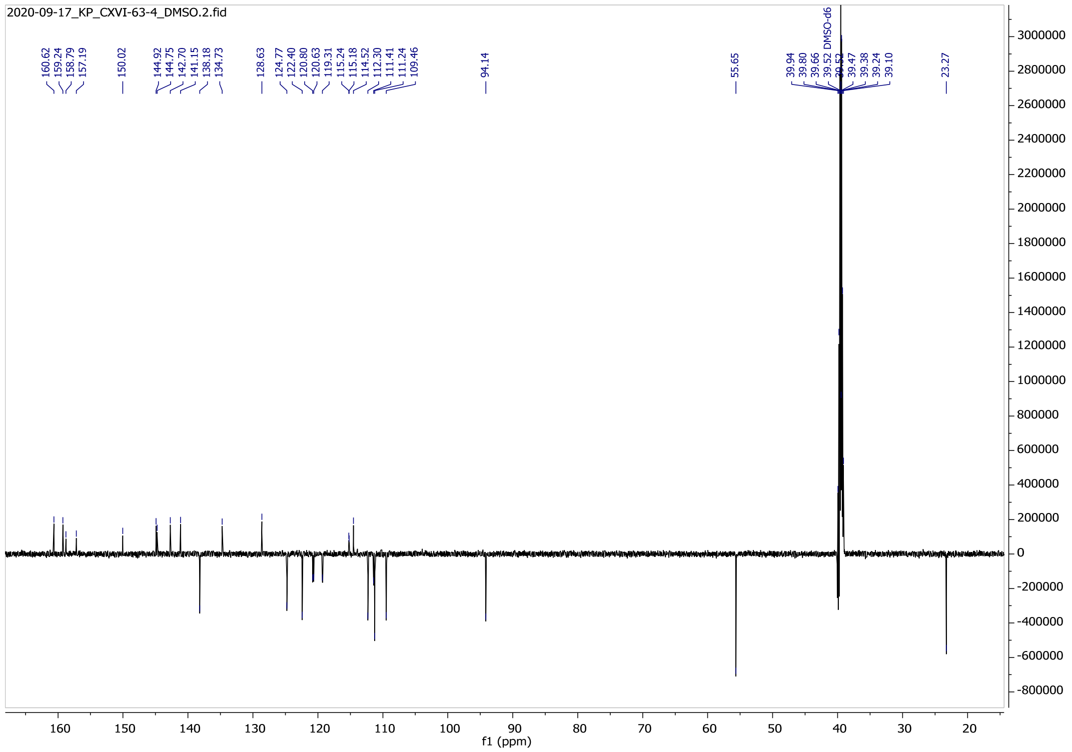

2020-09-17_KP_CXVI-63-4_DMSO.2.fid
f1 (ppm)

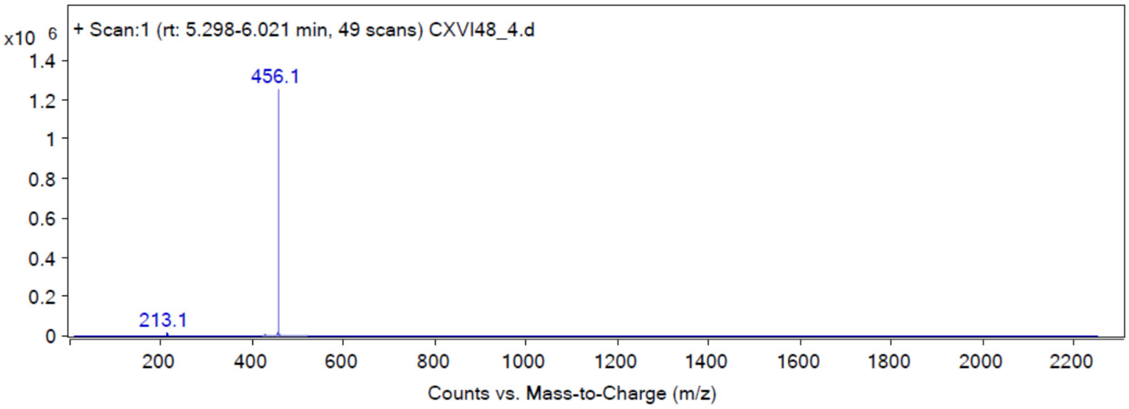

+ Scan:1 (rt: 5.298-6.021 min, 49 scans) CXVI48_4.d
x10 6
456.1
213.1
Counts vs. Mass-to-Charge (m/z)

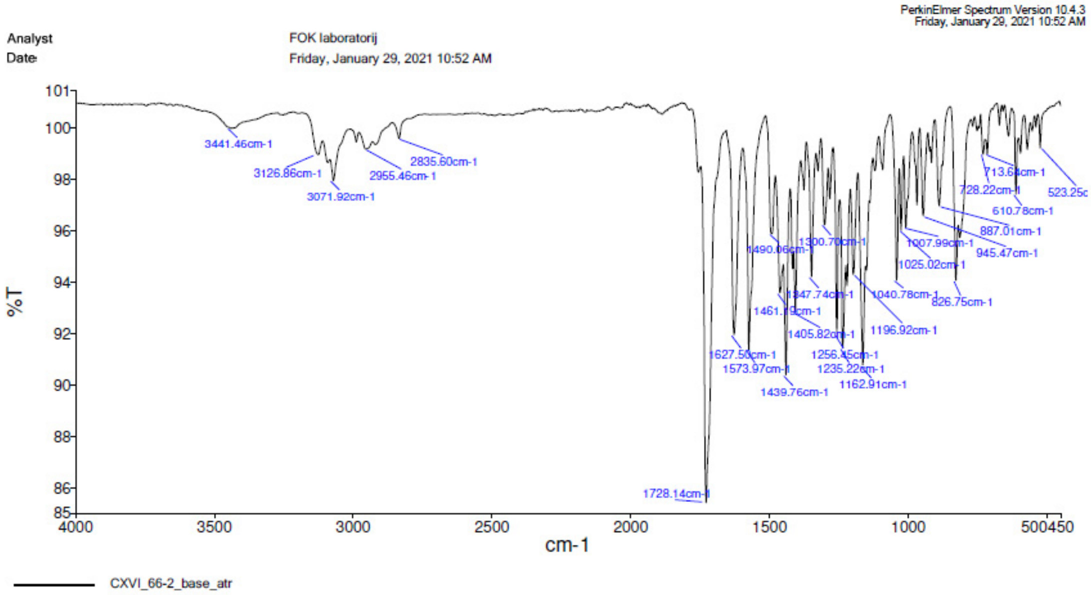

PerkinElmer Spectrum Version 10.4.3
Friday, January 29, 2021 10:52 AM
Analyst
Date
FOK laboratorij
Friday, January 29, 2021 10:52 AM
%T
cm-1
3441.46cm-1
3126.86cm-1
3071.92cm-1
2955.46cm-1
2835.60cm-1
1728.14cm-1
1627.50cm-1
1573.97cm-1
1490.06cm-1
1461.19cm-1
1439.76cm-1
1405.82cm-1
1347.74cm-1
1300.70cm-1
1256.45cm-1
1235.22cm-1
1196.92cm-1
1162.91cm-1
1040.78cm-1
1025.02cm-1
1007.99cm-1
945.47cm-1
887.01cm-1
826.75cm-1
728.22cm-1
713.64cm-1
610.78cm-1
CXVI_66-2_base_atr
